# Deciphering Mineral Homeostasis in Barley Seed Transfer Cells at Transcriptional Level

**DOI:** 10.1371/journal.pone.0141398

**Published:** 2015-11-04

**Authors:** Behrooz Darbani, Shahin Noeparvar, Søren Borg

**Affiliations:** 1 Department of Molecular Biology and Genetics, Research Centre Flakkebjerg, Aarhus University, Slagelse, Denmark; 2 Department of Plant and Environmental Sciences, University of Copenhagen, Frederiksberg, Denmark; INRA, FRANCE

## Abstract

In addition to the micronutrient inadequacy of staple crops for optimal human nutrition, a global downtrend in crop-quality has emerged from intensive breeding for yield. This trend will be aggravated by elevated levels of the greenhouse gas carbon dioxide. Therefore, crop biofortification is inevitable to ensure a sustainable supply of minerals to the large part of human population who is dietary dependent on staple crops. This requires a thorough understanding of plant-mineral interactions due to the complexity of mineral homeostasis. Employing RNA sequencing, we here communicate transfer cell specific effects of excess iron and zinc during grain filling in our model crop plant barley. Responding to alterations in mineral contents, we found a long range of different genes and transcripts. Among them, it is worth to highlight the auxin and ethylene signaling factors *Arf*s, *Abcb*s, *Cand*1, *Hps*4, *Hac*1, *Ecr*1, and *Ctr*1, diurnal fluctuation components *Sdg*2, *Imb*1, *Lip*1, and *Phy*C, retroelements, sulfur homeostasis components *Amp*1, *Hmt*3, *Eil*3, and *Vip*1, mineral trafficking components *Med*16, *Cnnm*4, *Aha*2, *Clpc*1, and *Pcbp*s, and vacuole organization factors *Ymr*155W, *Rab*G3F, *Vps*4, and *Cbl*3. Our analysis introduces new interactors and signifies a broad spectrum of regulatory levels from chromatin remodeling to intracellular protein sorting mechanisms active in the plant mineral homeostasis. The results highlight the importance of storage proteins in metal ion toxicity-resistance and chelation. Interestingly, the protein sorting and recycling factors *Exo*c7, *Cdc*1, *Sec*23A, and *Rab*11A contributed to the response as well as the polar distributors of metal-transporters ensuring the directional flow of minerals. Alternative isoform switching was found important for plant adaptation and occurred among transcripts coding for identical proteins as well as transcripts coding for protein isoforms. We also identified differences in the alternative-isoform preference between the treatments, indicating metal-affinity shifts among isoforms of metal transporters. Most important, we found the zinc treatment to impair both photosynthesis and respiration. A wide range of transcriptional changes including stress-related genes and negative feedback loops emphasize the importance to withhold mineral contents below certain cellular levels which otherwise might lead to agronomical impeding side-effects. By illustrating new mechanisms, genes, and transcripts, this report provides a solid platform towards understanding the complex network of plant mineral homeostasis.

## Introduction

Recognized for long, iron and zinc are essential cellular elements of importance to public health [1, 2]. However, the human diet-dependency on staple crops which are poor in minerals provokes inadequate intake of iron and zinc. Human micronutrient deficiency is a global health challenge and is categorized among the most important disease precursors. Iron and zinc deficiencies in plants are also agricultural challenges [3, 4]. Opposing to the higher levels of plant micronutrients is not only the artificial selection for higher yield but also the daily increasing levels of atmospheric carbon dioxide [5]. To combat micronutrient deficiency, we have to boost the micronutrient levels in crop varieties. The strategy although have some severe negative feedback loops resulted by metal ion-enhanced oxidative stress [2]. Therefore, it is very decisive to fully unravel the components of mineral homeostasis. Microarray transcript profiling has been used in the search for important components of mineral homeostasis. This introduced the heavy metal ATPase *Hma*3, cation diffusion facilitator 1, and nicotianamine synthase *Nas*3 as the responsible genes for zinc hyperaccumulation in *Arabidopsis halleri* [6]. Another cross-species comparison introduced the zinc transporter ZIP4, metal tolerance proteins MTP1 and 8, HMA3 and 4, iron regulated transporter IRT3, and natural resistance-associated macrophage protein NRAMP3 as well as lignin biosynthesis to be involved in metal hyperaccumulation by *Thlaspi caerulescens* [7]. Kobayashi et al. [8] highlighted the induction of methionine cycle and phytosiderophore biosynthesis genes under iron deficiency in rice. Mineral loading components of rice seeds have also been mapped to the members of *Zip*, Yellow stipe-like (*Ysl*), and *Nramp* gene families [9]. A comprehensive microarray study indicated the importance of iron for plastids and Fe-S cluster biosynthesis as well as detoxification mechanisms of metals like zinc under iron deficiency [10]. Moreover, global repression in photosynthesis related genes has been emphasized in iron depleted cyanobacteria [11]. Recently, RNA-Sequencing (RNA-Seq) has been applied to dissect plant mineral homeostasis [12, 13] and most interesting, recalled the repression of photosynthesis and tetrapyrrole biosynthesis related genes in iron deficient *Arabidopsis* [14].

In spite of our current knowledge, plant mineral homeostasis has yet to be fully explored. Utilizing microarray, we previously mapped the expression of diverse family members including *Zip*, *Nramp*, *Ysl*, *Hma*, *Mtp*, and Ca^2+^/H^+^-exchanger (*Cax*) genes to the transfer cells, aleurone layer, endosperm, and embryo of the barley grain [15]. However, microarray studies are limited to our knowledge of previously identified genes, they do not provide transcript splice-specific information, and suffer from narrow detection dynamic-range of expressional changes. To further extend, field-grown barley plants at the height of grain filling were subjected to foliar application of iron or zinc and a transcriptome-wide expression analysis was carried out for the transfer cells. Transfer cells bridge the maternal flux of materials towards the endosperm. As the edible part of grain, endosperm has been the target for micronutrient biofortification in crops. One advantage of the current study was the system-wide function and interaction analysis of the differentially expressed genes in order to identify highly potent candidates and strategies for biofortification. By using RNA-Seq, we not only provide experimental evidence to our recent homeostasis models [2] but also introduce a whole range of new aspects and components cooperating in plant mineral homeostasis. This includes different chromatin remodeling factors, auxin transporters, auxin and ethylene signaling components, clock genes, storage proteins, retroelements, and targeted sorting of protein. The results imply the involvement of different regulatory and non-regulatory mechanisms in fast response to the excess of micronutrients and recommend the bypassing of negative feedback loops and/or the use of upstream regulatory factors for biofortification crops.

## Results and Discussion

### Data analysis and statistics

**Fig 1:**
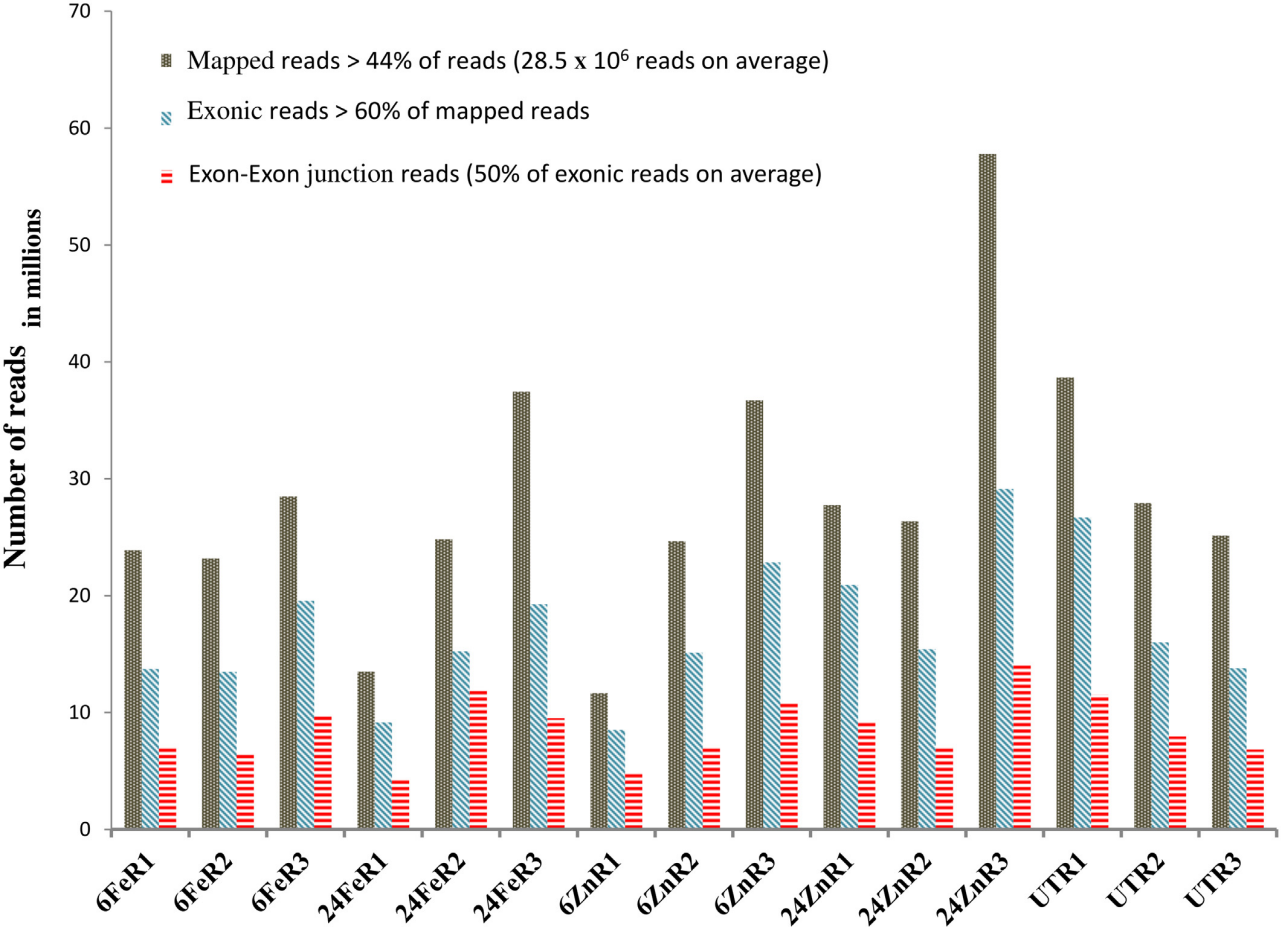
Mapping statistics. On average, 17 million exonic-reads were mapped per replicate. Fe and Zn represent iron and zinc treatments. Samples collected at 6 h and 24 h after the treatments are labeled by 6 and 24. UT is for untreated sample. R1, R2, and R3 represent biological replicates.

**Fig 2:**
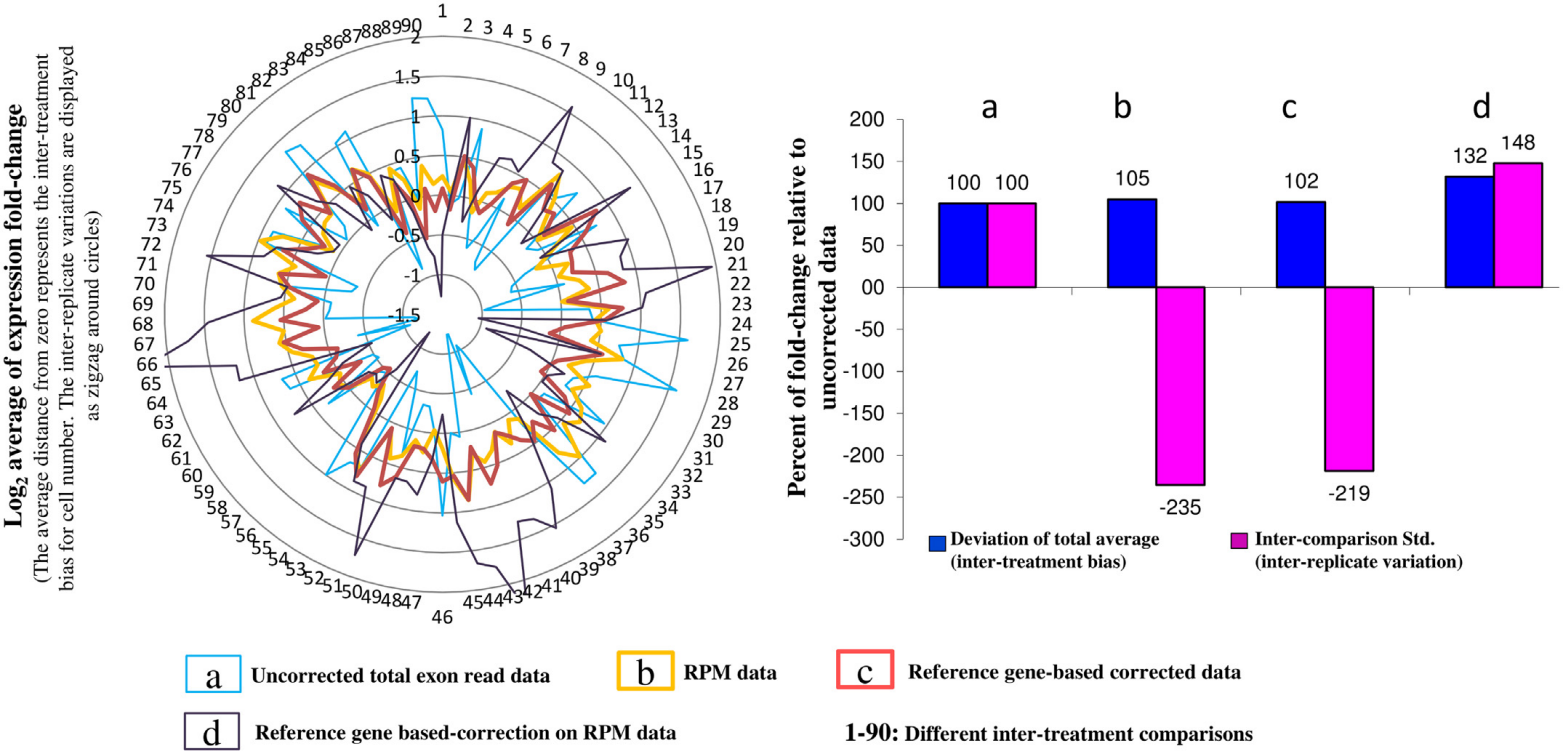
Bias structure analysis. The reference gene based-correction showed no advantage over the genome-wide correction. This can be explained by the single cell-type origin of the samples. We selected 18 reference genes with coefficient variations below 10% after analyzing 43 publicly-available barley microarray datasets including 891 samples and representing 22840 probes (S1 File). The inter-replicate variation and inter-treatment bias were calculated using the selected genes as described previously [17]. We considered all possible inter-treatment comparisons in our RNA-Seq data. There was no difference in inter-treatment bias among the single reference gene based-correction, exon read count based-correction measured as RPM, and uncorrected data. Sequential application of the corrections [17] was also not efficient. However, the exon read count based-correction was used for data analysis due to the large proportion of expelled inter-replicate variations. RPM: Read count Per Million Mapped reads, Std.: Standard deviation.

Transcriptome sequencing was performed to analyze the cellular response to foliar applications of iron and zinc in transfer cells of immature barley-grains. On average, 44.5% of the reads were mapped onto the barley genome (S1 Table). More than 50% of the 17.2 million exonic single-hit mapped reads evidenced exon-exon junctions per sample-replicate (Fig 1 and S1 Table). The reproducibility measure of slope of inter-replicate regression lines was improved from 0.86 to 0.95 after data correction. Data reproducibility was further checked using a sensitive measure of deviation from perfect reproducibility [16]. The averages of ratios' deviations improved from 55% in uncorrected data to 44% in corrected data. Reference gene-based correction showed no advantage compared to the total exon read count-based correction (Fig 2), revealed by bias structure analysis as introduced in [17]. This is expected from analyzing single cell-type samples.

**Fig 3:**
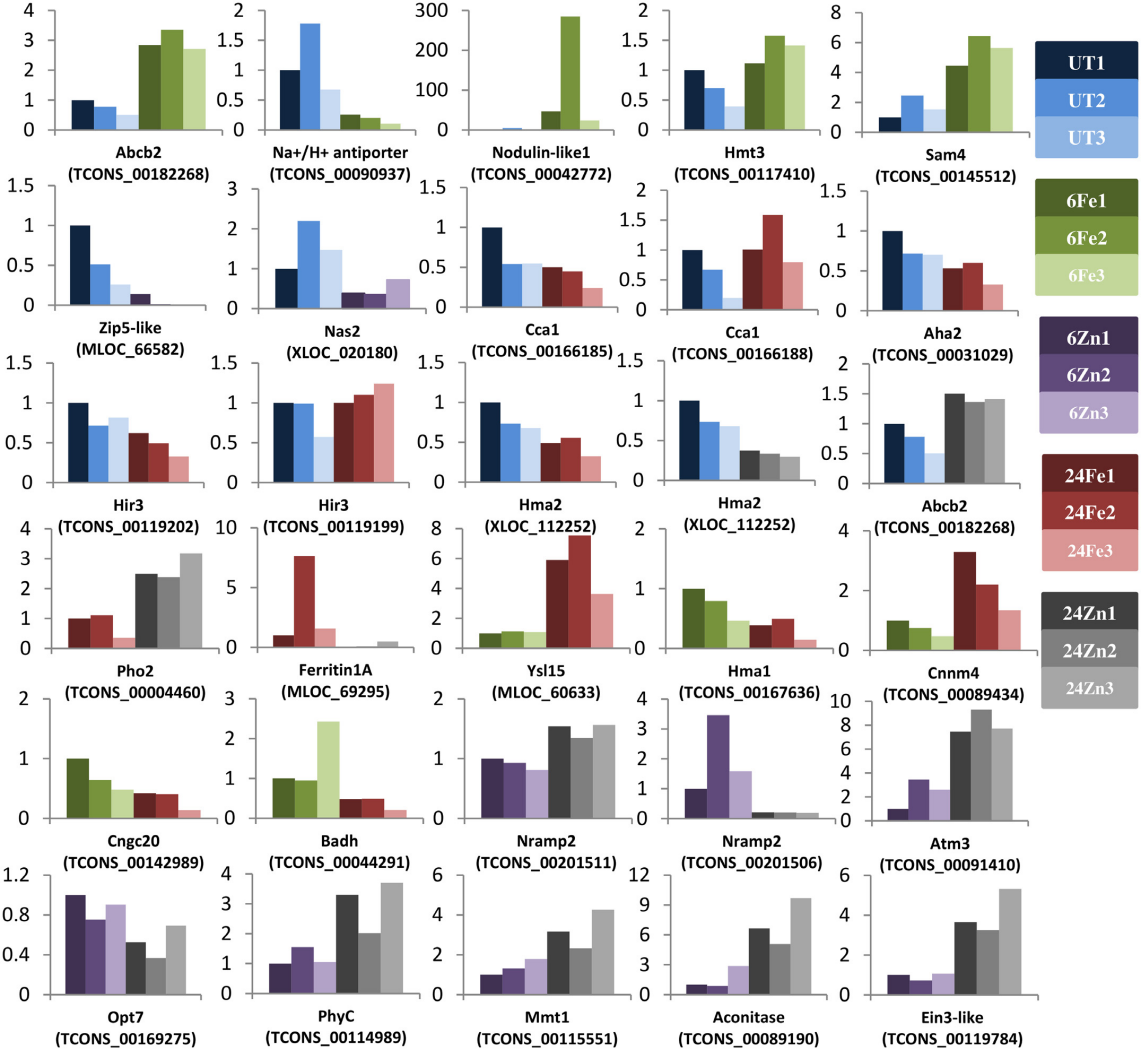
Real-Time PCR confirmed the expression changes. Gene- and transcript-specific primers were used to examine the expression changes of 30 selected events found by RNA-Seq. Accession numbers are shown below the gene names. The relative expression fold changes are shown individually. Except for the *Cca1* transcript TCONS_00166188, the expression changes were all in agreement with the RNA-Seq results (see S1 Fig). As the most stable gene (S1 File), the vacuolar ATP synthase was used to normalize the Real-Time PCR data. We also found identical expression patterns when normalizing the data to barley *Gadph* (S1 Fig). Three biological replicates (1–3) of samples including untreated sample (UT), 6 h after treatments (6Fe and 6Zn), and 24 h after treatments (24Fe and 24Zn) are shown in different colors. See S2 File for the functions of genes.

Statistical comparisons were carried out at both gene and transcript levels. On average, more than 90% of the differentially expressed genes and transcripts showed no significant changes at their transcript and gene levels, respectively. While this can be explained by switching, cumulative effects, and independent regulation of alternative transcript isoforms, it also reveals a complementary advantage of performing comparisons at both levels. Furthermore, we examined the expression changes of 30 differential expression events including genes and transcripts using Real-Time PCR. All changes, except one, were confirmed qualitatively (Fig 3).

Responding to the mineral supply, transcript isoform switching was a major regulatory mechanism and covered 19% of the differentially expressed transcripts (Table 1). Isoform switching in 18 genes (S3 File) including sucrose-phosphate synthase and indole-3-acetic acid-amido synthetase occurred among alternative transcripts coding for identical proteins. This mechanism can facilitate the utilization of one specific protein product through different antagonistic signaling pathways.

**Table 1:** Statistics of the comparisons.

Gene-level comparisons	Gene/Transcript	DE	Rep	Ind	IS	IS1	IS2
**24Fe/UT**	**16250**	**302**	**166**	**136**	**2**	**2**	**0**
**24Zn/UT**	**16171**	**106**	**85**	**21**	**0**	**0**	**0**
**6Zn/6Fe**	**16476**	**78**	**26**	**52**	**1**	**1**	**0**
**24Zn/24Fe**	**16758**	**105**	**95**	**10**	**2**	**1**	**1**
**Isoform-level comparisons**	**Gene/Transcript**	**DE**	**Rep**	**Ind**	**IS**	**IS1**	**IS2**
**24Fe/UT**	**18367**	**249**	**139**	**110**	**72**	**63**	**13**
**24Zn/UT**	**18696**	**484**	**239**	**245**	**113**	**94**	**19**
**6Zn/6Fe**	**18047**	**410**	**215**	**195**	**58**	**48**	**10**
**24Zn/24Fe**	**19389**	**585**	**296**	**289**	**86**	**71**	**11**

Number of significant changes (DE), repressed events (Rep), induced events (Ind), events with switched isoforms (IS), isoform switching among transcripts coding different protein isoforms (IS1), and isoform switching among transcripts only different in untranslated regions (IS2). UT: untreated samples, 6 & 24: samples of 6 h and 24 h after treatments, Fe & Zn: iron and zinc treatments.

### The effects of iron and zinc on upstream signaling pathways

**Fig 4:**
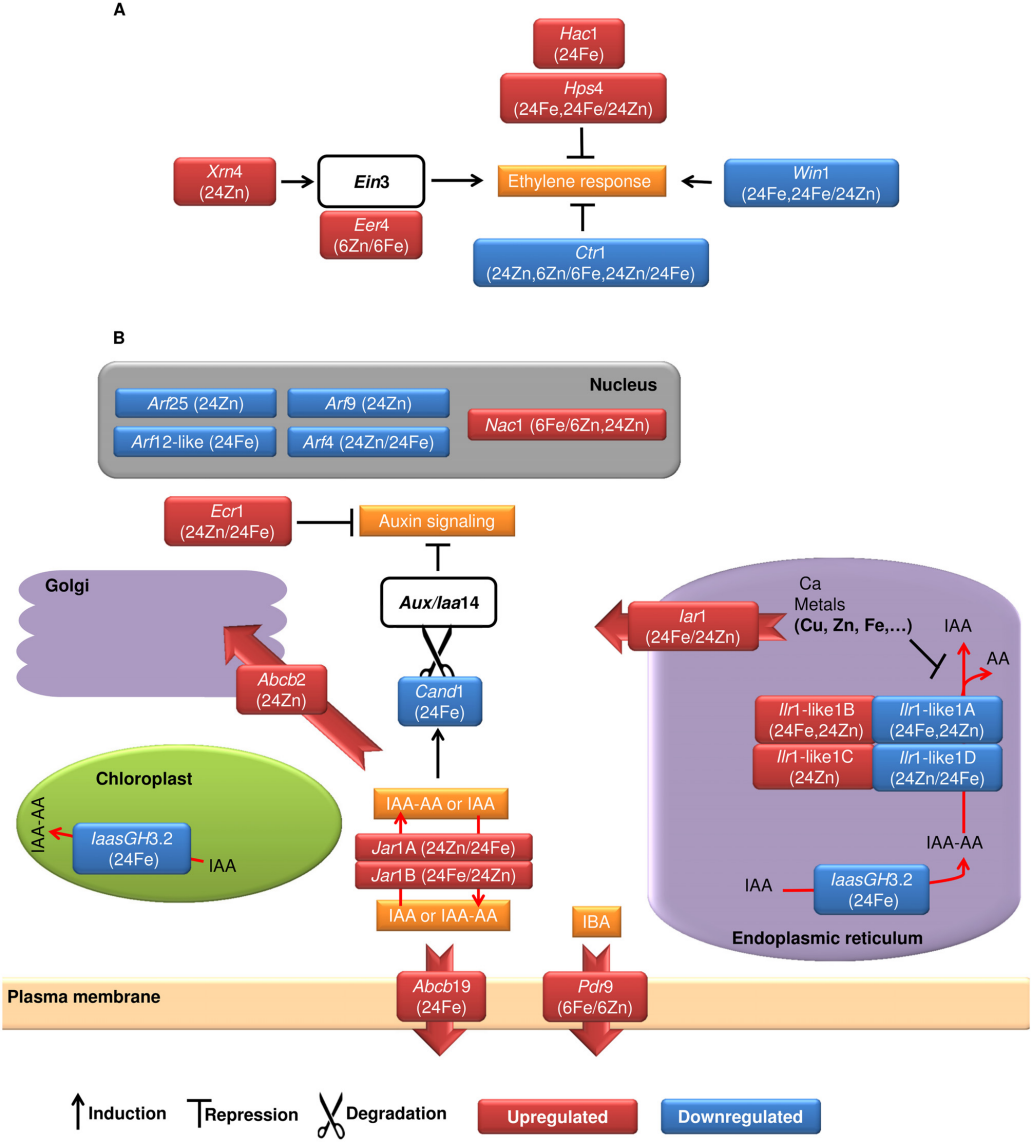
Hormonal signaling pathways. **(A)** Ethylene signaling. In contrast to iron-treated plants where the ethylene response was suppressed, zinc treatment triggered the signaling pathway. **(B)** Auxin (IAA) signaling. The treatments altered the cellular compartmentalization of auxin as well as its signaling pathways. The data reveals a very early-stage induction in auxin signaling after the treatments followed by negative feedback loops enabling auxin sequestration within organelles and cellular auxin efflux. This occurred likely due to iron surplus in the iron-treated plants as well as zinc repletion and/or continuous iron deficiency after foliar application of zinc. Accession numbers of the genes are available in S4 File. Different transcript isoforms of genes are shown by capital letters, immediately after the name of genes. Fe and Zn represent iron and zinc treatments. The samples of 6 h and 24 h after treatments are labeled by 6 and 24. UT stands for untreated plants. Comparisons of 24Fe/Untreated sample and 24Zn/Untreated sample are shown as 24Fe and 24Zn, respectively. Zinc treatment was compared with iron treatment which is shown as 6Zn/6Fe or 24Zn/24Fe. See S2 File or the text for detailed functions of genes. AA: amino acid, IBA: indole-3-butyric acid.

Our data addresses a key role for chromatin modifications in mineral homeostasis. The SKB1- mediated histone dimethylation interferes with iron uptake in *Arabidopsis* [18]. Accordingly, barley *Skb*1 showed higher expression in iron-treated plants compared to zinc-treated plants. Further, we found metal ion-triggered expression changes in 38 chromatin remodeling genes, providing genome-wide insights into the importance of fast chromatin-level responses to mineral availability. Comparing iron to zinc treatments, 27 of these genes were differentially expressed (S2 Table). Both regulatory and non-regulatory mineral homeostasis genes including *Ysl*, *Fro*2, *Nas*, *Hma*, *bHlH*39, *Irt*1, *Ferritin*, and many others follow light-dark cycles [2]. Most interesting, the histone methyltransferase *Sdg*2 which regulates the core clock components [19] was induced by iron and zinc (see S4 File for accession numbers). Disclosed by expression changes in the oscillator master-regulator genes (S2 Fig), SDG2 introduces a signaling pathway by which the metals can govern the plant circadian rhythm. As an ethylene-signaling negative regulator, the histone acetyltransferase *Hac*1 [20] was also induced by iron. Auxin and ethylene are key players in plant iron homeostasis mainly by working through the promotion of uptake mechanisms [2]. Our results revealed the suppression and enhancement of ethylene signaling by iron and zinc, respectively. After iron treatment, ethylene signaling was restricted by induction of known repressors; two hypersensitive to phosphate starvation genes *Hps*4 [21] and one serine/threonine-protein kinase gene *Ctr*1 [22] (Fig 4A). In contrast, the positive regulators *Win*1 and 5'-3' exoribonuclease *Xrn*4 [23] had higher levels of expression after zinc treatment (Fig 4A). We also found the EIN3-interacting transcription factor EER4 with higher expression in zinc-treated plants compared to iron-treated plants. The most likely scenario is the activation of iron deficiency response by the zinc repletion [see 2], underlined by the decreased iron content of seeds (Table 2). Therefore, iron-treated plants did not need rapid recovery from ethylene response which was consistent with the observed repression of EIN3-binding F-box gene *Ebf*1 that confers a fast feedback against ethylene [see 24].

**Table 2:** Seed mineral content.

Samples	Nutrients							
	Cu	Fe	Zn	Mg	Mn	P	S	K
**6Zn**	**3.2**	**20**	**23.4**	**794**	**9**	**2682**	**759**	**5684**
**24Zn**	**3.7**	**19.3**	**84.2**	**759**	**10.4**	**2574**	**839**	**5776**
**6Fe**	**3.5**	**67.8**	**21.7**	**594**	**5**	**2542**	**706**	**5854**
**24Fe**	**3.7**	**67.7**	**23**	**790**	**8.2**	**2524**	**759**	**5032**
**UT**	**3.9**	**22.1**	**25.3**	**852**	**9.3**	**2762**	**812**	**6026**

Total seed mineral contents (μg/g) of the samples were determined by ICP-MS. The quantities represent the average of four technical replicates with a coefficient variation of 1–2%. 6Fe: six hours after iron treatment, 6Zn: six hours after zinc treatment, 24Fe: 24 hours after iron treatment, 24Zn: 24 hours after zinc treatment, UT: untreated sample.

Recalling the importance of auxin in mineral homeostasis [2], its signaling cascade has yet to be established. Here, we introduce different components of auxin signaling involved in mineral homeostasis (Fig 4B). In general, the elevated iron and zinc levels were in accordance with suppression of auxin signaling (Table 2 and Fig 4B). ABCB/MDR transporters facilitate the sequestration within cellular compartments or efflux across the plasma membrane (PM) of auxin [25–27]. As auxin transporters, we found a putative Golgi ABCB2 as well as the PM ABCB19 induced by the treatments (Fig 4B). Resembling zinc-mediated iron deficiency signaling, the auxin precursor exporter *Abcg*37 (*Pdr*9) [26] had higher expression level in iron-treated plants compared to zinc after 6 h. It seems, the cells activated auxin signaling soon after the treatments followed by auxin sequestration within cellular compartments or efflux across PM. In parallel with the increased auxin efflux from cytosol, *Cand*1 which is involved in AUX degradation [28] was repressed by iron. AUX proteins are short-lived repressors of early auxin-response [29]. The auxin signaling repressor ECR1 [30] was also found to work in parallel after zinc treatment (Fig 4B). Accordingly, the auxin-response factors *Arf12*-like, *Arf*4, *Arf*25, and *Arf*9 were all repressed. The mutant *arf*12/25 plants show lower cellular iron, zinc, and auxin contents [31].

In addition to the auxin efflux and distribution, the reversible catalysis of less active auxin-amino acid (IAA-AA) conjugates [32] participates in mineral homeostasis. The influx of metals like zinc into the ER has been proposed to inhibit the IAA-leucine resistance 1 (ILR1)-mediated IAA-AA hydrolysis which can be activated by IAR1-facilitated metal efflux [32–35]. The expressional adjustments of a putative ER ZIP protein coding gene *Iar*1 likely released metals copper and zinc for vital biological processes in iron-treated cells and suppressed IAA-AA hydrolysis after zinc treatment (Fig 4B). A putative cytoplasmic IAA-amido synthetase GH3.5 (*Jar*1) switched transcript isoforms coding for an identical amino acid sequence when comparing iron and zinc treatments. Also, two probable chloroplast- and ER-localized IAA-amido synthetase *IaasGH*3.2 coding genes [36] were repressed which underlines decreased auxin levels in iron-treated plants.

Iron deficiency promotes the formation of branched root hairs [37, 38] and lateral root elongation is triggered after iron supply [39]. In this context, we noticed root growth and development factors *Nac*1 [40], the fusion gene nodulin/glutamine synthase [41], the auxin-induced in root cultures *Air*12 [42], and the root hair defective *Rhd*3 [43]. Their expression changes imply the enhancement of iron uptake/deficiency signaling and root elongation mechanism at early and later stages after iron treatment, respectively. Most likely, these genes are upstream factors responding to mineral fluctuations in different tissues.

### Excess zinc and iron trigger stress-response factors and affect photosynthesis and respiration

More than 20% of the responding genes were stress related, including DNA damage/repair, programmed cell death and autophagy, retroelements, and members of storage proteins. Different upstream stress response genes were employed to decrease ROS production and ROS-mediated cellular damage (S3 Fig and S4 Table, see S1 Text for functional details). The induction of 2Fe-2S binding domain-free coding transcripts in two xanthine dehydrogenase genes may be an adaptive strategy against iron deficiency after zinc treatment. These isoforms should also be resistant to conversion from the dehydrogenase to the oxidase form which produces superoxide and hydrogen peroxide [see 44].

**Fig 5:**
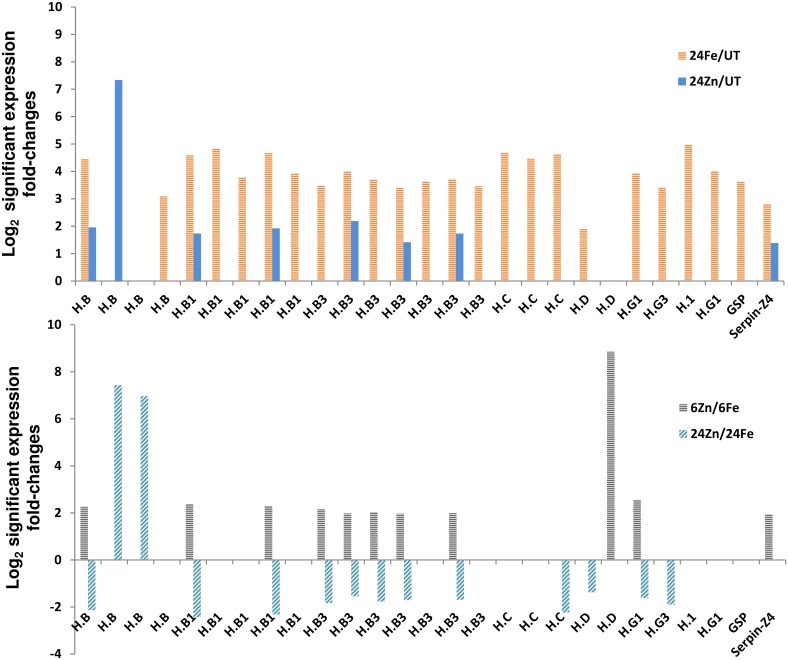
Differential expression of seed storage proteins. Genes encoding seed storage proteins including B, C, D, and G hordeins (H), were upregulated by the treatments. See S2 File for the accession numbers and gene names. 6Fe: 6 h after iron treatment, 6Zn: 6 h after zinc treatment, 24Fe: 24 h after iron treatment, 24Zn: 24 h after zinc treatment, UT: untreated plants. For example, 24Fe/UT represents the comparison of 24Fe with UT.

**Fig 6:**
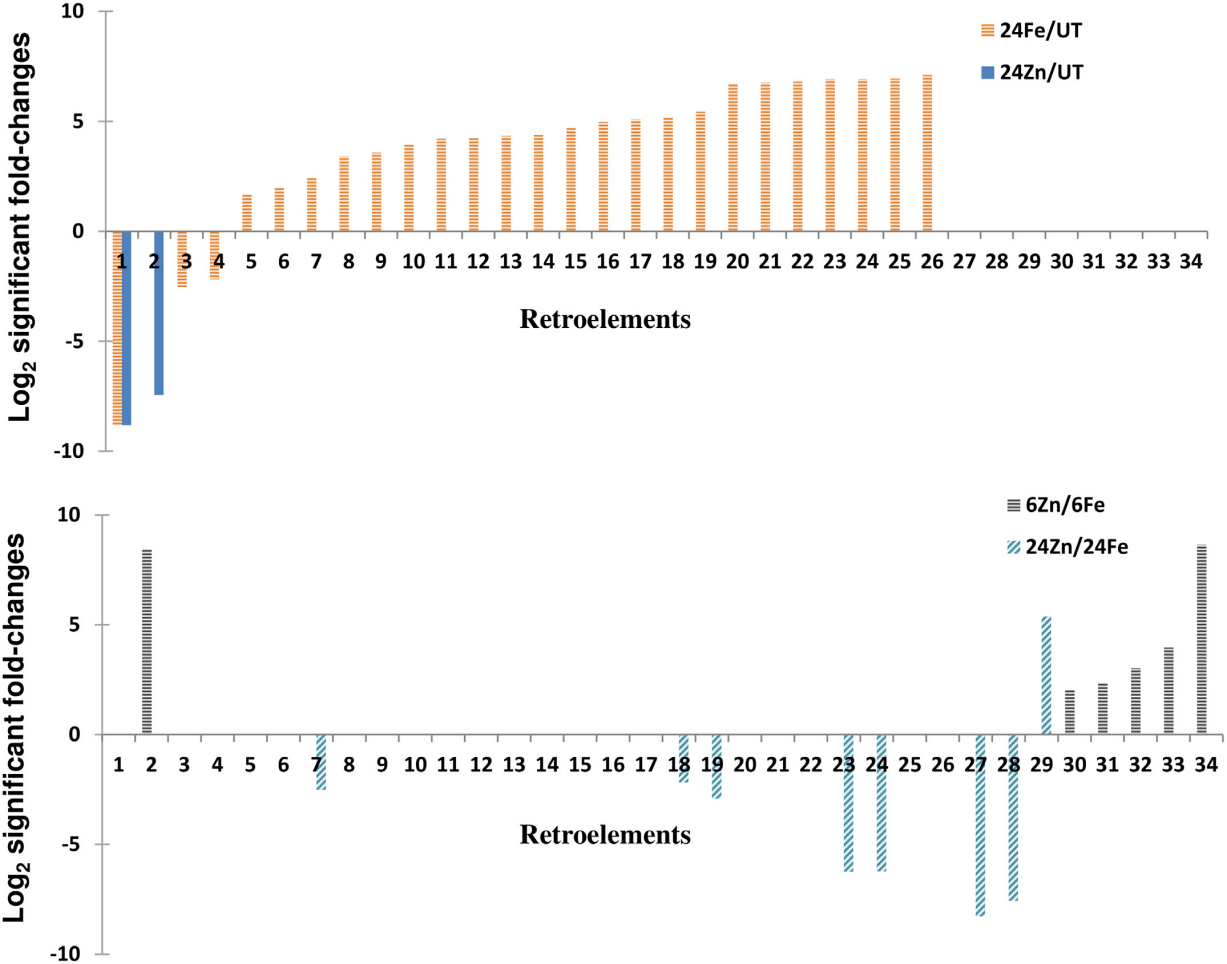
Retroelements were activated by foliar applications of iron and zinc. See S3 Table for the accession numbers and other details of events shown by numbers of 1–34. 6Fe: 6 h after iron treatment, 6Zn: 6 h after zinc treatment, 24Fe: 24 h after iron treatment, 24Zn: 24 h after zinc treatment, UT: untreated plants. For example, 24Fe/UT represents the comparison of 24Fe with UT.

Surprisingly, we found seed storage protein encoding genes induced by the treatments (Fig 5). Compared to the average amino acid composition of plant proteins, we noticed 750%, 350%, and 50% higher glutamine, proline, and cysteine frequencies in the upregulated storage proteins analyzed by the CLC software. Hydrolysis of hordeins and partial deamidation of glutamine residues leads to small peptides carrying ionized carboxyl groups with radical scavenging and metal chelation activities [45]. Therefore, we hypothesize a function for the induced storage proteins in both metal chelation and metal toxicity resistance. Considering the function of retroelements in plant adaptation to environmental cues like metal depletion [46, 47], we also noticed a fast activation of retroelements in response to metal surplus (Fig 6).

Our results also emphasize that photosynthesis and respiration were affected by zinc-mediated iron deficiency (S2 Text and S5 Fig). The data provides additional evidence to the interplay between iron and oxygen during the evolution as discussed in [2] by indicating a limited aerobic metabolism, i.e., suppression of Krebs cycle and electron transfer chains (S2 Text), to ensure less oxidative stress under excess iron levels. Taken together, the described negative side-effects of excess iron and zinc have implications for crop biofortification. Not only is it costly for the cells and potentially imposes a yield penalty, but the elevated cellular metal contents also trigger negative feedbacks, which work against the desired mineral levels and prioritize a minimum buildup of oxidative stress in biofortified crops.

### The S-adenosylmethionine cycle and mineral homeostasis

**Fig 7:**
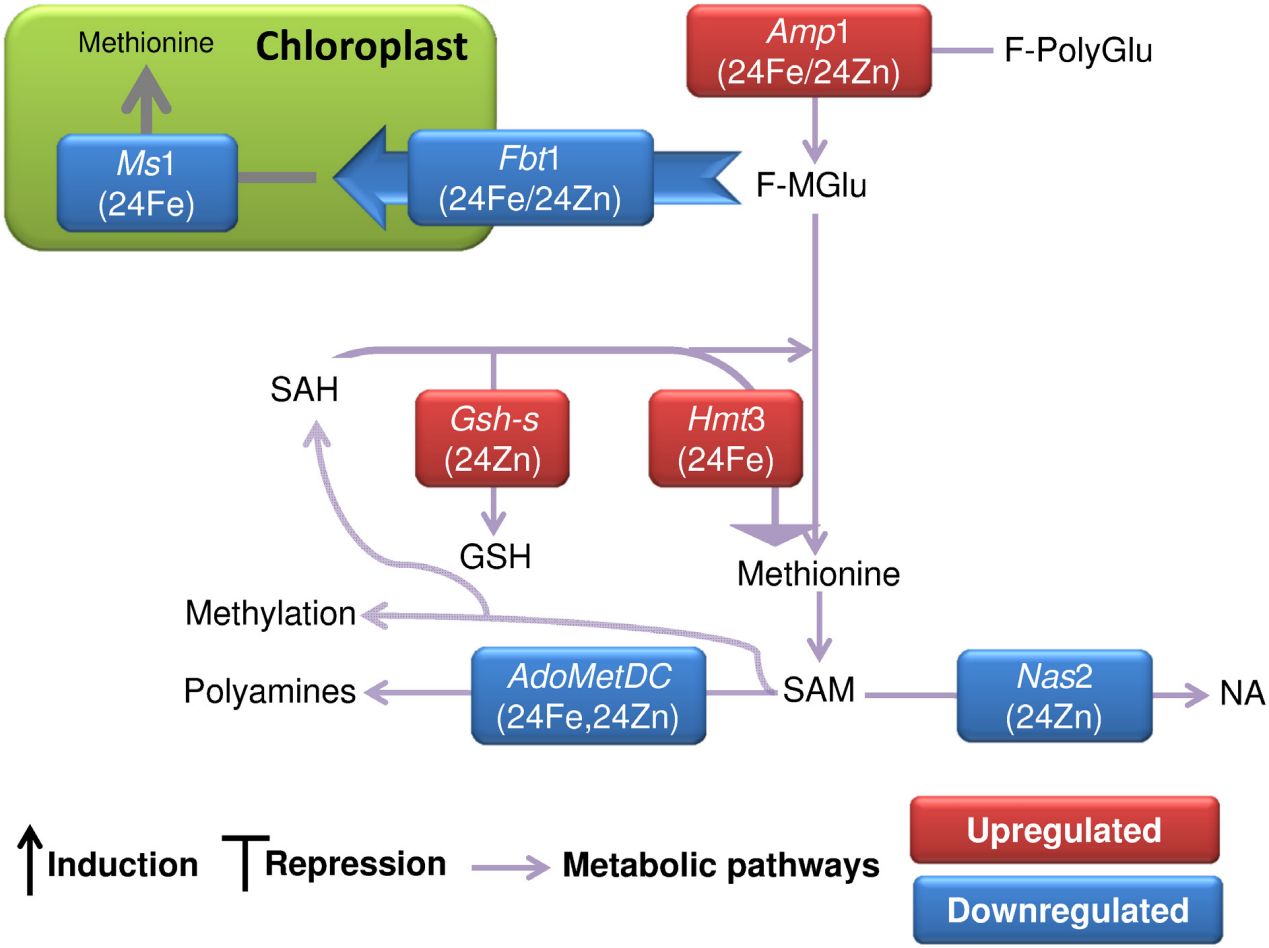
SAM plays an important role in mineral homeostasis. SAM biosynthesis was enhanced by iron. HMT catalyzes SAM biosynthesis by methyl transfer to homocysteine [53, 54]. The suppression of PA biosynthesis could results in a large pool of substrate “SAM” in favor of NA biosynthesis after iron treatment. Not only PA biosynthesis but also NA biosynthesis was suppressed by zinc. Concurrently, glutathione synthetase (*Gsh-s*) was induced. Accession numbers of the genes are available in S4 File. Fe and Zn represent iron and zinc treatments. The samples of 6 h and 24 h after treatments are represented by 6 and 24. UT stands for untreated plants. Comparisons of 24Fe/Untreated sample and 24Zn/Untreated sample are shown as 24Fe and 24Zn, respectively. Zinc treatment was compared with iron treatment which is shown as 6Zn/6Fe or 24Zn/24Fe. HMT: homocysteine S-methyltransferase, F-MGlu: folate monoglutamate, F-PolyGlu: folate polyglutamate, NA: nicotianamine, PA: polyamine, SAH: S-adenosylhomocysteine, SAM: S-adenosylmethionine.

Nicotianamine is a key player in metal transport and chelation [48]. Most upstream in the nicotianamine biosynthetic pathway, glutamate carboxypeptidase 2 (AMP1) releases folate monoglutamate from folate polyglutamate [49]. The *amp*1 mutants are insensitive to nitric oxide [50]. Therefore, repression of *Amp*1 can be explained as a negative feedback against nitric oxide-triggered iron uptake under continuous iron deficiency after 24 h of zinc treatment (Table 2 and Fig 7). In contrast, iron treatment enhanced the transcription of *Amp*1 to promote S-adenosylmethionine (SAM) biosynthesis. The repression of chloroplast folate-biopterin transporter *Fbt*1 [51] and methionine synthase *Ms*1 [52] and the induction of homocysteine S-methyltransferase *Hmt*3 [53, 54] could also trigger the production of nicothianamine precursor “SAM” (Fig 7). The gene coding for SAM decarboxylase (AdoMetDC), which utilizes SAM for biosynthesis of polyamines [55], was also repressed by iron. Therefore, SAM was preferably channeled to the nicotianamine biosynthesis after iron treatment. In contrast, zinc treatment repressed the nicothianamine and polyamine biosynthesis pathways in favor of glutathione prouction (Fig 7).

Ethylene-insensitive3 like 3 (EIL3) may co-regulate cellular metal and SAM reserves. EIL3 is involved in high-affinity sulfate uptake [54] and can be induced by iron deficiency [56]. *Eil*3 accordingly showed higher expression in iron-treated plants compared to zinc-treated plants after 6 h, perhaps to meet the lower seed sulfur content of iron-treated plants (Table 2). This was followed by the enhanced SAM production in iron-treated plants after 24 h. We also found the transcription factor *Vip*1 repressed in the zinc-treated plants. *Arabidopsis* plants defective in VIP1 have higher contents of sulfur, glutathione and cysteine [57]. Accordingly, the seed sulfur content was elevated by the zinc treatment (Table 2).

### Alterations in metal-trafficking components

**Fig 8:**
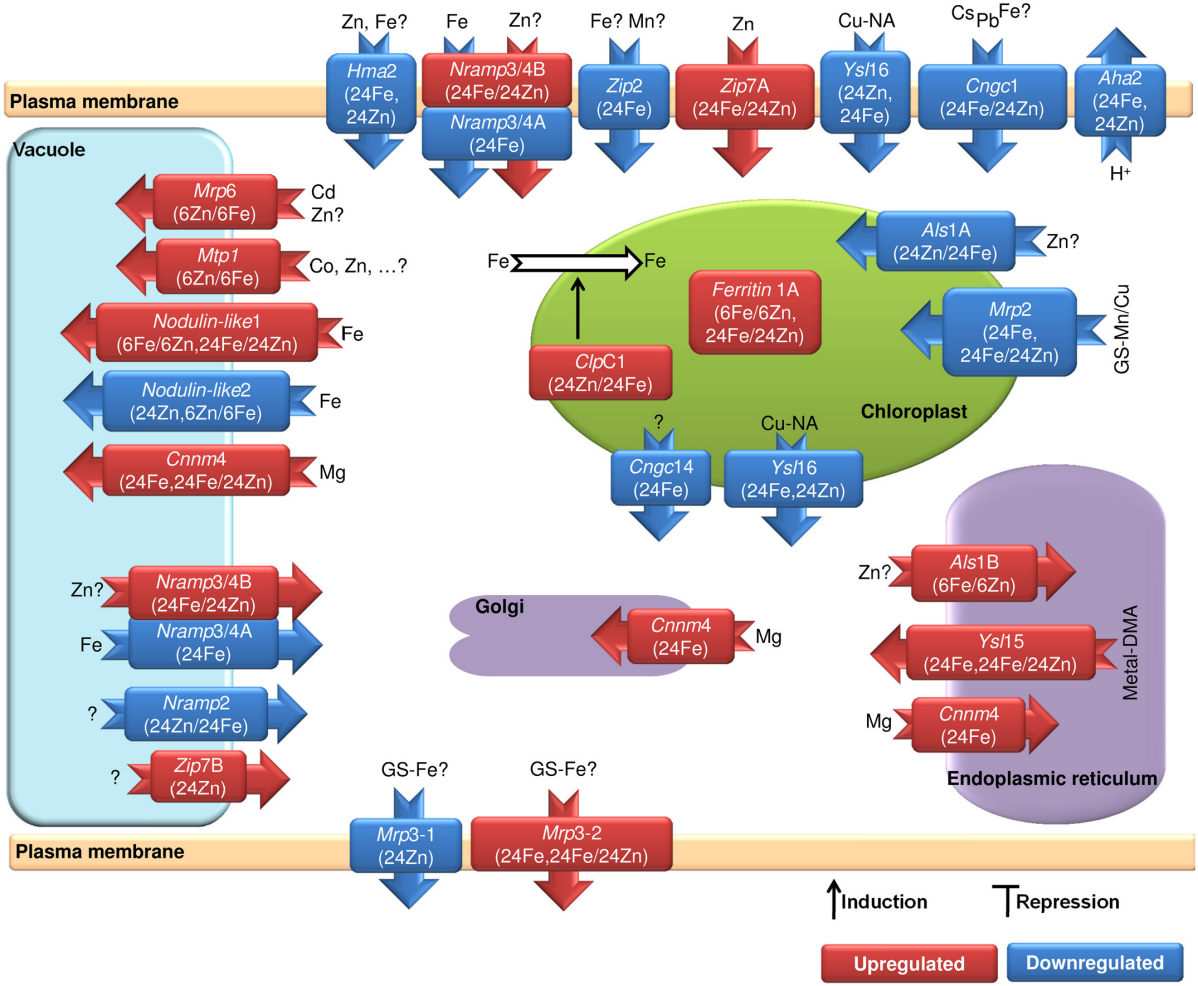
Expression changes in metal transporters. The treatments compelled the cells to restrain the iron or zinc uptake routes across the plasma membrane and chloroplast envelope and to sequester the minerals within vacuoles. In parallel, uptake and internal-source utilization of other metals were enhanced. This indicates a strong dependency of cellular levels of minerals on each other and sheds light on the negative feedback loops to avoid excess mineral contents. Accession numbers of the genes are available in S4 File. Different transcript isoforms of genes are shown by capital letters, immediately after the name of genes. Fe and Zn represent iron and zinc treatments. The samples of 6 h and 24 h after treatments are represented by 6 and 24. UT stands for untreated plants. Comparisons of 24Fe/Untreated sample and 24Zn/Untreated sample are shown as 24Fe and 24Zn, respectively. Zinc treatment was compared with iron treatment which is shown as 6Zn/6Fe or 24Zn/24Fe. DMA: deoxymugineic acid, GS: glutathione, NA: nicotianamine.

Members belonging to a diverse set of metal transporter families were in action against the excess mineral supply (Fig 8). The treatments compelled the cells to restrain their uptake routes of iron or zinc. Vacuolar sequestration was also activated. Concurrently, uptake and internal-source utilization of other metals were enhanced. Indicating the plasticity of plant mineral homeostasis in terms of regulation and negative feedbacks to the metal repletion, this sheds light on the potential usage of upstream regulatory components for crop biofortification strategies.

The *Arabidopsis* proton pumps, *Aha*2 is working in iron uptake from the rhizosphere [58]. Here, we report the barley *Aha*2 gene repressed in the transfer cells by both zinc and iron treatments (Fig 8). This introduces the apoplastic alkalinization as a widespread mechanism in plants which serves as the first barrier to lessen the uptake under metal repletion. As negative feedback loops, furthermore, a coordinated action of metal uptake and storage mechanisms were opposed to the enhanced cellular metal contents. HMA transporters are elaborated zinc and copper transporters [59–63]. The PM *Hma*2 was repressed by zinc and iron which supports the rice HMA2 influx activity reported in [64]. It needs further evidence on whether HMA2 can transport iron or the iron-mediated suppression was an inevitable regulatory response. We also found the vacuolar zinc transporter of Cation Diffusion Facilitator *Mtp*1 [65] with higher expression in plants treated by zinc compared to iron after 6 h (Fig 8).

We noticed different members of the ABC transporters influenced by the treatments. In *Arabidopsis*, *Mrp*3 can be induced by cadmium, nickel, arsenic, cobalt and lead but not by zinc and iron [66]. Most interestingly, our transcript-level comparisons revealed the contribution of *Abcc*3 (*Mrp*3) to the iron homeostasis (Fig 8). The higher expression of *Abcc*8-like in zinc-treated plants compared to iron-treated plants was likely to sequester zinc within vacuoles. Its homologue in *Arabidopsis*, MRP6, is involved in cadmium detoxification [67]. Recalling the glutathione S-Mn/Cu conjugate transport activity of *Arabidopsis* ABCC1 and ABCC2 [68, 69], the expression changes of *Abcc*2 (*Mrp*2, Fig 8) explain the additional supply of manganese and copper to recover the distressed photosynthesis (consult S2 Text and Table 2). Furthermore, the protease CLPC1 which is a key factor to supply chloroplasts with iron [70] had higher expression after zinc treatment compared to iron (Fig 8). Finally, the repression of *Abcb*27 as the homologue of aluminum sensitive ALS1 in rice [71], was conceivably to hinder zinc-overloading of chloroplasts (Fig 8).

As a subgroup of the oligopeptide transporter family (OPT), YSL proteins are metal transporters [72–76]. We found a putative ER/vacuolar YSL15 coding gene induced by iron. Its homologue in rice, OsYSL15, transports Fe (III)-deoxymugineic acid and displays inward transport in yeast experiments [74, 75] making vacuolar sequestration of iron unlikely. It seems HvYSL15 releases metals out of the ER in parallel with *Iar*1 (Figs 4B and 8). The Chloroplast/PM YSL16 coding gene was repressed by both zinc and iron. Rice YSL16 imports Cu-nicotinamine complex across PM [77]. Therefore, this response is likely to preserve the chloroplast copper. The grain copper content was decreased by the treatments (Table 2).

The *Arabidopsis* NRAMP3 and 4 export iron and manganese out of the vacuoles [78, 79]. We found a putative PM/tonoplast NRAMP3/4-like isoform repressed by iron treatment. Another transcript isoform of this gene had higher expression level in iron-treated plants compared to zinc. While *Thlaspi caerulescens* NRAMP4 transports iron and zinc, TcNRAMP3 only transports iron [80]. In contrast, AtNRAMP2 does not transport iron [81]. Accordingly, a putative tonoplast NRAMP2 displayed lower expression by zinc compared to iron. This gene likely releases the vacuolar zinc. As we noticed after zinc application, iron deficiency downregulates the vacuolar iron importer nodulin-like 2 in *Arabidopsis* [82]. The nodulin-like 1 had accordingly lower expression in zinc-treated plants compared to iron (Fig 8). Furthermore, a putative PM zinc transporter 2 (*Zip*2) was repressed by iron (Fig 8). Recalling the zinc and manganese transport activities of AtZIP2 [83, 84], the response is supported by the reduced manganese levels after iron treatment (Table 2). It seems HvZIP2 is not very specific and translocates other metals like iron as well. Additionally, the barley PM ZIP7 [85] had higher expression in iron-treated plants compared to zinc after 24 h. As for *Nramp*2, we noticed alternative transcripts of *Zip*7, encoding for small-size proteins. Whether these microproteins contribute to the transport activity or act in negative regulation of NRAMP2 and ZIP7 need further experiments.

Among the toxic metal transporters of cyclic nucleotide-gated channels [86–88], the *Cngc*1 and *Cngc*14 were differentially expressed. Furthermore, two dentin sialophosphoprotein-like genes had higher expression levels in iron-treated plants compared to zinc after 6 h. In human, the distribution of dentin sialophosphoprotein regulates mineral deposition and when mutated, it resembles the abnormal tooth biomineralization by defective cyclin M4 (*cnnm*4) [89]. The PM ancient conserved domain protein CNNM4 has magnesium efflux activity in mice [90]. Accordingly, iron induced putative tonoplast and Golgi/ER CNNM4 proteins with CBS domain (Fig 8). The CBS domain is known from bacterial CorC and CorB magnesium/cobalt efflux transporters [91, 92]. The suppression of vacuolar magnesium storage in zinc-treated plants after 24 h was in agreement with the repression of *Cbl3*. The tonoplast CBL3-CIPK23 calcium signaling promotes vacuolar magnesium sequestration in *Arabidopsis* [93]. This emphasizes that the vacuolar-function is central in the metal and calcium cross homeostasis proposed in [2]. Shown in Fig 8, vacuoles are playing crucial role in mineral homeostasis by storing and releasing of micronutrients in repleted and depleted cells, respectively. Accordingly, the membrane protein YMR155W-like coding gene was downregulated by iron. When mutated, it affects vacuolar membrane fragmentation and shows metal resistance phenotype in yeast [94, 95]. Therefore, the repression of *Ymr*155W likely enhances metal sequestration through vacuolar organization. We also found the vacuolar protein sorting 4 with higher expression in iron-treated plants compared to zinc. *Arabidopsis* VPS4 is involved in the protein trafficking and maintenance of large central vacuole [96]. The same expression pattern was found for the gene *Rab*G3F that together with monensin sensitivity 1(*Mon*1) is involved in prevacuolar compartments-to-vacuole trafficking and vacuole biogenesis [97]. MON1 is also involved in iron homeostasis of animal cells [98, 99].

Human RNA poly(rC)-binding proteins (PCBPs), as iron chaperones, are involved in delivering iron to non-heme iron containing proteins [100, 101]. Here, we report expression changes in PCBPs and thereby their potential role in plant mineral homeostasis not only by iron chaperoning but also through post-transcriptional regulation [see 102]. Recently, the mediator subunit 16 has been identified as a very upstream positive regulator of iron uptake in *Arabidopsis* [103]. Here, we report a sharp repression for *Med*16 after iron treatment implying a common regulator of iron homeostasis in graminaceous and non-graminaceous plants. Additionally, an iron-induced hemerythrin-like gene possibly worked as the repressor of iron uptake factors. The hemerythrin-like FBXL5 senses iron and oxygen availability and thereby negatively regulates iron absorption in mammalian cells [104–106]. Finally, we noticed the serine/threonine-protein kinase TOR and replication protein RPA which recently have been introduced as iron homeostasis components [107]. *Rpa*2, a homologue of *GmRpa*2A, was repressed after both of the treatments. Two other *Rp*a2 genes were induced by zinc. Taken together, the adaptability of cellular mineral homeostasis to avoid excess metal levels highlights the advantage of regulatory factors over the downstream transporters in crop biofortification.

### Intracellular protein-sorting regulates metal homeostasis

Our analysis also showed the importance of endocytosis based regulation in mineral homeostasis. Auxilin-related protein 2 which disturbs endocytosis and aluminum loading through uncoating of clathrin-coated vesicles in *Arabidopsis* [108], showed higher expression by iron compared to zinc, and likely halts iron uptake. The myosin-Vb gene had similar expression pattern. Together with RAB11A, animal myosin Vb retards recycling of the transferrin receptor to PM and affects iron import [109]. Responsible for endocytosis of animal transferrin receptor [110], the epidermal growth factor receptor substrate 15-like was properly repressed by iron. Conceivably, a similar mechanism functions in planta against and in favor of iron uptake after 24 h of iron and zinc treatments, respectively. In addition to the degradation-by-internalization of iron transporter IRT1 [111], the internalization of rice manganese transporter NRAMP3 has been reported under high manganese concentrations [112]. Accordingly, two protein transport *Sec*23A-like genes were induced by the treatments. The zinc-induced *Sec*23A had lower expression in zinc-treated samples compared to iron after 6 h. Yeast SEC23A is involved in internalization of PM proteins [113]. Therefore, plant SEC23A proteins are good candidates for disassembling of PM metal transporters. Here, we also highlight the metallophosphoesterase gene MPP_Cdc1_like which positively regulates manganese uptake in yeast [114]. It was upregulated after 24 h of zinc treatment. This is in accordance with the increased manganese content seen after 24 h of zinc application (Table 2). Compared to the zinc-treated plants, two other transcripts of MPP_Cdc1_like had higher expression levels in iron-treated plants after 6 h. Considering both the yeast *cdc*1 mutant complementation by mutations defective in vacuolar sorting of ubiquitinated proteins [114] and the CDC1 facilitated ER-to-PM/cell wall protein transport in yeast and human [115, 116], we propose that the gene MPP_Cdc1_like triggers the docking/integration of PM metal transporters like NRAMP3. We also highlight the homologue of At*Exo*70E1 (*Exoc*7) which was induced by iron. It may facilitate polar accumulation of transporters in the PM [117]. In *Arabidopsis*, overexpression of the FYVE1 accumulates PM IRT1 in apolar mode impairing the rate of metal uptake [118]. Phosphatidylinositol-3 and 4-phosphate are also involved in intracellular polarized and apolar trafficking of metabolites and proteins [119]. Not only the phosphatidylinositol-3-phosphate binding WD repeat and FYVE domain-containing protein 3 was induced in zinc-treated plants but also the induction of genes involved in the phosphatidylinositol-3-phosphate biosynthesis and recycling was observed (S5 Table). Moreover, the genes responsible for its usage were repressed. Therefore, higher phosphatidylinositol-3-phosphate seems to be a key trafficking factor under zinc repletion.

## Conclusions

Cellular shortage and surplus of metal are both hindering cellular metabolism due to the diverse structural/catalytic roles and the enhancement of oxidative stress, respectively. We observed a high degree of complexity in iron and zinc homeostasis which seems to be the aftermath of the engagement of metals at a vast numbers of metabolic pathways. Therefore, genetic engineering for sustainably improved crop fitness against micronutrient depletion and repletion are laborious goals. The cellular impact of excess micronutrients including changes in hormonal and circadian clock signaling, affected photosynthesis and respiration, negative feedback loops and triggered oxidative stress are not trivial in terms of affecting the plant fitness. Tissue specific genetic manipulation can leave out some of the complexities. However, this can suffer from the weak tissue-specific gene promoters which we personally have realized by independent transgenic experiments conducted in our laboratory. Moreover, all the regulatory mechanisms acting on the genes, transcripts, and proteins of interest should be taken into account in the efforts to bypass the negative feedback loops. Here, we highlight the docking and recycling mechanisms and uneven distribution of mineral transporters in plasma membrane. Overexpression of influx and efflux metal-transporters in a polar localized fashion can be very efficient to enhance the directional flow of minerals. Specific targeting of the upstream regulatory genes can be another strategy. However, all the scenarios demand a high resolution map of metal homeostasis. Here, our attempt furnishes the current knowledge with the vast reaches of transcriptomic maps in iron and zinc homeostasis of barley transfer cells. This report not only covers the known grass and non-graminaceous mineral homeostasis components, but also introduces new interactors into the plant mineral homeostasis. This includes different adaptation mechanisms plant cells use against alterations in mineral availability.

## Materials and Methods

### Experimental design

Field-grown barley (*Quench cv*.) plants at the growth stage 18±2 days after anthesis were treated by foliar application of 15 mM FeSO_4_.7H_2_O or 0.5% of ZnSO_4_.7H_2_O. Representing the control (UT) and 24 h after treatments (24Fe and 24Zn), immature seeds were collected from untreated and treated plants simultaneously at 8:30 a.m. Samples of 6 h after treatments (6Fe and 6Zn) were collected the day before at 2:30 p.m. We did not compare 6 h after treatments with either of untreated plants or samples collected 24 h after the treatments due to the possibility of interaction between mineral and diurnal-based changes. All four preferred comparisons (24Fe/UT, 24Zn/UT, 24Zn/24Fe, and 6Zn/6UT) were performed on transcript data as well as gene quantities. Three spikes from individual plants were collected representing three biological replicates. Samples were stored at -80°C before RNA extraction. Pooled RNAs extracted from three seeds representing top, middle, and bottom part of each spike was applied as one biological replicate. Finally, a single Illumina sequencing run including three channels each containing one of the three replicates from all the five samples was applied.

### Total RNA extraction and sequencing

**Fig 9:**
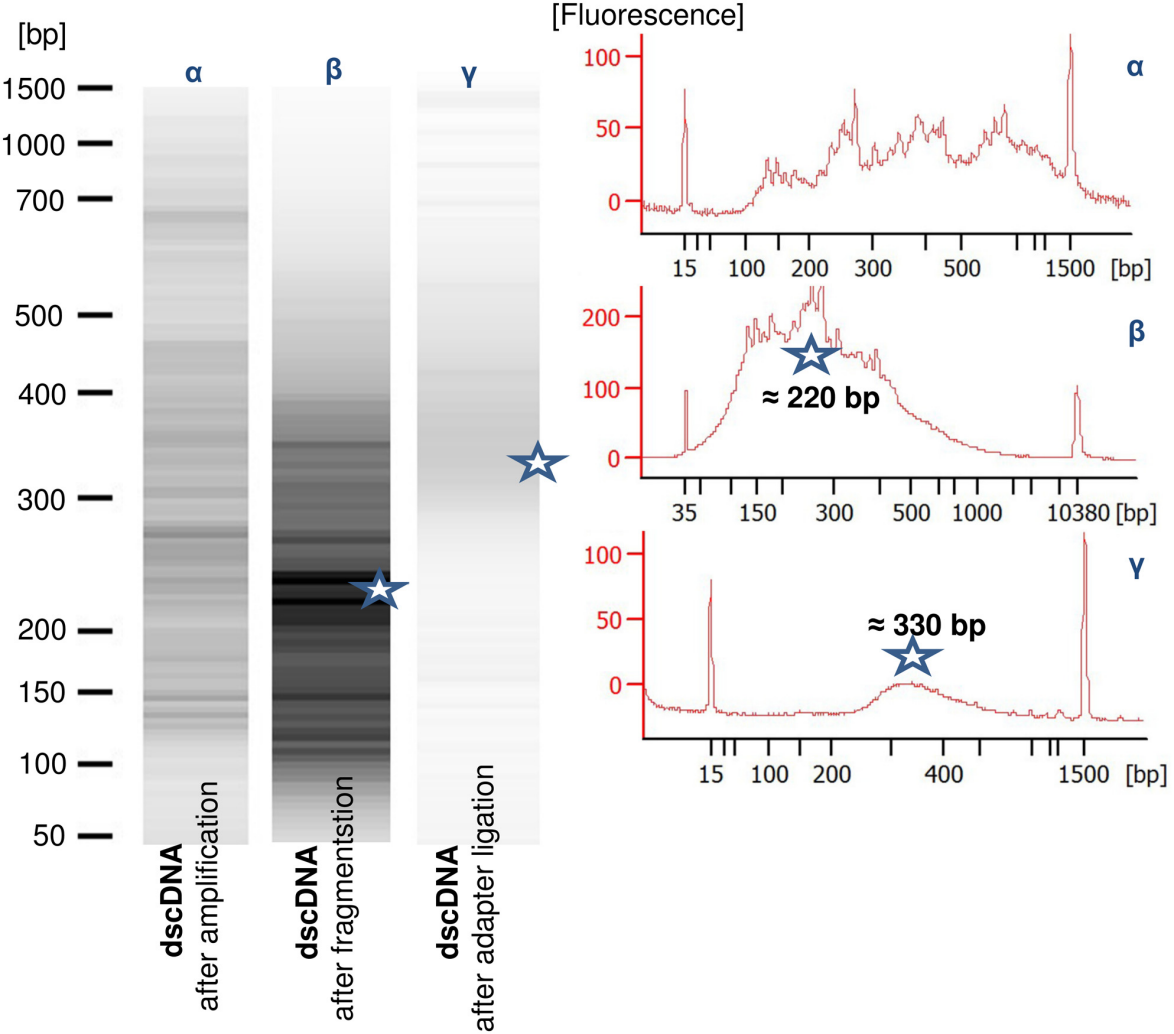
Library preparation for sequencing. Double-stranded cDNA profile after *in-vitro* linear amplification, fragmentation, and adaptor ligation is shown. Fragmentation enriched samples for ≈ 220 base-long fragments. Ligation of adaptors at both ends of the fragments changed the average length to ≈ 330 base pairs.

Laser capture microdissection [15] was used to collect transfer cells from 70 μm grain cryosections. Twenty sections from each seed and 60 in total were laser-bombarded for each of the biological replicates. Arcturus PicoPure RNA isolation kit (cat no. 12204–01) was used to isolate total RNA. RNA quantity and quality was determined by Agilent 2100 Bioanalyzer. For Real-Time PCR, 4 μl of the RNA samples were kept and the rest (≈ 28 μl) were used in sequencing. Due to the low quantity of samples ranging from 3.6 to 18 ng (S7 Table), linear amplification of RNAs was performed using NuGEN Ovation^®^ RNA-Seq kit (Fig 9). In parallel with the amplification, the semi-random primers of the kit also enriched the RNA population of samples against ribosomal RNA. Paired end 2 × 101 bp sequencing was performed at AROS Applied Biotechnology A/S (Aarhus, Denmark) using HiSeq 2000 Illumina sequencing platform and Truseq technology. CASAVA 1.8.2 was used to convert sequencing results into fastq format. The data are available under the GenBank accession number of SRA297575. The ABI Applied Biosystems 7900 HT Thermal Cycler used for Real-Time PCR (see S5 File for primers). The random-primed first-strand cDNAs were synthesized using Superscript II (Invitrogen Life Technologies, Carlsbad, CA, USA) according to the manufacturer’s instructions.

### RNA-Seq data analysis

The quality of the sequencing reads were checked by FastQC version 0.10.1. Based on the overall high quality of the sequencing reads (S6A Fig), we did no trimming except the TopHat automatic filtering. Unmapped reads were then trimmed using Trimmomatic-0.30 [120]. The 5ʹ and 3ʹ end of reads were hotspots of Kmers and imbalanced nucleotide content (S6B and S6C Fig). Therefore, we trimmed for the first 30 and the last 11 nucleotides of the reads. In addition, a quality filtering was applied for both ends of the reads with nucleotides showing lower than 23 quality score in Phred-33 scoring system. Additionally, we performed a sliding trimming with a window size of four and average quality score of 29. The minimum acceptable read-length was 30 bases. The trimmed reads were mapped and unmapped reads were further trimmed for the 31–35^th^ and 80–90^th^ nucleotide positions of original reads. Second-round trimmed reads were mapped and the remaining unmapped reads were excluded. Bowtie2-2.1.0 [121] was used to index the barley genome release 18 (*EnsemblPlants*: http://plants.ensembl.org/info/data/ftp/index.html). Tophat-2.0.8b performed a gapped alignment to predict exon-exon junctions and detect alternative spliced isoforms [122]. To map the original reads, the inner distance between pairs was defined to 40 bases with standard deviation of 141 bases. These could cover from a complete match to 181 bases distance between pairs. The mean length of the sequenced fragments was ≈ 300–330 with inner distance of 100–120 bases between pairs (Fig 9). We allowed three and two mismatches per original and trimmed reads and defined the minimum and maximum intron length of 35 and 80000 bases, respectively. Single-hit-report option was also activated. To enrich the gene model annotations of barley genome release 18, the detected splice-sites after each mapping step were used for the next mapping. After the three rounds of mapping, the cuffmerge utility of Cufflinks-2.1.1 was used to include all the known and identified novel transcripts in the analysis by building a new genome annotation file [see 123]. The Tophat outputs for the three rounds of mapping were merged for each sample-replicate using Samtools-0.1.19 [124]. Finally, the merged new annotation file and merged accepted hits were applied to count the expression quantities using SAMMate-2.7.4 [125].

### Statistical analysis

The inter-treatment comparisons were carried out using the edgeR [126]. Global dispersion was applied and the significant expression changes were selected with minimum two-fold changes using the corrected *p*-value cut-off of 0.01. Multiple testing correction was applied to correct the *p*-values [127]. Considering the outlier values in weak expression events, a low quantity filtering was performed before statistical analysis. Events were not excluded when the average quantity in each comparison (two samples) and the averages of three possible two-replicate combinations, at least in one of samples, were ≥ 5 reads per million.

### Functional annotation

We performed a manual functional annotation of novel transcripts and checked the previously annotated events. This was limited to the events which statistically showed significant changes in the comparisons. We extracted the functional information of genes from Ensemblplants (at http://plants.ensembl.org/index.html) and then, we carried out an extensive homology search for the novel and also annotated transcripts at both nucleotide and protein levels. We utilized different data bases including NCBI (http://blast.ncbi.nlm.nih.gov/Blast.cgi?CMD=Web&PAGE_TYPE=BlastHome), TAIR (http://www.arabidopsis.org/), Rice database (http://rice.plantbiology.msu.edu/), and UniProtKB/Swiss-Prot (http://www.uniprot.org/uniprot/). To assign a function, we followed the minimum similarities of 90% at the protein level and 80% at the nucleotide level, and an E value below 10^−30^. With the ambition to use the most recent functional studies, we did an extensive literature search to get a more complete picture of the function of genes. Subcellular localization of proteins was predicted by the PlantLoc server [128]. All the data are available in S2 File.

## Supporting Information

S1 Fig Real-Time PCR results. (PDF)

S2 Fig Differentially expressed components of circadian rhythm. (PDF)

S3 Fig Differentially expressed upstream regulatory genes involved in cellular stress response. (PDF)

S4 Fig Differentially expressed genes and transcripts involved in protein folding and degradation. (PDF)

S5 Fig Chloroplastic tetrapyrrole, heme, and chlorophyll biosynthetic pathways were influenced after the treatments. (PDF)

S6 Fig Quality check of sequencing reads. (PDF)

S1 File Selected reference genes for bias structure analysis. We analyzed 43 publicly-available microarray datasets including 891 different barley samples to find the most stable reference genes.
(PDF)

S2 File Differentially expressed events found in different comparisons. (XLSX)

S3 File Genes showing alternative isoform switching among transcripts which code for an identical amino acid sequence. (XLSX)

S4 File Accession numbers of the genes discussed in the main text. (PDF)

S5 File Real-time PCR primers. (PDF)

S1 Table Read mapping statistics. (PDF)

S2 Table Differentially expressed genes involved in chromatin remodeling. (PDF)

S3 Table Differentially activated retroelements. (XLSX)

S4 Table Differentially expressed genes involved in DNA-damage response. (PDF)

S5 Table Differentially expressed genes involved in phosphatidylinositol-3-phosphate biosynthesis, recycling, and usage. (XLSX)

S6 Table Differentially expressed genes positively involved in biotic stress response. (PDF)

S7 Table Quality check of RNA samples. (PDF)

S1 Text Iron- and zinc-triggered stress responses. It describes some of the stress-response genes found differentially expressed after the treatments.
(PDF)

S2 Text Excess zinc and iron affect photosynthesis and respiration. (PDF)

## References

[1] Sinclair SA, Krämer U. The zinc homeostasis network of land plants. Biochim Biophys Acta. 2012;1823: 1553–1567. 10.1016/j.bbamcr.2012.05.016 22626733

[2] Darbani B, Briat JF, Holm PB, Husted S, Noeparvar S, Borg S. Dissecting plant iron homeostasis under short and long-term iron fluctuations. Biotechnol Adv. 2013;31: 1292–1307. 10.1016/j.biotechadv.2013.05.003 23680191

[3] Cakmak I, Yilmaz A, Kalayci M, Ekiz H, Torun B, Erenoglu B, et al Zinc deficiency as a critical problem in wheat production in Central Anatolia. Plant and Soil 1996;180: 165–72.

[4] Abadía J, Vázquez S, Rellán-Álvarez R, El-Jendoubi H, Abadía A, Alvarez-Fernández A, et al Towards a knowledge-based correction of iron chlorosis. Plant Physiol Biochem. 2011;49: 471–482. 10.1016/j.plaphy.2011.01.026 21349731

[5] Loladze I. Hidden shift of the ionome of plants exposed to elevated CO2 depletes minerals at the base of human nutrition. Elife 2014;3: e02245 10.7554/eLife.02245 24867639 PMC4034684

[6] Becher M, Talke IN, Krall L, Krämer U. Cross-species microarray transcript profiling reveals high constitutive expression of metal homeostasis genes in shoots of the zinc hyperaccumulator *Arabidopsis halleri* . Plant J. 2004;37: 251–268. 14690509 10.1046/j.1365-313x.2003.01959.x

[7] van de Mortel JE, Almar Villanueva L, Schat H, Kwekkeboom J, Coughlan S, Moerland PD, et al Large expression differences in genes for iron and zinc homeostasis, stress response, and lignin biosynthesis distinguish roots of *Arabidopsis thaliana* and the related metal hyperaccumulator *Thlaspi caerulescens* . Plant Physiol. 2006;142: 1127–1147. 16998091 10.1104/pp.106.082073 PMC1630723

[8] Kobayashi T, Suzuki M, Inoue H, Itai RN, Takahashi M, Nakanishi H, et al Expression of iron-acquisition-related genes in iron-deficient rice is co-ordinately induced by partially conserved iron-deficiency-responsive elements. J Exp Bot. 2005;56: 1305–1316. 15781441 10.1093/jxb/eri131

[9] Narayanan NN, Vasconcelos MW, Grusak MA. Expression profiling of *Oryza sativa* metal homeostasis genes in different rice cultivars using a cDNA macroarray. Plant Physiol Biochem. 2007;45: 277–286. 17468002 10.1016/j.plaphy.2007.03.021

[10] Yang TJ, Lin WD, Schmidt W. Transcriptional profiling of the *Arabidopsis* iron deficiency response reveals conserved transition metal homeostasis networks. Plant Physiol. 2010;152: 2130–2141. 10.1104/pp.109.152728 20181752 PMC2850031

[11] Singh AK, McIntyre LM, Sherman LA. Microarray analysis of the genome-wide response to iron deficiency and iron reconstitution in the cyanobacterium *Synechocystis* sp. PCC 6803. Plant Physiol. 2003;132: 1825–1839. 12913140 10.1104/pp.103.024018 PMC181269

[12] Bernal M, Casero D, Singh V, Wilson GT, Grande A, Yang H, et al Transcriptome sequencing identifies SPL7-regulated copper acquisition genes FRO4/FRO5 and the copper dependence of iron homeostasis in *Arabidopsis* . Plant Cell. 2012;24: 738–761. 10.1105/tpc.111.090431 22374396 PMC3315244

[13] Waters BM, McInturf SA, Amundsen K. Transcriptomic and physiological characterization of the fefe mutant of melon (*Cucumis melo*) reveals new aspects of iron-copper crosstalk. New Phytol. 2014;203: 1128–1145. 10.1111/nph.12911 24975482 PMC4117724

[14] Rodríguez-Celma J, Pan IC, Li W, Lan P, Buckhout TJ, Schmidt W. The transcriptional response of *Arabidopsis* leaves to Fe deficiency. Front Plant Sci. 2013;4: 276 10.3389/fpls.2013.00276 23888164 PMC3719017

[15] Tauris B, Borg S, Gregersen PL, Holm PB. A roadmap for zinc trafficking in the developing barley grain based on laser capture microdissection and gene expression profiling. J Exp Bot. 2009;60: 1333–1347. 10.1093/jxb/erp023 19297552 PMC2657541

[16] Darbani b, Stewart CN. Reproducibility and reliability assays of the gene expression-measurements. Journal of Biological Research-Thessaloniki 2014;21: 3. 10.1186/2241-5793-21-3 PMC4376515 25984486

[17] Darbani B, Stewart CN, Noeparvar S, Borg S. Correction of gene expression data: Performance-dependency on inter-replicate and inter-treatment biases. J Biotechnol. 2014;188: 100–109. 10.1016/j.jbiotec.2014.08.012 25150216

[18] Fan H, Zhang Z, Wang N, Cui Y, Sun H, Liu Y, et al SKB1/PRMT5-mediated histone H4R3 dimethylation of Ib subgroup bHLH genes negatively regulates iron homeostasis in *Arabidopsis thaliana* . Plant J. 2014;77: 209–221. 10.1111/tpj.12380 24298997

[19] Malapeira J, Mas P. A chromatin-dependent mechanism regulates gene expression at the core of the *Arabidopsis* circadian clock. Plant Signal Behav. 2013;8: e24079 10.4161/psb.24079 23470726 PMC3907418

[20] Li C, Xu J, Li J, Li Q, Yang H. Involvement of *Arabidopsis* histone acetyltransferase HAC family genes in the ethylene signaling pathway. Plant Cell Physiol. 2014 2;55(2):426–35. 10.1093/pcp/pct180 24287137

[21] Yu H, Luo N, Sun L, Liu D. HPS4/SABRE regulates plant responses to phosphate starvation through antagonistic interaction with ethylene signalling. J Exp Bot. 2012;63: 4527–4538. 10.1093/jxb/ers131 22615140 PMC3421987

[22] Kieber JJ, Rothenberg M, Roman G, Feldmann KA, Ecker JR. CTR1, a negative regulator of the ethylene response pathway in *Arabidopsis*, encodes a member of the raf family of protein kinases. Cell 1993;72: 427–441. 8431946 10.1016/0092-8674(93)90119-b

[23] Olmedo G, Guo H, Gregory BD, Nourizadeh SD, Aguilar-Henonin L, Li H, et al ETHYLENE-INSENSITIVE5 encodes a 5'—>3' exoribonuclease required for regulation of the EIN3-targeting F-box proteins EBF1/2. Proc Natl Acad Sci U S A. 2006;103: 13286–3293. 16920797 10.1073/pnas.0605528103 PMC1550774

[24] Binder BM, Walker JM, Gagne JM, Emborg TJ, Hemmann G, Bleecker AB, et al The *Arabidopsis* EIN3 binding F-Box proteins EBF1 and EBF2 have distinct but overlapping roles in ethylene signaling. Plant Cell 2007;19: 509–523. 17307926 10.1105/tpc.106.048140 PMC1867343

[25] Noh B, Murphy AS, Spalding EP. Multidrug resistance-like genes of *Arabidopsis* required for auxin transport and auxin-mediated development. Plant Cell 2001;13: 2441–2454. 11701880 10.1105/tpc.010350 PMC139463

[26] Zargar SM, Fujiwara M, Inaba S, Kobayashi M, Kurata R, Ogata Y, et al Correlation analysis of proteins responsive to Zn, Mn, or Fe deficiency in *Arabidopsis* roots based on iTRAQ analysis. Plant Cell Rep. 2015;34: 157–166. 10.1007/s00299-014-1696-2 25366567

[27] Cho M, Lee ZW, Cho HT. ATP-binding cassette B4, an auxin-efflux transporter, stably associates with the plasma membrane and shows distinctive intracellular trafficking from that of PIN-FORMED proteins. Plant Physiol. 2012;159: 642–654. 10.1104/pp.112.196139 22492845 PMC3375931

[28] Cheng Y, Dai X, Zhao Y. AtCAND1, a HEAT-repeat protein that participates in auxin signaling in *Arabidopsis* . Plant Physiol. 2004;135: 1020–1026. 15181201 10.1104/pp.104.044495 PMC514136

[29] Jain M, Kaur N, Garg R, Thakur JK, Tyagi AK, Khurana JP. Structure and expression analysis of early auxin-responsive Aux/IAA gene family in rice (*Oryza sativa*). Funct Integr Genomics. 2006;6: 47–59. 16200395 10.1007/s10142-005-0005-0

[30] del Pozo JC, Dharmasiri S, Hellmann H, Walker L, Gray WM, Estelle M. AXR1-ECR1-dependent conjugation of RUB1 to the *Arabidopsis* Cullin AtCUL1 is required for auxin response. Plant Cell. 2002;14: 421–433. 11884684 10.1105/tpc.010282 PMC152922

[31] Qi Y, Wang S, Shen C, Zhang S, Chen Y, Xu Y, et al OsARF12, a transcription activator on auxin response gene, regulates root elongation and affects iron accumulation in rice (*Oryza sativa*). New Phytol. 2012;193: 109–120. 10.1111/j.1469-8137.2011.03910.x 21973088

[32] Magidin M, Pittman JK, Hirschi KD, Bartel B. ILR2, a novel gene regulating IAA conjugate sensitivity and metal transport in *Arabidopsis thaliana* . Plant J. 2003;35: 523–534. 12904214 10.1046/j.1365-313x.2003.01826.x

[33] Bartel B, Fink GR. ILR1, an amidohydrolase that releases active indole-3-acetic acid from conjugates. Science. 1995;268: 1745–1748. 7792599 10.1126/science.7792599

[34] Lasswell J, Rogg LE, Nelson DC, Rongey C, Bartel B. Cloning and characterization of IAR1, a gene required for auxin conjugate sensitivity in *Arabidopsis* . Plant Cell 2000;12: 2395–2408. 11148286 10.1105/tpc.12.12.2395 PMC102226

[35] Rampey RA, Baldridge MT, Farrow DC, Bay SN, Bartel B. Compensatory mutations in predicted metal transporters modulate auxin conjugate responsiveness in *Arabidopsis* . G3 (Bethesda). 2013;3: 131–141. 23316445 10.1534/g3.112.004655 PMC3538338

[36] Staswick PE, Serban B, Rowe M, Tiryaki I, Maldonado MT, Maldonado MC, et al Characterization of an *Arabidopsis* enzyme family that conjugates amino acids to indole-3-acetic acid. Plant Cell 2005;17: 616–627. 15659623 10.1105/tpc.104.026690 PMC548830

[37] Schmidt W, Tittel J, Schikora A. Role of hormones in the induction of iron deficiency responses in *Arabidopsis* roots. Plant Physiol. 2000;122: 1109–1118. 10759506 10.1104/pp.122.4.1109 PMC58945

[38] Müller M, Schmidt W. Environmentally induced plasticity of root hair development in *Arabidopsis* . Plant Physiol. 2004;134: 409–419. 14730071 10.1104/pp.103.029066 PMC371035

[39] Giehl RF, Lima JE, von Wirén N. Localized iron supply triggers lateral root elongation in *Arabidopsis* by altering the AUX1-mediated auxin distribution. Plant Cell 2012;24: 33–49. 10.1105/tpc.111.092973 22234997 PMC3289578

[40] Guo HS, Xie Q, Fei JF, Chua NH. MicroRNA directs mRNA cleavage of the transcription factor NAC1 to downregulate auxin signals for *Arabidopsis* lateral root development. Plant Cell 2005;17: 1376–1386. 15829603 10.1105/tpc.105.030841 PMC1091761

[41] Doskočilová A, Plíhal O, Volc J, Chumová J, Kourová H, Halada P, et al A nodulin/glutamine synthetase-like fusion protein is implicated in the regulation of root morphogenesis and in signalling triggered by flagellin. Planta 2011;234: 459–476. 10.1007/s00425-011-1419-7 21533644

[42] Neuteboom LW, Ng JM, Kuyper M, Clijdesdale OR, Hooykaas PJ, van der Zaal BJ. Isolation and characterization of cDNA clones corresponding with mRNAs that accumulate during auxin-induced lateral root formation. Plant Mol Biol. 1999;39: 273–287. 10080694 10.1023/a:1006104205959

[43] Wang H, Lockwood SK, Hoeltzel MF, Schiefelbein JW. The ROOT HAIR DEFECTIVE3 gene encodes an evolutionarily conserved protein with GTP-binding motifs and is required for regulated cell enlargement in Arabidopsis. Genes Dev. 1997;11: 799–811. 9087433 10.1101/gad.11.6.799

[44] Nishino T, Okamoto K, Kawaguchi Y, Hori H, Matsumura T, Eger BT, et al Mechanism of the conversion of xanthine dehydrogenase to xanthine oxidase: identification of the two cysteine disulfide bonds and crystal structure of a non-convertible rat liver xanthine dehydrogenase mutant. J Biol Chem. 2005;280: 24888–24894. 15878860 10.1074/jbc.M501830200

[45] Bamdad F, Chen L. Antioxidant capacities of fractionated barley hordein hydrolysates in relation to peptide structures. Mol Nutr Food Res. 2013;57: 493–503. 10.1002/mnfr.201200252 23319431

[46] Grandbastien MA. Activation of plant retrotransposons under stress conditions. Trends Plant Sci. 1998;3: 181–187.

[47] Casacuberta E, González J. The impact of transposable elements in environmental adaptation. Mol Ecol. 2013;22: 1503–1517. 10.1111/mec.12170 23293987

[48] Stephan UW, Schmidke I, Pich A. Phloem translocation of Fe, Cu, Mn, and Zn in Ricinus seedlings in relation to the concentrations of nicotianamine, an endogenous chelator of divalent metal ions, in different seedling parts. Plant and Soil 1994;165: 181–188.

[49] Helliwell CA, Chin-Atkins AN, Wilson IW, Chapple R, Dennis ES, Chaudhury A. The *Arabidopsis* AMP1 gene encodes a putative glutamate carboxypeptidase. Plant Cell 2001;13: 2115–2125. 11549767 10.1105/TPC.010146 PMC139455

[50] Liu WZ, Kong DD, Gu XX, Gao HB, Wang JZ, Xia M, et al Cytokinins can act as suppressors of nitric oxide in *Arabidopsis* . Proc Natl Acad Sci U S A. 2013;110: 1548–1553. 10.1073/pnas.1213235110 23319631 PMC3557067

[51] Klaus SM, Kunji ER, Bozzo GG, Noiriel A, de la Garza RD, Basset GJ, et al Higher plant plastids and cyanobacteria have folate carriers related to those of trypanosomatids. J Biol Chem. 2005;280: 38457–38463. 16162503 10.1074/jbc.M507432200

[52] Ravanel S, Block MA, Rippert P, Jabrin S, Curien G, Rébeillé F, et al Methionine metabolism in plants: chloroplasts are autonomous for de novo methionine synthesis and can import S-adenosylmethionine from the cytosol. J Biol Chem. 2004;279: 22548–22557. 15024005 10.1074/jbc.M313250200

[53] Ranocha P, McNeil SD, Ziemak MJ, Li C, Tarczynski MC, Hanson AD. The S-methylmethionine cycle in angiosperms: ubiquity, antiquity and activity. Plant J. 2001;25: 575–284. 11309147 10.1046/j.1365-313x.2001.00988.x

[54] Sauter M, Moffatt B, Saechao MC, Hell R, Wirtz M. Methionine salvage and S-adenosylmethionine: essential links between sulfur, ethylene and polyamine biosynthesis. Biochem J. 2013;451: 145–154. 10.1042/BJ20121744 23535167

[55] Rangan P, Subramani R, Kumar R, Singh AK, Singh R. Recent advances in polyamine metabolism and abiotic stress tolerance. Biomed Res Int. 2014;2014: 239621 10.1155/2014/239621 25136565 PMC4124767

[56] García MJ, Lucena C, Romera FJ, Alcántara E, Pérez-Vicente R. Ethylene and nitric oxide involvement in the up-regulation of key genes related to iron acquisition and homeostasis in *Arabidopsis* . J Exp Bot. 2010;61: 3885–3899. 10.1093/jxb/erq203 20627899

[57] Wu Y, Zhao Q, Gao L, Yu XM, Fang P, Oliver DJ, et al Isolation and characterization of low-sulphur-tolerant mutants of *Arabidopsis* . J Exp Bot. 2010;61: 3407–3422. 10.1093/jxb/erq161 20547563 PMC2905201

[58] Santi S, Schmidt W. Dissecting iron deficiency-induced proton extrusion in *Arabidopsis* roots. New Phytol. 2009;183: 1072–1084. 10.1111/j.1469-8137.2009.02908.x 19549134

[59] Abdel-Ghany SE, Müller-Moulé P, Niyogi KK, Pilon M, Shikanai T. Two P-type ATPases are required for copper delivery in *Arabidopsis thaliana* chloroplasts. Plant Cell. 2005;17: 1233–1251. 15772282 10.1105/tpc.104.030452 PMC1087999

[60] Kim YY, Choi H, Segami S, Cho HT, Martinoia E, Maeshima M, et al AtHMA1 contributes to the detoxification of excess Zn(II) in *Arabidopsis* . Plant J. 2009;58: 737–753. 10.1111/j.1365-313X.2009.03818.x 19207208

[61] Morel M, Crouzet J, Gravot A, Auroy P, Leonhardt N, Vavasseur A, et al AtHMA3, a P1B-ATPase allowing Cd/Zn/Co/Pb vacuolar storage in *Arabidopsis* . Plant Physiol. 2009;149: 894–904. 10.1104/pp.108.130294 19036834 PMC2633814

[62] Zimmermann M, Clarke O, Gulbis JM, Keizer DW, Jarvis RS, Cobbett CS, et al Metal binding affinities of *Arabidopsis* zinc and copper transporters: selectivities match the relative, but not the absolute, affinities of their amino-terminal domains. Biochemistry. 2009;48: 11640–11654. 10.1021/bi901573b 19883117

[63] Deng F, Yamaji N, Xia J, Ma JF. A member of the heavy metal P-type ATPase OsHMA5 is involved in xylem loading of copper in rice. Plant Physiol. 2013;163: 1353–1362. 10.1104/pp.113.226225 24064929 PMC3813655

[64] Yamaji N, Xia J, Mitani-Ueno N, Yokosho K, Feng Ma J. Preferential delivery of zinc to developing tissues in rice is mediated by P-type heavy metal ATPase OsHMA2. Plant Physiol. 2013;162: 927–939. 10.1104/pp.113.216564 23575418 PMC3668081

[65] Podar D, Scherer J, Noordally Z, Herzyk P, Nies D, Sanders D. Metal selectivity determinants in a family of transition metal transporters. J Biol Chem. 2012;287: 3185–3196. 10.1074/jbc.M111.305649 22139846 PMC3270973

[66] Zientara K, Wawrzyńska A, Lukomska J, López-Moya JR, Liszewska F, Assunção AG, et al Activity of the AtMRP3 promoter in transgenic *Arabidopsis thaliana* and *Nicotiana tabacum* plants is increased by cadmium, nickel, arsenic, cobalt and lead but not by zinc and iron. J Biotechnol. 2009;139: 258–263. 10.1016/j.jbiotec.2008.12.001 19111837

[67] Gaillard S, Jacquet H, Vavasseur A, Leonhardt N, Forestier C. AtMRP6/AtABCC6, an ATP-binding cassette transporter gene expressed during early steps of seedling development and up-regulated by cadmium in *Arabidopsis thaliana* . BMC Plant Biol. 2008;8: 22 10.1186/1471-2229-8-22 18307782 PMC2291051

[68] Song WY, Park J, Mendoza-Cózatl DG, Suter-Grotemeyer M, Shim D, Hörtensteiner S, et al Arsenic tolerance in *Arabidopsis* is mediated by two ABCC-type phytochelatin transporters. Proc Natl Acad Sci U S A. 2010;107: 21187–21192. 10.1073/pnas.1013964107 21078981 PMC3000282

[69] Song WY, Mendoza-Cózatl DG, Lee Y, Schroeder JI, Ahn SN, Lee HS, et al Phytochelatin-metal(loid) transport into vacuoles shows different substrate preferences in barley and *Arabidopsis* . Plant Cell Environ. 2014;37: 1192–1201. 10.1111/pce.12227 24313707 PMC4123957

[70] Wu H, Ji Y, Du J, Kong D, Liang H, Ling HQ. ClpC1, an ATP-dependent Clp protease in plastids, is involved in iron homeostasis in *Arabidopsis* leaves. Ann Bot. 2010;105: 823–833. 10.1093/aob/mcq051 20382967 PMC2859920

[71] Huang CF, Yamaji N, Chen Z, Ma JF. A tonoplast-localized half-size ABC transporter is required for internal detoxification of aluminum in rice. Plant J. 2012;69: 857–867. 10.1111/j.1365-313X.2011.04837.x 22035218

[72] Curie C, Panaviene Z, Loulergue C, Dellaporta SL, Briat JF, Walker EL: Maize yellow stripe1 encodes a membrane protein directly involved in Fe(III) uptake. Nature 2001;409: 346–349. 11201743 10.1038/35053080

[73] Lubkowitz M. The oligopeptide transporters: a small gene family with a diverse group of substrates and functions? Mol Plant. 2011;4: 407–415. 10.1093/mp/ssr004 21310763

[74] Inoue H, Kobayashi T, Nozoye T, Takahashi M, Kakei Y, Suzuki K, et al Rice OsYSL15 is an iron-regulated iron(III)-deoxymugineic acid transporter expressed in the roots and is essential for iron uptake in early growth of the seedlings. J Biol Chem. 2009;284: 3470–3479. 10.1074/jbc.M806042200 19049971

[75] Lee S, Chiecko JC, Kim SA, Walker EL, Lee Y, Guerinot ML, et al Disruption of OsYSL15 leads to iron inefficiency in rice plants. Plant Physiol. 2009;150: 786–800. 10.1104/pp.109.135418 19376836 PMC2689993

[76] Chen CC, Chen YY, Tang IC, Liang HM, Lai CC, Chiou JM, et al *Arabidopsis* SUMO E3 ligase SIZ1 is involved in excess copper tolerance. Plant Physiol. 2011;156: 2225–2234. 10.1104/pp.111.178996 21632972 PMC3149952

[77] Zheng L, Yamaji N, Yokosho K, Ma JF. YSL16 is a phloem-localized transporter of the copper-nicotianamine complex that is responsible for copper distribution in rice. Plant Cell 2012;24: 3767–3782. 10.1105/tpc.112.103820 23012434 PMC3480301

[78] Lanquar V, Lelièvre F, Bolte S, Hamès C, Alcon C, Neumann D, et al Mobilization of vacuolar iron by AtNRAMP3 and AtNRAMP4 is essential for seed germination on low iron. EMBO J. 2005; 24: 4041–4051. 16270029 10.1038/sj.emboj.7600864 PMC1356305

[79] Lanquar V, Ramos MS, Lelièvre F, Barbier-Brygoo H, Krieger-Liszkay A, Krämer U, et al Export of vacuolar manganese by AtNRAMP3 and AtNRAMP4 is required for optimal photosynthesis and growth under manganese deficiency. Plant Physiol. 2010;152: 1986–1999. 10.1104/pp.109.150946 20181755 PMC2850043

[80] Oomen RJ, Wu J, Lelièvre F, Blanchet S, Richaud P, Barbier-Brygoo H, et al Functional characterization of NRAMP3 and NRAMP4 from the metal hyperaccumulator *Thlaspi caerulescens* . New Phytol. 2009;181: 637–650. 10.1111/j.1469-8137.2008.02694.x 19054339

[81] Curie C, Alonso JM, Le Jean M, Ecker JR, Briat JF. Involvement of NRAMP1 from *Arabidopsis thaliana* in iron transport. Biochem J. 2000;347 Pt 3: 749–55. 10769179 PMC1221012

[82] Gollhofer J, Schläwicke C, Jungnick N, Schmidt W, Buckhout TJ. Members of a small family of nodulin-like genes are regulated under iron deficiency in roots of *Arabidopsis thaliana* . Plant Physiol Biochem. 2011;49: 557–564. 10.1016/j.plaphy.2011.02.011 21411332

[83] Grotz N, Fox T, Connolly E, Park W, Guerinot ML, Eide D. Identification of a family of zinc transporter genes from *Arabidopsis* that respond to zinc deficiency. Proc Natl Acad Sci U S A. 1998;95: 7220–7224. 9618566 10.1073/pnas.95.12.7220 PMC22785

[84] Milner MJ, Seamon J, Craft E, Kochian LV. Transport properties of members of the ZIP family in plants and their role in Zn and Mn homeostasis. J Exp Bot. 2013; 64: 369–381. 10.1093/jxb/ers315 23264639 PMC3528025

[85] Tiong J, McDonald GK, Genc Y, Pedas P, Hayes JE, Toubia J, et al HvZIP7 mediates zinc accumulation in barley (*Hordeum vulgare*) at moderately high zinc supply. New Phytol. 2014;201: 131–143. 10.1111/nph.12468 24033183

[86] Trudeau MC, Zagotta WN. Calcium/calmodulin modulation of olfactory and rod cyclic nucleotide-gated ion channels. J Biol Chem. 2003;278: 18705–18708. 12626507 10.1074/jbc.R300001200

[87] Sunkar R, Kaplan B, Bouché N, Arazi T, Dolev D, Talke IN, et al Expression of a truncated tobacco NtCBP4 channel in transgenic plants and disruption of the homologous *Arabidopsis* CNGC1 gene confer Pb2+ tolerance. Plant J. 2000;24: 533–542. 11115134 10.1046/j.1365-313x.2000.00901.x

[88] Kanter U, Hauser A, Michalke B, Dräxl S, Schäffner AR. Caesium and strontium accumulation in shoots of *Arabidopsis thaliana*: genetic and physiological aspects. J Exp Bot. 2010;61: 3995–4009. 10.1093/jxb/erq213 20624763 PMC2935873

[89] Parry DA, Mighell AJ, El-Sayed W, Shore RC, Jalili IK, Dollfus H, et al Mutations in CNNM4 cause Jalili syndrome, consisting of autosomal-recessive cone-rod dystrophy and amelogenesis imperfecta. Am J Hum Genet. 2009;84: 266–273. 10.1016/j.ajhg.2009.01.009 19200525 PMC2668026

[90] Yamazaki D, Funato Y, Miura J, Sato S, Toyosawa S, Furutani K, et al Basolateral Mg2+ extrusion via CNNM4 mediates transcellular Mg2+ transport across epithelia: a mouse model. PLoS Genet. 2013;9: e1003983 10.1371/journal.pgen.1003983 24339795 PMC3854942

[91] Gibson MM, Bagga DA, Miller CG, Maguire ME. Magnesium transport in *Salmonella typhimurium*: the influence of new mutations conferring Co^2+^ resistance on the CorA Mg^2+^ transport system. Mol Microbiol. 1991;5: 2753–2762. 1779764 10.1111/j.1365-2958.1991.tb01984.x

[92] Smith RL, Banks JL, Snavely MD, Maguire ME. Sequence and topology of the CorA magnesium transport systems of *Salmonella typhimurium* and *Escherichia coli*. Identification of a new class of transport protein. J Biol Chem. 1993;268: 14071–14080. 8314774

[93] Tang RJ, Zhao FG, Garcia VJ, Kleist TJ, Yang L, Zhang HX, et al Tonoplast CBL-CIPK calcium signaling network regulates magnesium homeostasis in *Arabidopsis* . Proc Natl Acad Sci U S A. 2015;112: 3134–3139. 10.1073/pnas.1420944112 25646412 PMC4364200

[94] Smith MR, Boenzli MG, Hindagolla V, Ding J, Miller JM, Hutchison JE, Greenwood JA, Abeliovich H, Bakalinsky AT. Identification of gold nanoparticle-resistant mutants of *Saccharomyces cerevisiae* suggests a role for respiratory metabolism in mediating toxicity. Appl Environ Microbiol. 2013;79: 728–733. 10.1128/AEM.01737-12 23144132 PMC3553748

[95] Michaillat L, Mayer A. Identification of genes affecting vacuole membrane fragmentation in *Saccharomyces cerevisiae* . PLoS One 2013;8: e54160 10.1371/journal.pone.0054160 23383298 PMC3562189

[96] Shahriari M, Keshavaiah C, Scheuring D, Sabovljevic A, Pimpl P, Häusler RE, et al The AAA-type ATPase AtSKD1 contributes to vacuolar maintenance of *Arabidopsis thaliana* . Plant J. 2010;64: 71–85. 10.1111/j.1365-313X.2010.04310.x 20663085

[97] Cui Y, Zhao Q, Gao C, Ding Y, Zeng Y, Ueda T, et al Activation of the Rab7 GTPase by the MON1-CCZ1 complex is essential for PVC-to-vacuole trafficking and plant growth in *Arabidopsis* . Plant Cell 2014;26: 2080–2097. 24824487 10.1105/tpc.114.123141 PMC4079370

[98] McCreedy RA, Fleet JC. Forward genetics used to identify new gene Mon1a with critical role in controlling macrophage iron metabolism and iron recycling from erythrocytes. Nutr Rev. 2009; 67: 607–610. 10.1111/j.1753-4887.2009.00233.x 19785692 PMC2929669

[99] Bagley DC, Paradkar PN, Kaplan J, Ward DM. Mon1a protein acts in trafficking through the secretory apparatus. J Biol Chem. 2012;287: 25577–25588. 10.1074/jbc.M112.354043 22665492 PMC3408209

[100] Shi H, Bencze KZ, Stemmler TL, Philpott CC. A cytosolic iron chaperone that delivers iron to ferritin. Science 2008;320: 1207–1210. 10.1126/science.1157643 18511687 PMC2505357

[101] Frey AG, Nandal A, Park JH, Smith PM, Yabe T, Ryu MS, et al Iron chaperones PCBP1 and PCBP2 mediate the metallation of the dinuclear iron enzyme deoxyhypusine hydroxylase. Proc Natl Acad Sci U S A. 2014;111: 8031–8036. 10.1073/pnas.1402732111 24843120 PMC4050543

[102] Rodríguez-Cazorla E, Ripoll JJ, Andújar A, Bailey LJ, Martínez-Laborda A, Yanofsky MF, et al K-homology nuclear ribonucleoproteins regulate floral organ identity and determinacy in *Arabidopsis* . PLoS Genet. 2015;11: e1004983 10.1371/journal.pgen.1004983 25658099 PMC4450054

[103] Zhang Y, Wu H, Wang N, Fan H, Chen C, Cui Y, et al Mediator subunit 16 functions in the regulation of iron uptake gene expression in *Arabidopsis* . New Phytol. 2014;203: 770–783. 10.1111/nph.12860 24889527

[104] Salahudeen AA, Thompson JW, Ruiz JC, Ma HW, Kinch LN, Li Q, et al An E3 ligase possessing an iron-responsive hemerythrin domain is a regulator of iron homeostasis. Science 2009;326: 722–726. 10.1126/science.1176326 19762597 PMC3582197

[105] Chollangi S, Thompson JW, Ruiz JC, Gardner KH, Bruick RK. Hemerythrin-like domain within F-box and leucine-rich repeat protein 5 (FBXL5) communicates cellular iron and oxygen availability by distinct mechanisms. J Biol Chem. 2012;287: 23710–23717. 10.1074/jbc.M112.360404 22648410 PMC3390645

[106] Ruiz JC, Walker SD, Anderson SA, Eisenstein RS, Bruick RK. F-box and leucine-rich repeat protein 5 (FBXL5) is required for maintenance of cellular and systemic iron homeostasis. J Biol Chem. 2013;288: 552–560. 10.1074/jbc.M112.426171 23135277 PMC3537052

[107] Atwood SE, O'Rourke JA, Peiffer GA, Yin T, Majumder M, Zhang C, et al Replication protein A subunit 3 and the iron efficiency response in soybean. Plant Cell Environ. 2014;37: 213–234. 10.1111/pce.12147 23742135

[108] Ezaki B, Kiyohara H, Matsumoto H, Nakashima S. Overexpression of an auxilin-like gene (F9E10.5) can suppress Al uptake in roots of *Arabidopsis* . J Exp Bot. 2007;58: 497–506. 17150990 10.1093/jxb/erl221

[109] Lapierre LA, Kumar R, Hales CM, Navarre J, Bhartur SG, Burnette JO, et al Myosin vb is associated with plasma membrane recycling systems. Mol Biol Cell. 2001;12: 1843–1857. 11408590 10.1091/mbc.12.6.1843 PMC37346

[110] Carbone R, Fré S, Iannolo G, Belleudi F, Mancini P, Pelicci PG, et al eps15 and eps15R are essential components of the endocytic pathway. Cancer Res. 1997;57: 5498–5504. 9407958

[111] Barberon M, Zelazny E, Robert S, Conéjéro G, Curie C, Friml J, et al Monoubiquitin-dependent endocytosis of the iron-regulated transporter 1 (IRT1) transporter controls iron uptake in plants. Proc Natl Acad Sci U S A. 2011;108: E450–8. 10.1073/pnas.1100659108 21628566 PMC3156158

[112] Yamaji N, Sasaki A, Xia JX, Yokosho K, Ma JF. A node-based switch for preferential distribution of manganese in rice. Nat Commun. 2013;4: 2442 10.1038/ncomms3442 24048172

[113] Peñalver E, Lucero P, Moreno E, Lagunas R. Clathrin and two components of the COPII complex, Sec23p and Sec24p, could be involved in endocytosis of the *Saccharomyces cerevisiae* maltose transporter. J Bacteriol. 1999;181: 2555–2563. 10198022 10.1128/jb.181.8.2555-2563.1999 PMC93684

[114] Eguez L, Chung YS, Kuchibhatla A, Paidhungat M, Garrett S. Yeast Mn2+ transporter, Smf1p, is regulated by ubiquitin-dependent vacuolar protein sorting. Genetics 2004;167: 107–117. 15166140 10.1534/genetics.167.1.107 PMC1470849

[115] Fujita M, Maeda Y, Ra M, Yamaguchi Y, Taguchi R, Kinoshita T. GPI glycan remodeling by PGAP5 regulates transport of GPI-anchored proteins from the ER to the Golgi. Cell 2009;139: 352–365. 10.1016/j.cell.2009.08.040 19837036

[116] Vazquez HM, Vionnet C, Roubaty C, Conzelmann A. Cdc1 removes the ethanolamine phosphate of the first mannose of GPI anchors and thereby facilitates the integration of GPI proteins into the yeast cell wall. Mol Biol Cell. 2014;25: 3375–3388. 10.1091/mbc.E14-06-1033 25165136 PMC4214784

[117] Drdová EJ, Synek L, Pečenková T, Hála M, Kulich I, Fowler JE, et al The exocyst complex contributes to PIN auxin efflux carrier recycling and polar auxin transport in *Arabidopsis* . Plant J. 2013;73: 709–719. 10.1111/tpj.12074 23163883

[118] Barberon M, Dubeaux G, Kolb C, Isono E, Zelazny E, Vert G. Polarization of IRON-REGULATED TRANSPORTER 1 (IRT1) to the plant-soil interface plays crucial role in metal homeostasis. Proc Natl Acad Sci U S A. 2014;111: 8293–8298. 10.1073/pnas.1402262111 24843126 PMC4050562

[119] Fujimoto M, Suda Y, Vernhettes S, Nakano A, Ueda T. Phosphatidylinositol 3-kinase and 4-kinase have distinct roles in intracellular trafficking of cellulose synthase complexes in *Arabidopsis thaliana* . Plant Cell Physiol. 2015;56: 287–298. 10.1093/pcp/pcu195 25516570

[120] Bolger AM, Lohse M, Usadel B. Trimmomatic: A flexible trimmer for Illumina Sequence Data, Bioinformatics 2014;30: 2114–2120. 10.1093/bioinformatics/btu170 24695404 PMC4103590

[121] Langmead B, Salzberg S. Fast gapped-read alignment with Bowtie 2. Nat Methods. 2012;9: 357–359. 10.1038/nmeth.1923 22388286 PMC3322381

[122] Kim D, Pertea G, Trapnell C, Pimentel H, Kelley R, Salzberg SL. TopHat2: accurate alignment of transcriptomes in the presence of insertions, deletions and gene fusions. Genome Biol. 2011;14: R36. 10.1186/gb-2013-14-4-r36 PMC4053844 23618408

[123] Trapnell C, Roberts A, Goff L, Pertea G, Kim D, Kelley DR, et al Differential gene and transcript expression analysis of RNA-seq experiments with TopHat and Cufflinks. Nat Protoc. 2012;7: 562–578. 10.1038/nprot.2012.016 22383036 PMC3334321

[124] Li H, Handsaker B, Wysoker A, Fennell T, Ruan J, Homer N, et al The Sequence Alignment/Map format and SAMtools. Bioinformatics 2009;25: 2078–2079. 10.1093/bioinformatics/btp352 19505943 PMC2723002

[125] Xu G, Deng N, Zhao Z, Judeh T, Flemington E, Zhu D. SAMMate: a GUI tool for processing short read alignments in SAM/BAM format. Source Code Biol. Med. 2011;6: 2 10.1186/1751-0473-6-2 21232146 PMC3027120

[126] Robinson MD, McCarthy DJ, Smyth GK. edgeR: a Bioconductor package for differential expression analysis of digital gene expression data. Bioinformatics 2010;26: 139–140. 10.1093/bioinformatics/btp616 19910308 PMC2796818

[127] Benjamini Y, Hochberg Y. Controlling the false discovery rate: a practical and powerful approach to multiple testing. J. R. Statist. Soc. B. 1995;57: 289–300.

[128] Tang S, Li T, Cong P, Xiong W, Wang Z, Sun J. PlantLoc: an accurate web server for predicting plant protein subcellular localization by substantiality motif. Nucleic Acids Res. 2013;41(Web Server issue): W441–7. 10.1093/nar/gkt428 23729470 PMC3692052

