# Supplementary material for: Deciphering Mineral Homeostasis in Barley Seed Transfer Cells at Transcriptional Level
Source: PLoS One. 2015 Nov 4;10(11):e0141398. doi: 10.1371/journal.pone.0141398 (PMC4633283; doi:10.1371/journal.pone.0141398)
Supplement: S1 Fig — (PDF) [file pone.0141398.s001.pdf]

S1 Fig. Real-Time PCR was performed to confirm the expression changes found by RNA-Seq. Quantities are represented in the form of relative expression fold-changes. Real-Time PCR data was corrected based on the vacuolar ATP synthase [MLOC_59475] (**A**) and *Gadph* [MLOC_18233] (**B**) as reference genes. (**C**) The relative fold-change of RNA-Seq data are also shown. Three biological replicates (1-3) of samples including untreated sample (UT), 6 h after treatments (6Fe and 6Zn), and 24 h after treatments (24Fe and 24Zn) are shown in different colors.

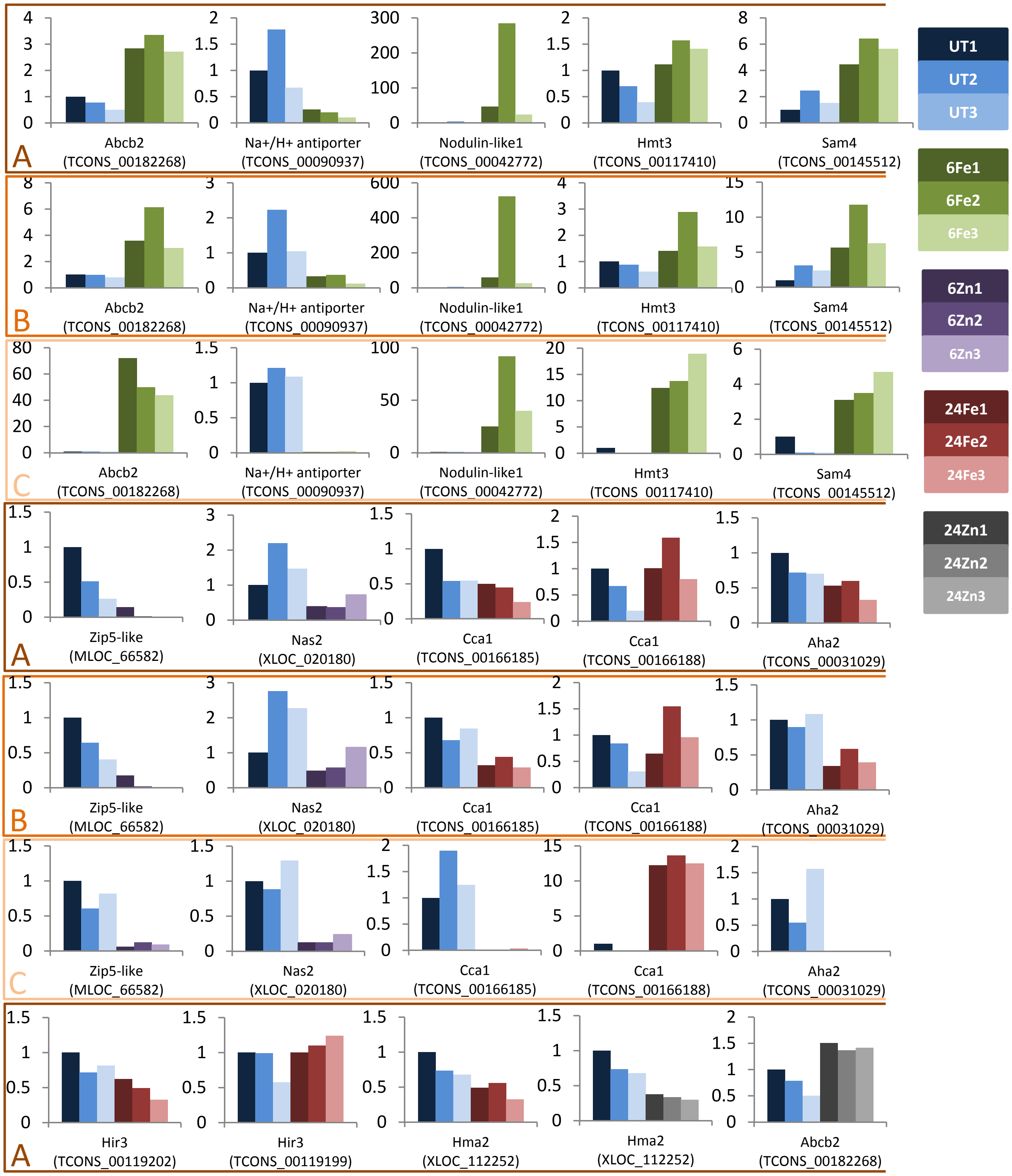
A
Abcb2 (TCONS_00182268)
Na+/H+ antiporter (TCONS_00090937)
Nodulin-like1 (TCONS_00042772)
Hmt3 (TCONS_00117410)
Sam4 (TCONS_00145512)
B
Abcb2 (TCONS_00182268)
Na+/H+ antiporter (TCONS_00090937)
Nodulin-like1 (TCONS_00042772)
Hmt3 (TCONS_00117410)
Sam4 (TCONS_00145512)
C
Abcb2 (TCONS_00182268)
Na+/H+ antiporter (TCONS_00090937)
Nodulin-like1 (TCONS_00042772)
Hmt3 (TCONS_00117410)
Sam4 (TCONS_00145512)
A
Zip5-like (MLOC_66582)
Nas2 (XLOC_020180)
Cca1 (TCONS_00166185)
Cca1 (TCONS_00166188)
Aha2 (TCONS_00031029)
B
Zip5-like (MLOC_66582)
Nas2 (XLOC_020180)
Cca1 (TCONS_00166185)
Cca1 (TCONS_00166188)
Aha2 (TCONS_00031029)
C
Zip5-like (MLOC_66582)
Nas2 (XLOC_020180)
Cca1 (TCONS_00166185)
Cca1 (TCONS_00166188)
Aha2 (TCONS_00031029)
A
Hir3 (TCONS_00119202)
Hir3 (TCONS_00119199)
Hma2 (XLOC_112252)
Hma2 (XLOC_112252)
Abcb2 (TCONS_00182268)
UT1
UT2
UT3
6Fe1
6Fe2
6Fe3
6Zn1
6Zn2
6Zn3
24Fe1
24Fe2
24Fe3
24Zn1
24Zn2
24Zn3

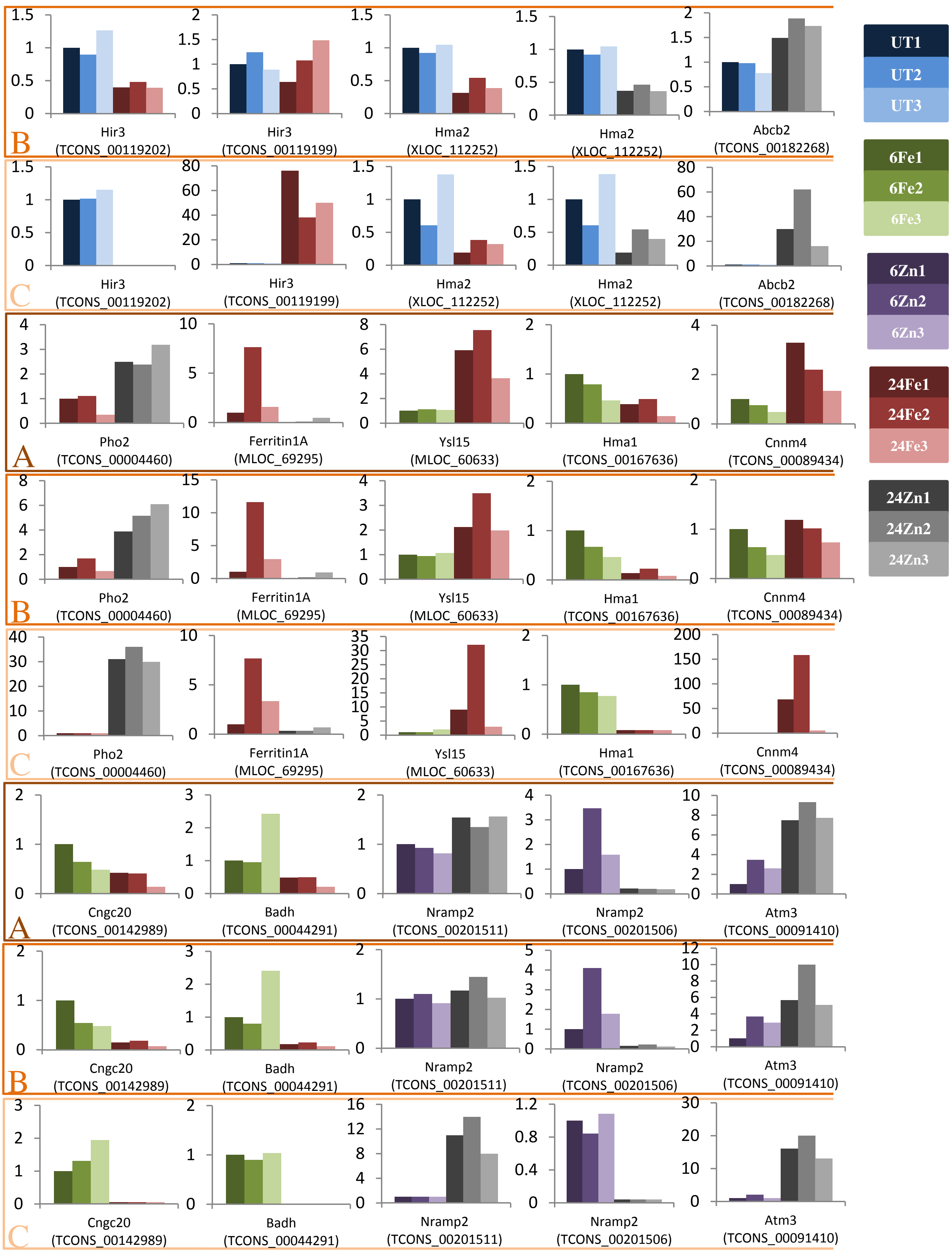
B
Hir3 (TCONS_00119202)
Hir3 (TCONS_00119199)
Hma2 (XLOC_112252)
Hma2 (XLOC_112252)
Abcb2 (TCONS_00182268)
C
Hir3 (TCONS_00119202)
Hir3 (TCONS_00119199)
Hma2 (XLOC_112252)
Hma2 (XLOC_112252)
Abcb2 (TCONS_00182268)
A
Pho2 (TCONS_00004460)
Ferritin1A (MLOC_69295)
Ysl15 (MLOC_60633)
Hma1 (TCONS_00167636)
Cnnm4 (TCONS_00089434)
B
Pho2 (TCONS_00004460)
Ferritin1A (MLOC_69295)
Ysl15 (MLOC_60633)
Hma1 (TCONS_00167636)
Cnnm4 (TCONS_00089434)
C
Pho2 (TCONS_00004460)
Ferritin1A (MLOC_69295)
Ysl15 (MLOC_60633)
Hma1 (TCONS_00167636)
Cnnm4 (TCONS_00089434)
A
Cngc20 (TCONS_00142989)
Badh (TCONS_00044291)
Nramp2 (TCONS_00201511)
Nramp2 (TCONS_00201506)
Atm3 (TCONS_00091410)
B
Cngc20 (TCONS_00142989)
Badh (TCONS_00044291)
Nramp2 (TCONS_00201511)
Nramp2 (TCONS_00201506)
Atm3 (TCONS_00091410)
C
Cngc20 (TCONS_00142989)
Badh (TCONS_00044291)
Nramp2 (TCONS_00201511)
Nramp2 (TCONS_00201506)
Atm3 (TCONS_00091410)
UT1
UT2
UT3
6Fe1
6Fe2
6Fe3
6Zn1
6Zn2
6Zn3
24Fe1
24Fe2
24Fe3
24Zn1
24Zn2
24Zn3

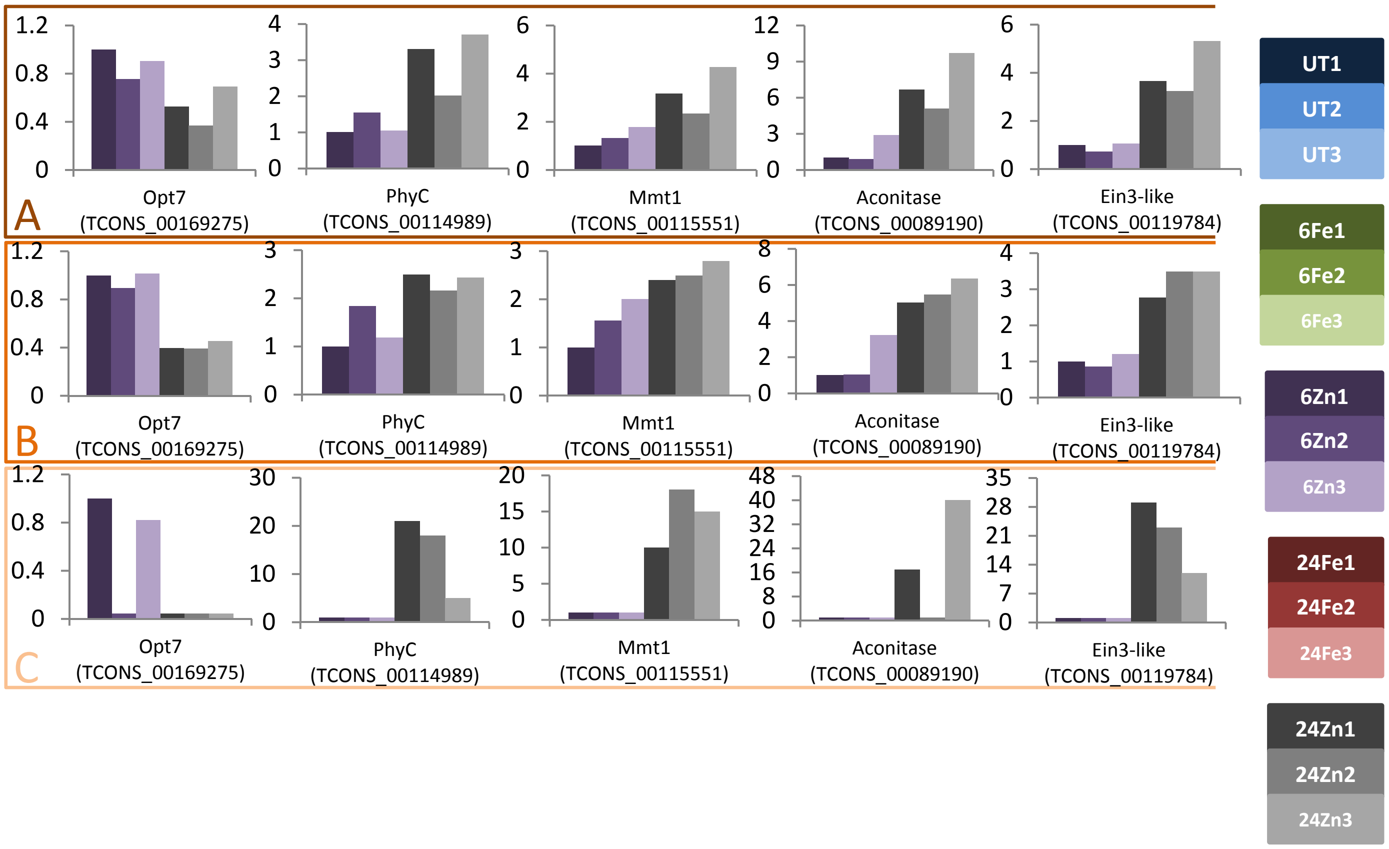
A
B
C
Opt7 (TCONS_00169275)
PhyC (TCONS_00114989)
Mmt1 (TCONS_00115551)
Aconitase (TCONS_00089190)
Ein3-like (TCONS_00119784)
UT1
UT2
UT3
6Fe1
6Fe2
6Fe3
6Zn1
6Zn2
6Zn3
24Fe1
24Fe2
24Fe3
24Zn1
24Zn2
24Zn3
