# Supplementary material for: Deciphering Mineral Homeostasis in Barley Seed Transfer Cells at Transcriptional Level
Source: PLoS One. 2015 Nov 4;10(11):e0141398. doi: 10.1371/journal.pone.0141398 (PMC4633283; doi:10.1371/journal.pone.0141398)
Supplement: S2 Fig — (PDF) [file pone.0141398.s002.pdf]

S2 Fig. Differentially expressed components of circadian rhythm. Most upstream, the expression of phytochrome C and light insensitive period 1 showed inverse transcriptional changes between the treatments. Phytochromes and other factors like LIP1 are involved in light input to the clock [Plant Signal Behav. 2011;6; 223-231]. The circadian clock associated 1, and time for coffee are active at downstream of photoreceptors [Plant Signal Behav. 2011;6; 223-231, Nat Cell Biol. 2011;13: 616-622]. The promoter activities of *Cca*1 and *Toc*1 are negatively correlated to iron availability [Plant Physiol. 2013;161: 893-903]. While one transcript of *Cca*1 was upregulated, two other alternative transcripts were properly repressed by iron. Downstream of PHYA, the imbibition-inducible 1 (*Imb*1), FAR1-related sequence 11-like, and scarecrow-like protein 8 also responded to the treatments. The transcription activator IMB1 regulates the ABA signaling negatively and PHYA signal transduction pathway positively [Plant J. 2003;35: 787-799]. Acting upstream of Constans and flowering locus T, the blue insensitive trait 1 and gigantea 2 were also repressed and induced, respectively. Covering different master regulators, our results pinpoint the dependency of mineral-demand on day/night cycle. Recalling the urgency of minerals for the light-dependent photosynthesis, these expressional changes highlight the importance of minerals for proper diurnal oscillation in plants.

Accession numbers of the genes are available in S4 File. Transcript isoforms of genes are distinguished by capital letters, immediately after the name of genes. Fe and Zn represent iron and zinc treatments. 6 and 24 represent samples of 6 h and 24 h after either of the treatments. UT stands for untreated sample. Comparisons of 24Fe/Untreated sample and 24Zn/Untreated sample are shown as 24Fe and 24Zn, respectively. Zinc treatment was compared with iron treatment which is shown as 6Zn/6Fe or 24Zn/24Fe. Including the function of gene, the data is accessible in S2 File. Phytochrome C (PhyC), Phytochrome A (PhyA), Light insensitive period 1 (Lip1), Imbibition-inducible 1 (Imb1), Scarecrow-like 8 (Scl8), FAR1-related sequences (Frs), Circadian clock associated 1 (CCA1), Blue insensitive trait 1 (Bit1), Gigantean (Gi), Constans (Co), Constans-like 9 and 10 (Col9 and 10), Homeobox protein luminidependens-like (Ld-like), FY-like, Frigida-like (Fri-like), Flowering Locus C (Flc), Cryptochrome 1 (Cry1), Flowering Locus T (Ft), WD-40 repeat-containing protein MSI4 gene (Msi4).

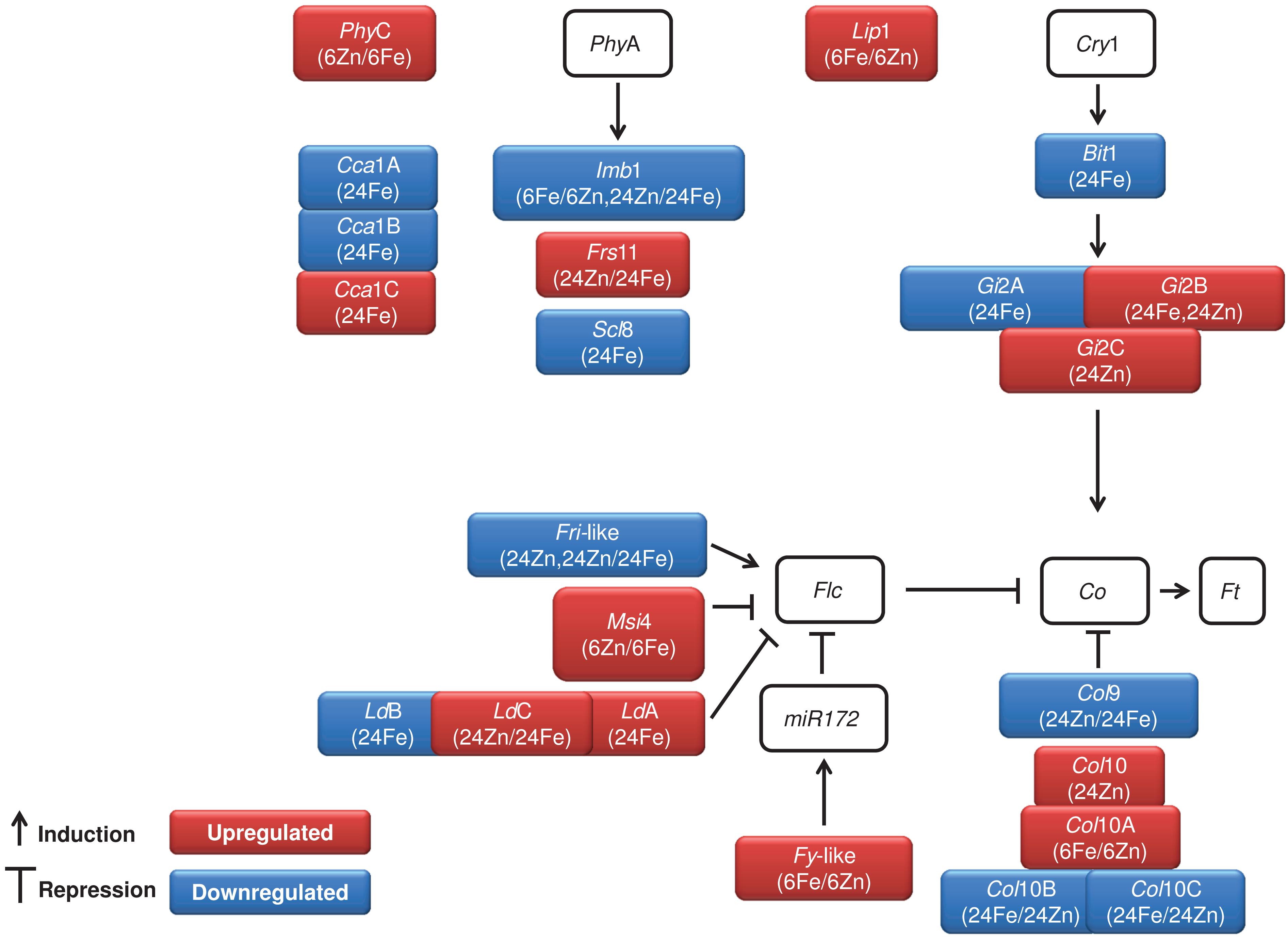
PhyC (6Zn/6Fe)
PhyA
Lip1 (6Fe/6Zn)
Cry1
Cca1A (24Fe)
Cca1B (24Fe)
Cca1C (24Fe)
Imb1 (6Fe/6Zn,24Zn/24Fe)
Frs11 (24Zn/24Fe)
Scl8 (24Fe)
Bit1 (24Fe)
Gi2A (24Fe)
Gi2B (24Fe,24Zn)
Gi2C (24Zn)
Fri-like (24Zn,24Zn/24Fe)
Msi4 (6Zn/6Fe)
LdB (24Fe)
LdC (24Zn/24Fe)
LdA (24Fe)
Flc
miR172
Fy-like (6Fe/6Zn)
Co
Ft
Col9 (24Zn/24Fe)
Col10 (24Zn)
Col10A (6Fe/6Zn)
Col10B (24Fe/24Zn)
Col10C (24Fe/24Zn)
Induction
Repression
Upregulated
Downregulated
