# Supplementary material for: Deciphering Mineral Homeostasis in Barley Seed Transfer Cells at Transcriptional Level
Source: PLoS One. 2015 Nov 4;10(11):e0141398. doi: 10.1371/journal.pone.0141398 (PMC4633283; doi:10.1371/journal.pone.0141398)
Supplement: S3 Fig — (PDF) [file pone.0141398.s003.pdf]

S3 Fig. Differentially expressed upstream regulatory genes involved in cellular stress response. Transcript isoforms of genes are distinguished by capital letters, immediately after the name of genes. Fe and Zn represent iron and zinc treatments and, 6 and 24 represent samples of 6 h and 24 h after either of the treatments. UT stands for untreated sample. Comparisons of 24Fe/Untreated sample and 24Zn/Untreated sample are shown as 24Fe and 24Zn, respectively. Zinc treatment was compared with iron treatment which is shown as 6Zn/6Fe or 24Zn/24Fe. Gene names and accession numbers are indicated in the S1 Text. ROS and ABA stand for reactive oxygen species and abscisic acid, respectively.

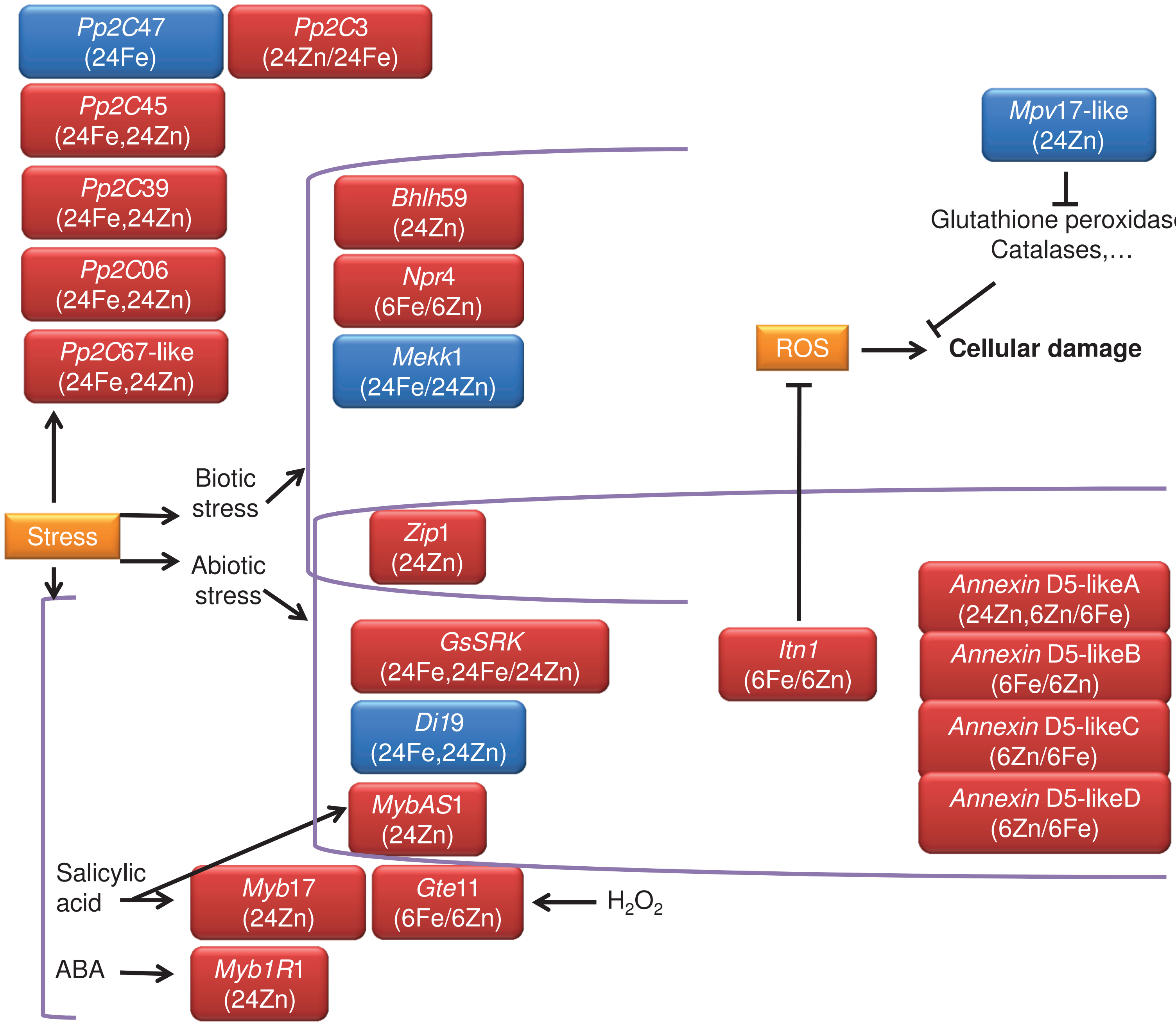
Pp2C47 (24Fe)
Pp2C3 (24Zn/24Fe)
Pp2C45 (24Fe,24Zn)
Pp2C39 (24Fe,24Zn)
Pp2C06 (24Fe,24Zn)
Pp2C67-like (24Fe,24Zn)
Bhlh59 (24Zn)
Npr4 (6Fe/6Zn)
Mekk1 (24Fe/24Zn)
Mpv17-like (24Zn)
Glutathione peroxidase
Catalases,…
ROS
Cellular damage
Stress
Biotic stress
Abiotic stress
Zip1 (24Zn)
GsSRK (24Fe,24Fe/24Zn)
Di19 (24Fe,24Zn)
MybAS1 (24Zn)
Itn1 (6Fe/6Zn)
Annexin D5-likeA (24Zn,6Zn/6Fe)
Annexin D5-likeB (6Fe/6Zn)
Annexin D5-likeC (6Zn/6Fe)
Annexin D5-likeD (6Zn/6Fe)
Salicylic acid
Myb17 (24Zn)
Gte11 (6Fe/6Zn)
H_2O_2
ABA
Myb1R1 (24Zn)

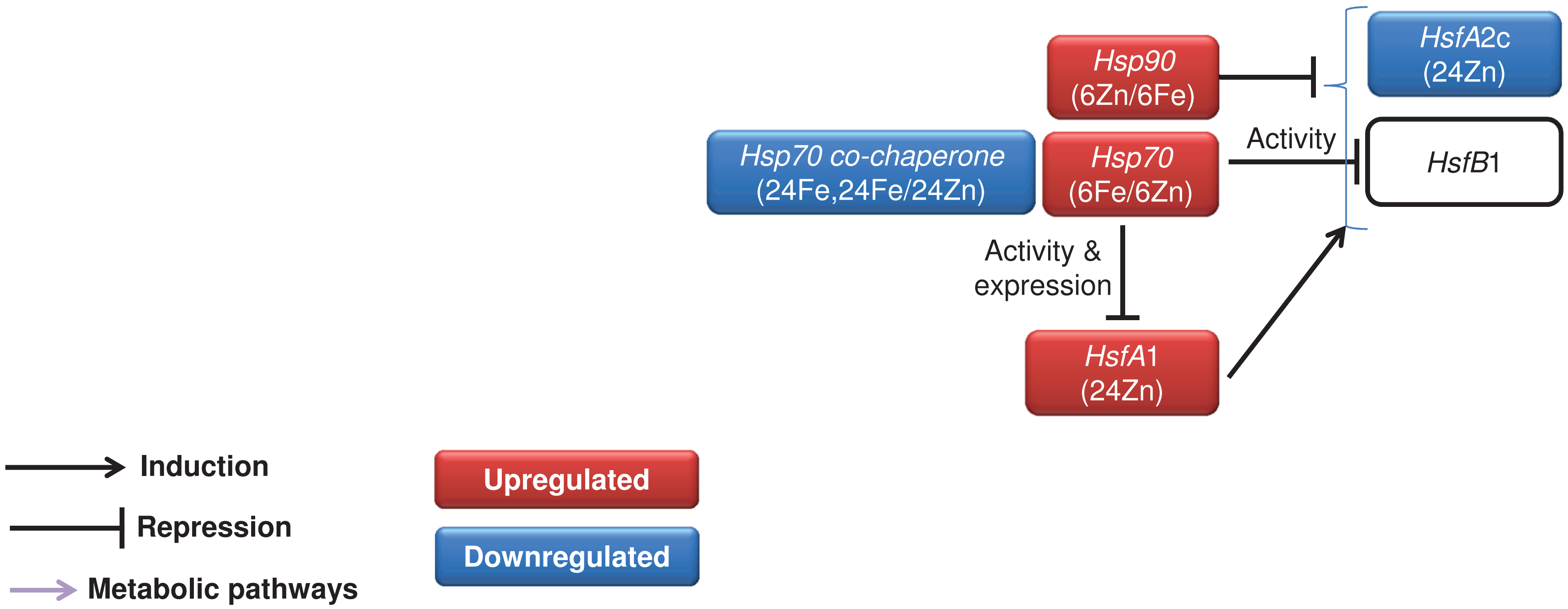

Hsp90 (6Zn/6Fe)
Hsp70 co-chaperone (24Fe,24Fe/24Zn)
Hsp70 (6Fe/6Zn)
Activity
HsfA2c (24Zn)
HsfB1
Activity & expression
HsfA1 (24Zn)
Induction
Repression
Metabolic pathways
Upregulated
Downregulated
