# Supplementary material for: Deciphering Mineral Homeostasis in Barley Seed Transfer Cells at Transcriptional Level
Source: PLoS One. 2015 Nov 4;10(11):e0141398. doi: 10.1371/journal.pone.0141398 (PMC4633283; doi:10.1371/journal.pone.0141398)

S4 Fig. Differentially expressed genes and transcripts involved in protein folding and degradation. 24Fe: 24 h after Fe treatment, 24Zn: 24 h after Zn treatment, and UT: untreated sample. For example, 24Fe/UT represents the comparison of 24Fe with UT.

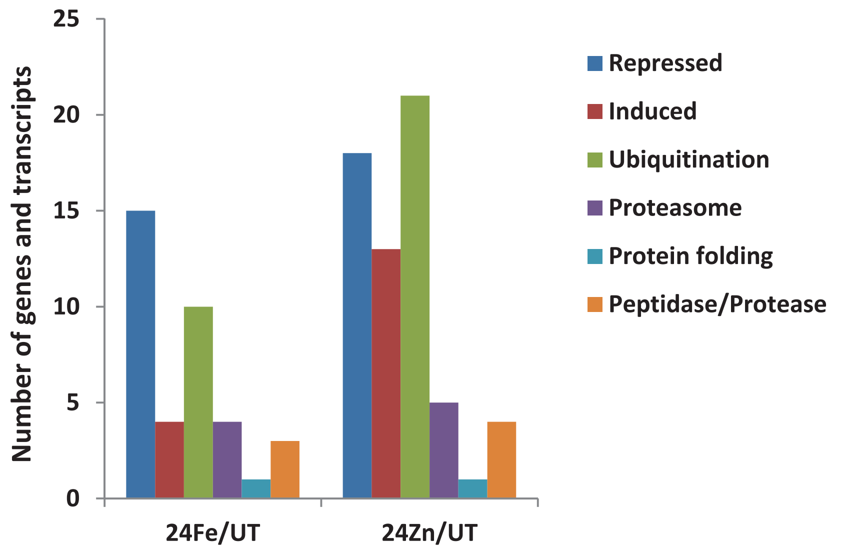

Supplement: S4 Fig — (PDF) [file pone.0141398.s004.pdf]
