# Supplementary material for: Deciphering Mineral Homeostasis in Barley Seed Transfer Cells at Transcriptional Level
Source: PLoS One. 2015 Nov 4;10(11):e0141398. doi: 10.1371/journal.pone.0141398 (PMC4633283; doi:10.1371/journal.pone.0141398)

S5 Fig. Chloroplastic tetrapyrrole, heme, and chlorophyll biosynthetic pathways were influenced after the treatments. Accession numbers of the genes are available in S4 File. Fe and Zn represent iron and zinc treatments and, 6 and 24 represent samples of 6 h and 24 h after either of the treatments. UT stands for untreated sample. Comparisons of 24Fe/Untreated sample and 24Zn/Untreated sample are shown as 24Fe and 24Zn, respectively. Zinc treatment was compared with iron treatment which is shown as 6Zn/6Fe or 24Zn/24Fe.

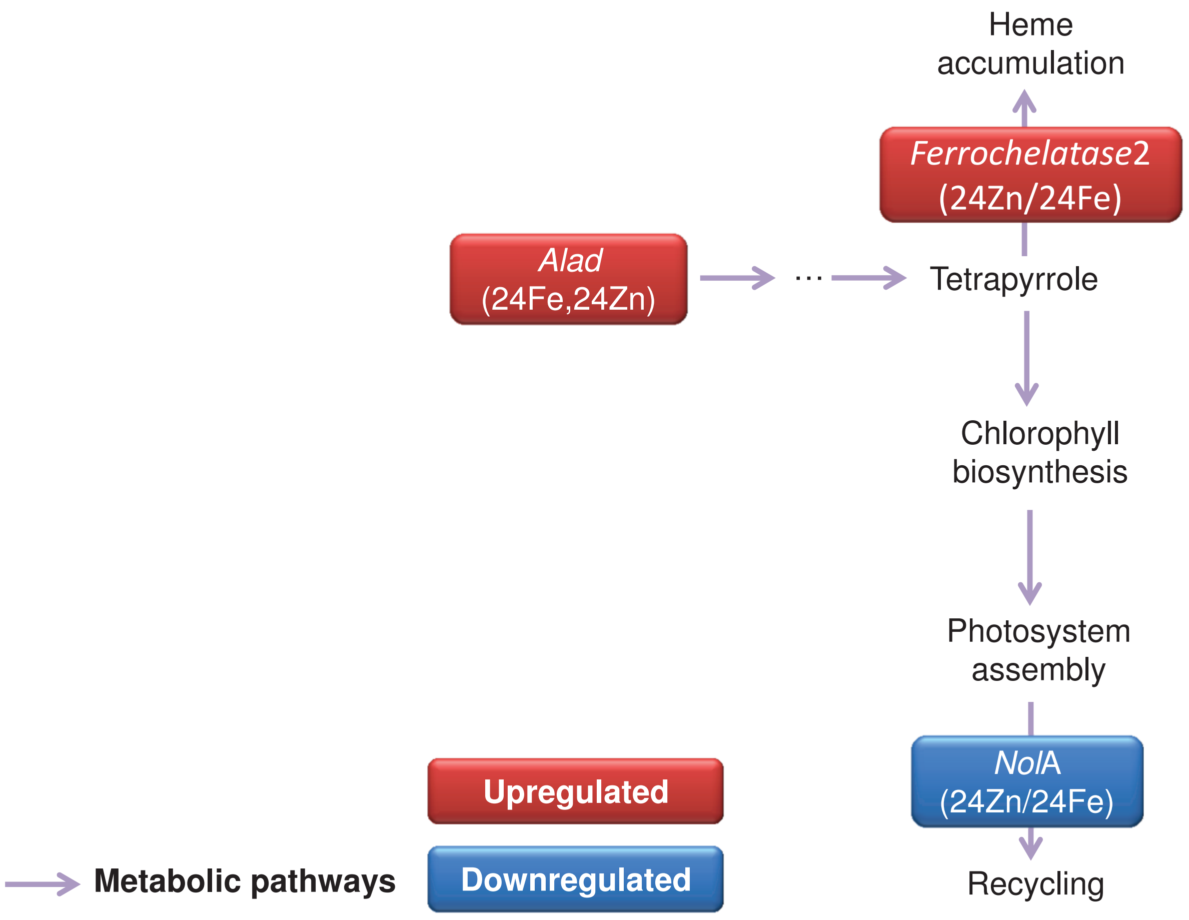

Supplement: S5 Fig — (PDF) [file pone.0141398.s005.pdf]
