# Supplementary material for: Deciphering Mineral Homeostasis in Barley Seed Transfer Cells at Transcriptional Level
Source: PLoS One. 2015 Nov 4;10(11):e0141398. doi: 10.1371/journal.pone.0141398 (PMC4633283; doi:10.1371/journal.pone.0141398)

S6A Fig. Per base sequence quality of the 15 samples including five treatments.

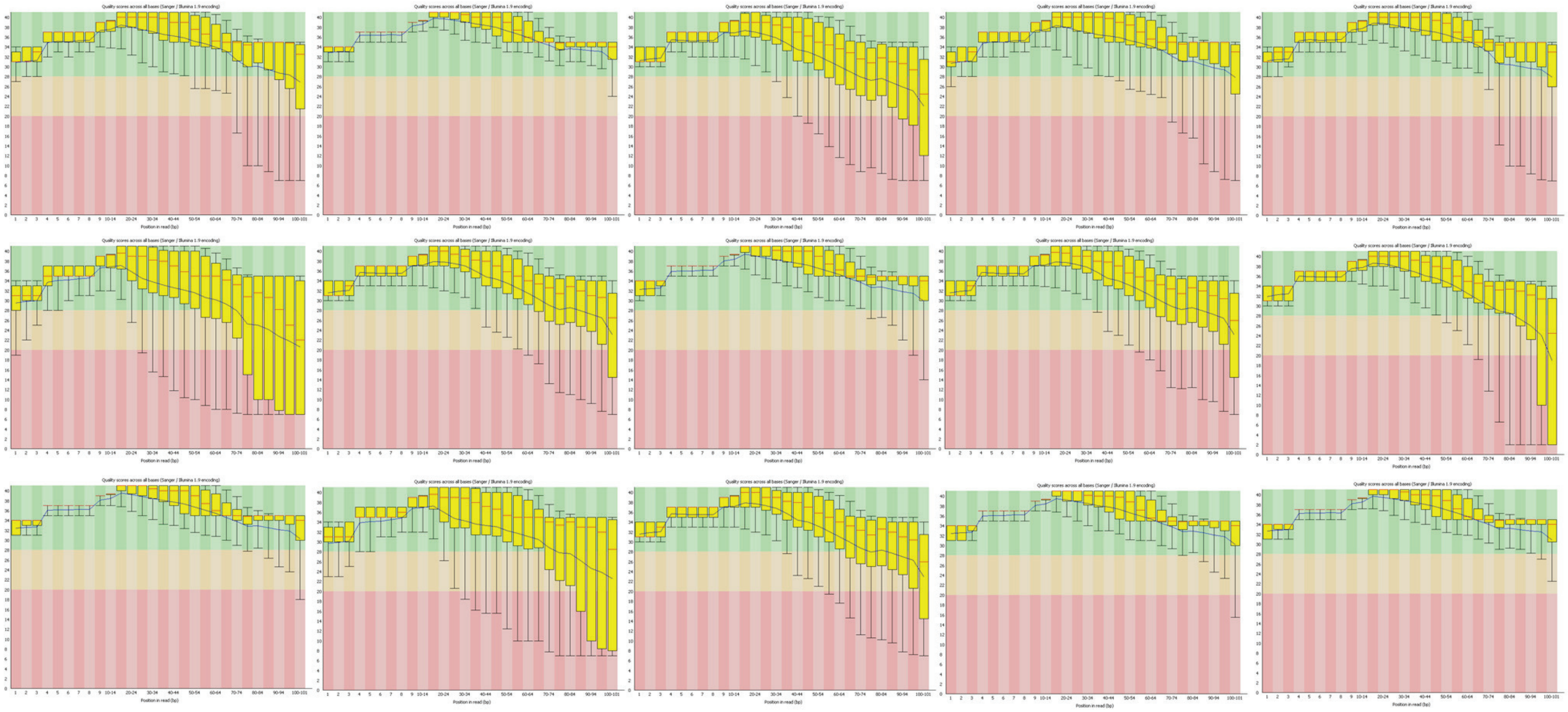

S6B Fig. Kmer content of the 15 samples including five treatments.

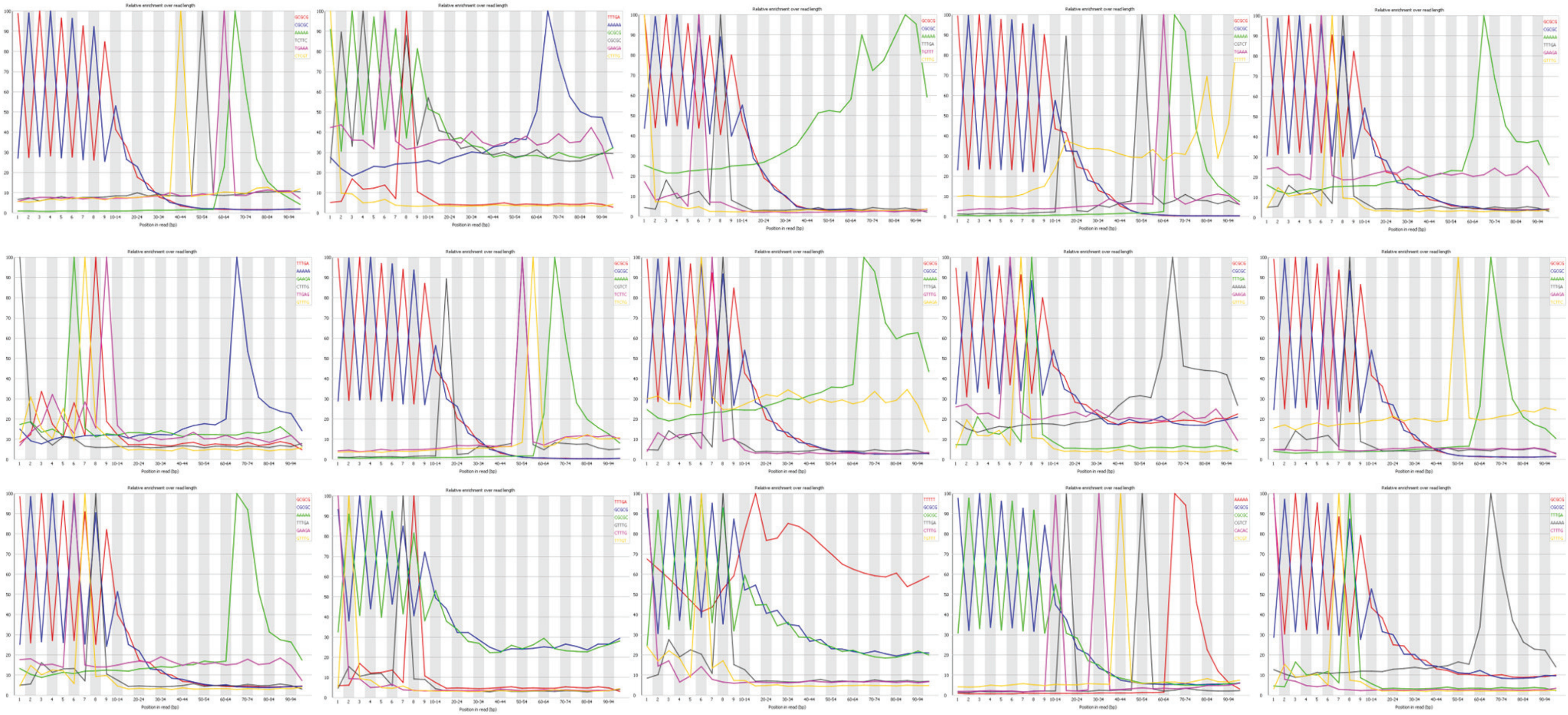

S6C Fig. Per base sequence content of the 15 samples including five treatments.

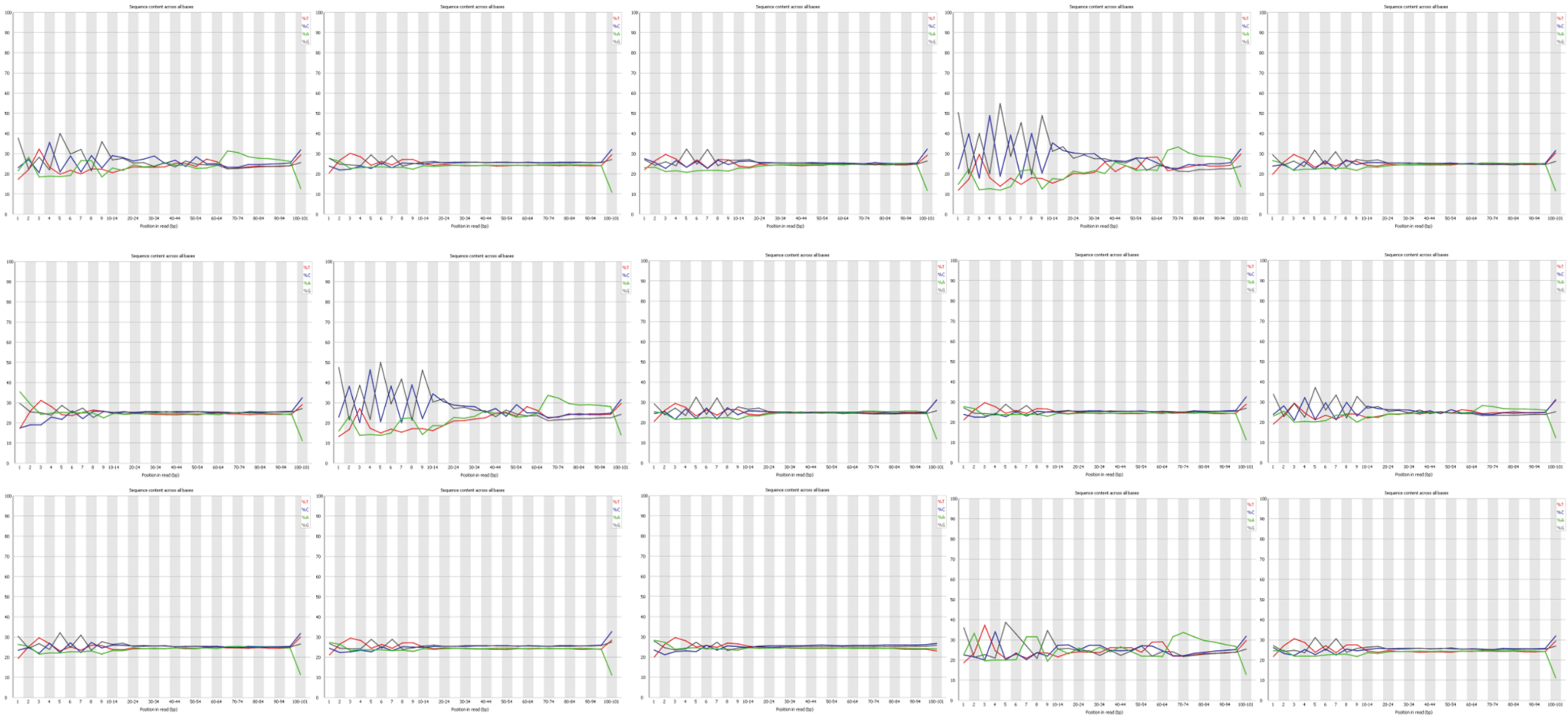

Supplement: S6 Fig — (PDF) [file pone.0141398.s006.pdf]
