# Supplementary material for: Deciphering Mineral Homeostasis in Barley Seed Transfer Cells at Transcriptional Level
Source: PLoS One. 2015 Nov 4;10(11):e0141398. doi: 10.1371/journal.pone.0141398 (PMC4633283; doi:10.1371/journal.pone.0141398)
Supplement: S1 File — We analyzed 43 publicly-available microarray datasets including 891 different barley samples to find the most stable reference genes. (PDF) [file pone.0141398.s007.pdf]

S1 File: Selected reference genes for bias structure analysis.

Publicly-available microarray data (http://www.ebi.ac.uk/arrayexpress/experiments/browse.html,) from 43 experiments (891 samples) were analyzed using the affymetrix expression console build 1.3.1.187 following the 3' Expression Arrays-RMA protocol. The quantile-based corrected data were used to measure the coefficient of variations of 22,840 events.

MLOC_59475 was selected as reference gene to correct the data (reference gene-based correction; see Fig. 2 and 3).
Gene accession numbers can be used to access the sequences at http://plants.ensembl.org/index.html.

Chr.	Gene accession	Microarray Probe	CV%
4HL	MLOC_74989	HV06H13u_x_at	7.49%
5HL	MLOC_38178	HVSMEl0009A21r2_at	7.53%
chr6	XLOC_084723	HVSMEl0006J08r2_at	7.56%
chr2	MLOC_36705	HW06E20u_x_at	8.12%
chr5	MLOC_14589	HV11I05u_x_at	8.48%
chr2	MLOC_51106	HV05H24u_x_at	7.81%
3HL	MLOC_19670	rbah15o14_at	8.23%
chr4	MLOC_16537	rbaal3o13_x_at	8.37%
chr7	MLOC_55795	HV02P13u_at	8.62%
chr4	MLOC_75234	Contig2591_at	8.71%
chr6	MLOC_4872	HVSMEg0007D18r2_at	8.96%
chr2	MLOC_67890	HV_CEb0022M04f_at	9.08%
5HL	MLOC_10688	Contig7890_at	9.33%
chr3	XLOC_034679	rbaal10d03_x_at	9.54%
chr4	MLOC_15010	HV08J24u_at	9.62%
chr2	MLOC_69938	HVSMEi0013G17f_at	9.72%
chr7	MLOC_54859	HV14M05r_at	6.41%
chr5	MLOC_59475	HA10P21u_at	6.37%

Microarray experiments:

E-GEOD-3170	E-GEOD-11182	E-GEOD-20416	E-GEOD-33397
E-GEOD-6325	E-GEOD-11200	E-GEOD-21804	E-GEOD-33398
E-GEOD-6990	E-GEOD-12584	E-GEOD-23595	E-GEOD-33401
E-GEOD-8618	E-GEOD-14521	E-GEOD-27821	E-GEOD-33405
E-GEOD-8712	E-GEOD-14930	E-GEOD-27822	E-GEOD-33406
E-GEOD-9365	E-GEOD-15295	E-GEOD-33391	E-GEOD-33407
E-GEOD-10328	E-GEOD-15970	E-GEOD-33393	E-GEOD-33938
E-GEOD-10330	E-GEOD-17238	E-GEOD-33394	E-GEOD-34102
E-GEOD-10332	E-GEOD-18758	E-GEOD-33396	E-GEOD-35639
E-GEOD-41511	E-GEOD-41512	E-GEOD-41513	E-GEOD-41514
E-GEOD-41515	E-GEOD-41516	E-GEOD-41517	
