# Supplementary material for: Deciphering Mineral Homeostasis in Barley Seed Transfer Cells at Transcriptional Level
Source: PLoS One. 2015 Nov 4;10(11):e0141398. doi: 10.1371/journal.pone.0141398 (PMC4633283; doi:10.1371/journal.pone.0141398)
Supplement: S4 File — (PDF) [file pone.0141398.s010.pdf]

S4 File: Accession numbers of the genes discussed in the main text.

Gene accession numbers can be used to access the sequences at http://plants.ensembl.org/index.html.

Genes	Accession numbers
Win1	MLOC_12747
Ctr1	MLOC_44066
Hps4	MLOC_3982 and MLOC_69563
Ebf1	MLOC_12878
Xrn4	MLOC_5783
Abcb2	MLOC_44063
Abcb19	MLOC_13299
Cand1	MLOC_67779
Iar1	XLOC_048523
Ilr1-like1	MLOC_51086
IAA-amido synthetase GH3.2	MLOC_5534 and XLOC_044856
Indole-3-acetic acid-amido synthetase GH3.5, JASMONATE RESISTANT 1, JAR1	MLOC_53684
Arf12-like	MLOC_51932
Arf4	MLOC_68907
Arf25	XLOC_051829
Arf9	MLOC_64596
Nac1	MLOC_53744
Air12	MLOC_13370
Phr1	MLOC_5585
Pho2	MLOC_53410
Spx1-like	MLOC_54859
Pho1-2-like	MLOC_12153
Pht2-1	MLOC_6818
G3Pp1	MLOC_14115
GDE1-like	MLOC_61338
Ckl2	MLOC_68238 and MLOC_19861
Wpk4	MLOC_74559
GA2	MLOC_12263
Nit4	MLOC_12498

Citrate transporter	MLOC_69350
Nitrate transporter 1.2-like	MLOC_58425
Ptr2	XLOC_007484, XLOC_063867, and MLOC_56891
PhyC	PhyC
Lip1	MLOC_76692
Imb1, imbibition-inducible 1	MLOC_55782
Scl8, scarecrow-like protein 8	MLOC_72117
Frs11, FAR1-related sequence 11-like	MLOC_15705
Cca1	MLOC_14118
Bit1, blue insensitive trait 1	XLOC_009824
Gigantea, Gi	MLOC_70638
Col9	MLOC_61919
Col10	MLOC_50966
Ld-like	MLOC_54055
FY-like	MLOC_65855
Frigida-like, Fri-like	MLOC_36986
bHLH135	MLOC_55964
Prl1	MLOC_57827
Npc1	MLOC_27455
Phosphoethanolamine N-methyltransferase 1	MLOC_66415
Aapt1	MLOC_53933
Aquaporin	MLOC_54419, MLOC_8032, and MLOC_14656
ABCG23	MLOC_61784
ABCG16	MLOC_10906
ABCG5	MLOC_66355
ABCG25	MLOC_62985
Pdr6	MLOC_62487
Pdr4	XLOC_036126
Pdr1	MLOC_5394
Hemerythrin-like domain containing protein coding gene	MLOC_51178
Phosphatidylinositol 3,4,5-trisphosphate 3-phosphatase	MLOC_17486
Phosphatidylinositol 4-kinase type 2-beta	MLOC_55934
LURP-one-related 6-like	MLOC_10773

pBI-1	MLOC_66886
Mitochondrial calcium uptake protein 1	MLOC_64237
Tonoplast calcium-transporting ATPase 4	MLOC_20538
Golgi/chloroplast calcineurin subunit B	MLOC_61171
Tonoplast anaerobic C4-dicarboxylate transporter	XLOC_073567
Plasma membrane proton pump AHA2	MLOC_75846
Plasma membrane pyrophosphate-energized proton pump-like	MLOC_6480
Plasma membrane potassium transporter 25-like	MLOC_63991
Tpk	MLOC_18521
Kob1	MLOC_65333
bHLH130-like	MLOC_67834
Tonoplast Na+/H+ antiporter, Nha	MLOC_14189
Alad	MLOC_74261
Chlorophyll b reductase NOL	MLOC_71939
Ferrochelatase-2	MLOC_59580
aarF domain-containing protein kinase	MLOC_37418
RuBisCO activase	XLOC_053055
RuBisCO large subunit Rbcl	rbcL-1 (EPlHVUG00000010074)
photosystem I P700 apoprotein A2, PsaB	XLOC_024496
photosystem I P700 apoprotein A1, PsaA	MLOC_67992
Photosystem II CP43	XLOC_042498
Photosystem II D1	MLOC_34266
Photosystem II protein D2	XLOC_094817
HvMcb1	MLOC_10280
Sweet11	MLOC_12385
Sweet4	MLOC_37708
Sweet13	MLOC_58163
Aconitase gene	MLOC_65580
NADH dehydrogenase iron-sulfur protein 2	XLOC_118602
Complex III assembly chaperone, BCS1-B	XLOC_080653
Pepc2	MLOC_10405
Cytochrome c oxidase subunit 1	MLOC_370
Cytochrome c oxidase subunit 3	MLOC_34123

NADH dehydrogenase subunit 4L	MLOC_9224
Apocytochrome b	MLOC_58118
Amp1	MLOC_68987
Chloroplastic folate-biopterin transporter Fbt1	MLOC_52745
Methionine synthase 1 , Ms1	MLOC_76000
Hmt3	MLOC_73966
Nas2	XLOC_020180
S-adenosylmethionine decarboxylase, AdoMetDC	MLOC_62478
Hma2	XLOC_112252
Abcc3 (Mrp3)	MLOC_37050
Abcc3 (Mrp3)	MLOC_59941
Mrp2	MLOC_56261
Abcc8-like	MLOC_52644
Abcb27, Als1	MLOC_10398
Ysl15	MLOC_60633
YSL16	XLOC_117521
E3 SUMO-protein ligase Siz1	MLOC_53617and MLOC_60545
Nramp3/4-like	MLOC_63231
Nramp2	MLOC_36948
Nodulin-like 2	XLOC_017866
Nodulin-like 1	XLOC_024234
Zip2	MLOC_77779
Zip7, homologue of AtZip4,	MLOC_56286
Cngc1	XLOC_108950
Cngc14	MLOC_7399
Mfsd5	MLOC_11806
Dentin sialophosphoprotein-like	MLOC_12212 and XLOC_030634
Cnnm4	MLOC_51948 and MLOC_71024
Auxilin-related protein 2	MLOC_14470
Myosin-Vb	MLOC_38101
Sec23A-like	MLOC_57586 and MLOC_10655
exocyst complex component 7, Exoc7	MLOC_56556
YMR155W-like	MLOC_59382

Ferritin 1A	MLOC_69295
Poly(rC)-binding protein 1-like, PCBP1-like	MLOC_64332
Poly(rC)-binding protein 2-like, PCBP2-like	MLOC_70283
Poly(rC) binding protein, PCBP	MLOC_60567
Cytosine-5-methyltransferase Cmt2-like	MLOC_59780
microRNA1126	XLOC_036446
Herc2	MLOC_12637
Epidermal growth factor receptor substrate 15-like	MLOC_58597
Rhd3	MLOC_60676
Mediator subunit 16, Med16	XLOC_001806
Atg7	MLOC_20377
COPII coat assembly protein sec16	MLOC_81720
RPA2, Replication protein A	MLOC_71848
TOR, Serine/threonine-protein kinase TOR	MLOC_13770
Eil3	MLOC_10315
YMR155W-like coding gene	MLOC_59382
Mtp1	MLOC_55970
Nodulin/Glutamine synthase-like	MLOC_59238
RabG3F	MLOC_70429
Plastid/mitochondrial pyruvate dehydrogenase	MLOC_53947
Plastidic acetyl-coA synthetase	MLOC_77651
LYR family of Fe/S cluster biogenesis protein	MLOC_65308
Fbt4	MLOC_54047
Eer4	MLOC_36649
Pdr9, Abcg37	MLOC_37495
K+, Na+ and Cl- symporter, Cation-chloride co-transporter 1	MLOC_54034
Vps4	MLOC_12988
Malic enzyme 3	MLOC_11548
Skb1	MLOC_76355
Malate dehydrogenase 2	MLOC_64140
MPP_Cdc1_like	MLOC_16478
Cipk23, CBL-interacting protein kinase 23	MLOC_61362
Cbl3	MLOC_16711

Ecr1	MLOC_14395
Hac1, Histone acetyltransferase of the CBP family 1	MLOC_11125
Histone-lysine N-methyltransferase ATXR3, Sdg2	MLOC_51413
CTP synthase	MLOC_11734 and MLOC_70060
Aspartate aminotransferase and glutamate/aspartate-prephenate aminotransferase	MLOC_14736
Glutamate receptor 3.4	MLOC_66590
Rho GTPase 1	MLOC_61507
Vip1	MLOC_71198
ClpC1	MLOC_73713
Rpa2	XLOC_034540
Rpa2	MLOC_15664
Glutathione synthetase, Gsh-s	MLOC_61045
