# Supplementary material for: Deciphering Mineral Homeostasis in Barley Seed Transfer Cells at Transcriptional Level
Source: PLoS One. 2015 Nov 4;10(11):e0141398. doi: 10.1371/journal.pone.0141398 (PMC4633283; doi:10.1371/journal.pone.0141398)
Supplement: S5 File — (PDF) [file pone.0141398.s011.pdf]

S5 File: Real-time PCR primers.

In case of transcript specific primers, the transcript identification number is provided.

Gene	Primer	Sequence 5′ to 3′
Abcb2, Auxin efflux transporter	Forward primer	CTGGTGCCAGGAAAGGTTCT
	Reverse primer	CTGAAAACAGGGCCTCCCTT
Na+/H+ antiporter precursor	Forward primer	ACATCTGATTTTCGCTGATGCT
	Reverse primer	TCCAATGCATCCATGCCAAC
Nodulin-like1	Forward primer	CTCTGGCTGTTCGTCAAGGT
	Reverse primer	ATTAGCCCGGCCTTACATCG
Hmt3, Homocysteine S-methyltransferase 3	Forward primer	ATGTAGGCGAGTGGTGCAAA
	Reverse primer	TACCTAAGCCGCAGGGTAGT
Sam4, S-adenosylmethionine synthase 4	Forward primer	AGGTGCATGTATCGTGGGTG
	Reverse primer	TCTACCGCGGAACGAAAAGA
Zip5-like	Forward primer	AGCATCTGATTGGCTGGTGG
	Reverse primer	GAAGAGCTGGTGGAACGTCA
Nas2, Nicotianamine synthase 2	Forward primer	GCCGGTGATCTTCTTCACCA
	Reverse primer	TAGTGCCCTCCTCTGCATCT
Cca1 (TCONS_00166185)	Forward primer	TGTTGCCCTGCTGATTGATT
	Reverse primer	TCTCTGCAACATTACTCATTGCT
Cca1 (TCONS_00166188)	Forward primer	CAGTGGACAACGCACCAAAG
	Reverse primer	TGCAACATGCTTGCTGTGTC
Aha1	Forward primer	CCATCCGTATTGTGCTTGGC
	Reverse primer	AGCTGTCGGGTAAGGGAGAT
Hir3 (TCONS_00119199)	Forward primer	AGATCCGGGCATTTCGTTGT
	Reverse primer	CCCATACTGCAGGCTTCCAA
Hir3 (TCONS_00119202)	Forward primer	CCCCCGGACGGAGGAA
	Reverse primer	TCCAGGTTGAGCTTCGGAAC
Hma2	Forward primer	GCAGCACAGAACACAAAGAGGA
	Reverse primer	CAACACCGGCGGACAACT
Pho2	Forward primer	AAGTTTTATACCTTGCCAAATTATTTATGGTT
	Reverse primer	AACTGTGGCTTCTCGGTCTG
Ferritin 1A	Forward primer	CGGGCAAGGGAAGATGTAGG

	Reverse primer	TCCCCCAAAACCACATCCAG
Ysl15	Forward primer	ATCCGGTGGATTTTTCGCCT
	Reverse primer	TTCGCATACTTGTGGGGCAT
Hma1	Forward primer	CGCTAAAGCTCGCCAAACAA
	Reverse primer	TGGAGAAGAACTGTCAACCAA
Cnnm4	Forward primer	AAGGGCCTCCCCTCTTTTTC
	Reverse primer	AGCTCTCCACCTTTTCCAGC
Cngc20	Forward primer	TGGCCCTGTCGACAAAATGA
	Reverse primer	CCTGCTTCCATCTGCACTGA
Badh	Forward primer	TGCTAAAGAGCCGATGCAAGA
	Reverse primer	GCACTGCAAACTTGACCACC
Nramp2 (TCONS_00201511)	Forward primer	CTCTTTGTCACAACTGTTTTCGCTA
	Reverse primer	ACCAAGGTTATCAGAGGAATCAGT
Nramp2 (TCONS_00201506)	Forward primer	GATTATTCATGTGCAGGTCTGTTTC
	Reverse primer	GATTTCCAACACTGCTCATTACCT
Atm3, ABC transporter B family member 25	Forward primer	TCGCTTCGTTGGTATGCAGT
	Reverse primer	AGGCACGTGACAAAGTTGGA
Opt7	Forward primer	GGAACTTACTCGGCGGTGAA
	Reverse primer	AACCAGCATCTAGTGCACCC
PhyC	Forward primer	GCTATGACAGGGTGATGGCA
	Reverse primer	CCTGGATGCTTGTGGGATGT
Aconitase	Forward primer	GTGGAGTTCCCCAATCACCA
	Reverse primer	GCAGGGCAATGAACCACATC
Ein3	Forward primer	GTAACATTATCGACGACTGACAATG
	Reverse primer	CTAATACGTGGGAAATATGCTACTGT
Mmt1	Forward primer	AGGAGGTAGGTGCTTCCAGT
	Reverse primer	TGAACCTCCCATGCTTGGTC
**Reference genes**	**Primer**	**Sequence 5′ to 3′**
Vacuolar H+-ATPase (MLOC_59475)	Forward primer	GATGCTGGGTGGGGGTTGAT
	Reverse primer	GCCAGACCCAACAGGCAAAT
Gadph (MLOC_18233)	Forward primer	CCAAGGCTGTTGGTAAGGTT
	Reverse primer	GACACATCCACAGTGGGAAC
