# Supplementary material for: Deciphering Mineral Homeostasis in Barley Seed Transfer Cells at Transcriptional Level
Source: PLoS One. 2015 Nov 4;10(11):e0141398. doi: 10.1371/journal.pone.0141398 (PMC4633283; doi:10.1371/journal.pone.0141398)
Supplement: S1 Table — (PDF) [file pone.0141398.s012.pdf]

S1 Table: Read mapping statistics.

Samples	1	2	3	4	5	6	7	8	9	10	11	12	13	14	15
**6FeR1**	32769072	7262436	58275708	12182618	20.91%	8845219	2855375	23883212	40.98%	13730231	57.49%	7088903	29.68%	6641328	27.81%
**6FeR2**	27075176	604929	53545423	12451502	23.25%	7777733	2960076	23189311	43.31%	13472947	58.10%	6562886	28.30%	6910061	29.80%
**6FeR3**	33358251	3838359	62878143	15516421	24.68%	10209685	2755269	28481375	45.30%	19554112	68.65%	9733564	34.17%	9820548	34.48%
**24FeR1**	22714324	11181254	34247394	7569608	22.10%	4509551	1415464	13494623	39.40%	9149629	67.80%	4298689	31.85%	4850940	35.94%
**24FeR2**	27473156	3761831	51184481	13838679	27.04%	8251255	2732076	24822010	48.50%	15242250	61.41%	11983283	48.28%	3258967	13.13%
**24FeR3**	45213714	2120889	88306539	19846813	22.47%	13287918	4316347	37451078	42.40%	19271006	51.46%	9521095	25.42%	9749911	26.03%
**6ZnR1**	26864826	16470209	37259443	5310422	14.25%	5044673	1295085	11650180	31.30%	8502592	72.98%	4914811	42.19%	3587781	30.80%
**6ZnR2**	26824339	2605497	51043181	13634341	26.71%	8249006	2773138	24656485	48.30%	15128165	61.36%	6997499	28.38%	8130666	32.98%
**6ZnR3**	40024375	1820078	78228672	20480174	26.18%	12292250	3929109	36701533	46.90%	22851055	62.26%	10870438	29.62%	11980617	32.64%
**24ZnR1**	32780274	6916914	58643634	16968429	28.93%	7888257	2886954	27743640	47.30%	20912962	75.38%	9310866	33.56%	11602096	41.82%
**24ZnR2**	29126461	2929503	55323419	14654234	26.49%	8800455	2906799	26361488	47.60%	15423882	58.51%	7129648	27.05%	8294234	31.46%
**24ZnR3**	67336974	1621730	133052218	30634990	23.02%	20858365	6297177	57790532	43.40%	29123276	50.40%	14053208	24.32%	15070068	26.08%
**UTR1**	39584932	373481	78796383	25346639	32.17%	10133564	3192213	38672416	49.10%	26690927	69.02%	11540407	29.84%	15150520	39.18%
**UTR2**	35039333	3153479	66925187	14514100	21.69%	10290443	3112576	27917119	41.70%	16006385	57.33%	7949273	28.47%	8057112	28.86%
**UTR3**	27966621	1579014	54354228	13171501	24.23%	8976621	2988402	25136524	46.20%	13784324	54.84%	6830869	27.17%	6953455	27.66%
**Avg.**	34276788	4415974	64137603	15741365	24.54%			28530102	44.48%	17256249.53	60.48%	8585696	30.10%	8670554	30.40%

1: Total read number (in pairs)
2: Quality failed reads by Tophat
3: Kept reads for alignment by TopHat
4: Number of Mapped reads
5: Fraction of reads were mapped
6: Number of reads were mapped after the first round read-trimming
7: Number of reads were mapped after the second round read-trimming
8: Total mapped reads (column4+column6+column7)
9: Total fraction of mapped reads
10: Total mapped reads on exonic region
11: Fraction of reads mapped on exonic region

12: Mapped reads on exon-exon junctions
13: Fraction of reads mapped on exon-exon junctions
14: Mapped on exons and not on junctions
15: Fraction of reads mapped on exons and not on junctions

UT: Untreated sample
6 & 24: Samples collected after 6 h and 24 h of the treatments
Fe & Zn: Iron and zinc treatments
R1- R3: Replicates
Avg.: Average
