# Supplementary material for: Deciphering Mineral Homeostasis in Barley Seed Transfer Cells at Transcriptional Level
Source: PLoS One. 2015 Nov 4;10(11):e0141398. doi: 10.1371/journal.pone.0141398 (PMC4633283; doi:10.1371/journal.pone.0141398)
Supplement: S2 Table — (PDF) [file pone.0141398.s013.pdf]

S2 Table: Differentially expressed genes involved in chromatin remodeling.

Gene accession numbers can be used to access the sequences at http://plants.ensembl.org/index.html.
6Fe: 6 h after Fe treatment, 6Zn: 6 h after Zn treatment, 24Fe: 24 h after Fe treatment, 24Zn: 24 h after Zn treatment, and UT: untreated sample. For example, 24Fe/UT represents the comparison of 24Fe with UT.

Genes/transcripts showing significant expression changes after the treatments

		Log$_2$ fold-changes	
Genea accession	Transcript	24Fe/UT	24Zn/UT
MLOC_10078	TCONS_00130749		-7.893
MLOC_10620	TCONS_00005487		8.333
MLOC_11125	TCONS_00144800	10.377	
MLOC_13090	TCONS_00112969		6.084
MLOC_51413	TCONS_00181824	6.223	5.129
MLOC_58535	TCONS_00052102		-7.470
MLOC_62732	TCONS_00033219	-9.118	-9.097
MLOC_68591	TCONS_00005037	8.794	8.299
MLOC_68591	TCONS_00005044		-7.812
MLOC_70462	TCONS_00174521	-8.759	
MLOC_70462	TCONS_00174519		6.023
MLOC_78810	TCONS_00056319		-7.667
XLOC_007299	TCONS_00014924		7.465
XLOC_099518	TCONS_00164899	11.087	9.834
XLOC_099548	TCONS_00165055		-7.697

For functional details see S2 File.

Genes /transcripts showing significant different expression patterns when comparing zinc with iron

		Log$_2$ fold-changes	
Genea accession	Transcript	6Zn/6Fe	24Zn/24Fe
MLOC_10030	TCONS_00092578	-7.536	-7.436
MLOC_10030	TCONS_00092583	8.968	
MLOC_10620	TCONS_00005480		-8.381
MLOC_11299	TCONS_00060793	-8.380	
MLOC_11348	TCONS_00139391		-7.653

MLOC_12053	TCONS_00050751		-7.624
MLOC_12053	TCONS_00050748		4.792
MLOC_12102	TCONS_00064573		6.054
MLOC_12102	TCONS_00064569	7.222	
MLOC_13052	TCONS_00035418		-8.993
MLOC_34561	TCONS_00035225		-7.763
MLOC_34561	TCONS_00035219		7.178
MLOC_44316	TCONS_00121826		-8.030
MLOC_44316	TCONS_00121823		-7.533
MLOC_44316	TCONS_00121830		9.651
MLOC_44316	TCONS_00121826	-8.433	
MLOC_4555	TCONS_00062806		7.862
MLOC_51537	TCONS_00082558		7.649
MLOC_51537	TCONS_00082555	8.218	
MLOC_54689	TCONS_00112091		-5.707
MLOC_57410	TCONS_00062033	-9.387	9.239
MLOC_58445	TCONS_00092485	-7.687	
MLOC_58575	TCONS_00118039	-7.536	
MLOC_59895	TCONS_00112586		-8.157
MLOC_60134	TCONS_00028671	8.120	
MLOC_62732	TCONS_00033216		8.229
MLOC_68963	TCONS_00058957		-8.095
MLOC_69040	TCONS_00100347		4.932
MLOC_72504	TCONS_00008210		7.620
XLOC_024260		-4.024	
MLOC_4670	TCONS_00168367		-5.181
MLOC_76355	TCONS_00140313		-7.533
MLOC_68482	TCONS_00055753		-8.935
MLOC_10278	TCONS_00110890		-7.333
MLOC_4846	TCONS_00057759		7.293

For functional details see S2 File.
