# Supplementary material for: Deciphering Mineral Homeostasis in Barley Seed Transfer Cells at Transcriptional Level
Source: PLoS One. 2015 Nov 4;10(11):e0141398. doi: 10.1371/journal.pone.0141398 (PMC4633283; doi:10.1371/journal.pone.0141398)
Supplement: S4 Table — (PDF) [file pone.0141398.s015.pdf]

S4 Table: Differentially expressed genes involved in DNA-damage response.

Gene accession numbers can be used to access the sequences at http://plants.ensembl.org/index.html.

24Fe: 24 h after Fe treatment, 24Zn: 24 h after Zn treatment, and UT: untreated sample.

For example, 24Fe/UT represents the comparison of 24Fe with UT.

Genea accession n	Transcript ID	24Fe/UT (Log2 fold-changes)
MLOC_80839	TCONS_00165175	-10.831
MLOC_52203	TCONS_00087144	-9.495
MLOC_70155	TCONS_00147142	-8.730
MLOC_12301	TCONS_00027923	8.615
MLOC_80839	TCONS_00165173	8.708
MLOC_52203	TCONS_00087139	9.181

Genea accession n	Transcript ID	24Zn/UT (Log2 fold-changes)
MLOC_44144	TCONS_00050762	-9.533
MLOC_50473	TCONS_00006737	-7.636
MLOC_52203	TCONS_00087144	-6.391
MLOC_52203	TCONS_00087135	7.303
MLOC_36617	TCONS_00173901	7.402
XLOC_048568	TCONS_00082563	8.282
MLOC_52203	TCONS_00087143	8.982
MLOC_9934	TCONS_00063425	9.798

For functional details see S2 File.
