# Supplementary material for: Deciphering Mineral Homeostasis in Barley Seed Transfer Cells at Transcriptional Level
Source: PLoS One. 2015 Nov 4;10(11):e0141398. doi: 10.1371/journal.pone.0141398 (PMC4633283; doi:10.1371/journal.pone.0141398)
Supplement: S6 Table — (PDF) [file pone.0141398.s017.pdf]

S6 Table: Differentially expressed genes positively involved in biotic stress response.

Gene accession numbers can be used to access the sequences at http://plants.ensembl.org/index.html.

24Fe: 24 h after Fe treatment, 24Zn: 24 h after Zn treatment, and UT: untreated sample. For example, 24Fe/UT represents the comparison of 24Fe with UT.

Genea accession n	Transcript	24Fe/UT (Log2 fold-changes)
MLOC_44740		3.31
MLOC_44740		3.31
XLOC_125552		2.91
XLOC_102179		6.00
XLOC_042786	TCONS_00073705	8.62
HIR3	TCONS_00119202	-10.37
MLOC_37056		-2.84
XLOC_032316		2.01
MLOC_72278		2.13
XLOC_051490		2.78
MLOC_22225		2.80
MLOC_22225		2.80
MLOC_65022		3.02
MLOC_22225	TCONS_00009376	3.11
MLOC_22225	TCONS_00009376	3.11
HIR3	TCONS_00119199	8.72
HIR3	TCONS_00119198	9.18

Genea accession n	Transcript	24Zn/UT (Log2 fold-changes)
MLOC_91	TCONS_00130079	-8.32
MLOC_10090		-6.38
MLOC_64217	TCONS_00030509	-7.76
MLOC_61466	TCONS_00007855	-7.54
MLOC_59797	TCONS_00205019	-5.95
MLOC_60268	TCONS_00117590	-5.54
MLOC_55862	TCONS_00118472	-5.38
MLOC_12349		-1.33
MLOC_52965		-1.19
MLOC_22225		1.16
MLOC_44740		1.48
XLOC_068681	TCONS_00116134	7.61

For functional details see S2 File.
