# Supplementary material for: Deciphering Mineral Homeostasis in Barley Seed Transfer Cells at Transcriptional Level
Source: PLoS One. 2015 Nov 4;10(11):e0141398. doi: 10.1371/journal.pone.0141398 (PMC4633283; doi:10.1371/journal.pone.0141398)
Supplement: S7 Table — (PDF) [file pone.0141398.s018.pdf]

S7 Table: Agilent 2100 bioanalyzer was used to check the quantities and qualities of RNA samples.

Samples	Concentration (pg/μl)	RNA integrity (RIN)
UT1	315	3.1
UT2	633	7.1
UT3	418	8
6hFe1	300	5.8
6hFe2	330	4.9
6hFe3	357	7.2
24hFe1	130	6.8
24hFe2	312	7.3
24hFe3	240	5.2
6hZn1	160	4.7
6hZn2	250	6.1
6hZn3	305	7
24hZn1	650	3.6
24hZn2	234	6.7
24hZn3	150	6.5

Fe, Zn, and UT represent iron and zinc treated and untreated samples, respectively. The 6 h and 24 h represent collected samples 6 h and 24 h after the treatments. Numbers 1, 2, and 3 represent three replicates of each sample.
