# Supplementary material for: Deciphering Mineral Homeostasis in Barley Seed Transfer Cells at Transcriptional Level
Source: PLoS One. 2015 Nov 4;10(11):e0141398. doi: 10.1371/journal.pone.0141398 (PMC4633283; doi:10.1371/journal.pone.0141398)
Supplement: S1 Text — It describes some of the stress-response genes found differentially expressed after the treatments. (PDF) [file pone.0141398.s019.pdf]

Deciphering Mineral Homeostasis in Barley Seed Transfer Cells at Transcriptional Level

Behrooz Darbani[1,2¶]*, Shahin Noeparvar[1¶], Søren Borg[1]*

[1] Department of Molecular Biology and Genetics, Research Centre Flakkebjerg, Aarhus University, Slagelse, Denmark
[2] Department of Plant and Environmental Sciences, University of Copenhagen, Frederiksberg, Denmark.
* Corresponding authors
Email: behroozdarbani@gmail.com (BD), soren.borg@mbg.au.dk (SB)

S1 Text

Iron- and zinc-triggered stress responses

Detoxification mechanisms

If not scavenged, reactive oxygen species can lead to oxidative destruction of cells. Catalases, dismutases, and ascorbate peroxidases are the major ROS scavengers (Trends in Plant Science 2002, 7:405-410). As detoxifying enzymes, different peroxidases were found induced by comparing iron and zinc treatments.

Catalase 2

The heme containing catalase 2 detoxifies hydrogen peroxide (Plant Mol Biol. 1995, 29:1005-1014). *HvCat*2 gene (MLOC_11835) had higher expression by iron treatment compared to 24 h after zinc treatment.

L-ascorbate peroxidase 2

The heme and calcium binding L-ascorbate peroxidase 2 coding gene (*Apx*2; MLOC_69302) was induced strongly by zinc treatment after 24 h. It is involved in hydrogen peroxide removal and stress response (Gene 2003, 322:93-103).

Peroxidases

The peroxidase 12 (*Per*12; XLOC_027592) showed higher expression by iron after 6 h compared to zinc and one isoform of the peroxidase 5-like gene (XLOC_052491) had higher expression after 24 h of zinc treatment compared to iron. In contrast, the peroxidase 4 gene (MLOC_64966) was repressed after 24 h of iron treatment.

Aldehyde dehydrogenase

As a stress response factor, aldehyde dehydrogenase family 7 member A1 (Antiquitin-1; MLOC_72011) was repressed after 24 h of the treatments. It can detoxify aldehydes and scavenge ROS (see Plant Cell Physiol. 1999, 40: 198-204; Plant Cell & Environment 2006, 29:1033-1048).

Xanthine dehydrogenase

Two Xanthine dehydrogenase genes (MLOC_11603 and XLOC_002008) were induced each in one transcript after 24 h of zinc treatment. Xanthine dehydrogenase catalyzes the oxidation of hypoxanthine to xanthine and the xanthine to uric acid which has been proposed as an important scavenger of deleterious oxygen species and peroxynitrite in biological systems (J Am Aging Assoc. 2001, 24:187-93; J Biol Chem. 2005, 280:24888-24894). Both of the isoforms do not contain the 2Fe-2S binding Fer2-2 domain. This is a wise cellular reaction to manage the oxidative stress induced by zinc using iron free isoforms of enzymes and expected as a consequence of the competitive uptake of metals; zinc excess hinders iron uptake (reviewed in Biotechnology advances 2013, 31:1292-1307).

Molybdenum cofactor sulfurase

Molybdenum cofactor sulfurase coding gene (MLOC_2170) was induced after 24 h of zinc treatment in one transcript and repressed in another. MCSU3 is involved in stress response likely through sulfation of molybdenum which is essential for xanthine dehydrogenase and aldehyde oxidase (ADO) enzymes (Plant Cell 2001, 13:2063-83).

Shaggy-related protein kinase alpha and 6-phosphogluconate dehydrogenase

In addition, shaggy-related protein kinase alpha-like gene (MLOC_52139) was induced over 400 times by iron treatment after 24 h compared to untreated plants and to 24 h after zinc treatment. It triggers the pentose phosphate pathway by providing NADPH and therefore, is involved in H_2O_2 removal by regulating the ascorbate-glutathione cycle (Plant Cell 2012, 24:3380-92). In agreement, the 6-phosphogluconate dehydrogenase gene (MLOC_25686) was induced in one transcript by iron after 6 h. By involving in the pentose-phosphate shunt, it catalyzes the oxidative decarboxylation of 6-phosphogluconate to ribulose 5-phosphate and CO2, with concomitant reduction of NADP to NADPH.

Flavonoid biosynthesis

Flavonoids are well-known antioxidants (Plant Physiol Biochem. 2013, 72:35-45). Iron repressed the gene dihydroflavonol 4-reductase (MLOC_65788). Dihydroflavonol 4-reductase coding genes XLOC_002759 and MLOC_65788 showed higher and lower expression levels in iron treated plants compared to zinc after 24 h, respectively. As another flavonoid biosynthesis player, chalcone synthase 1 (MLOC_74116) showed repression in iron treatment. The chalcone synthase 8 (MLOC_66998) was also repressed after 24 h of treatments.

Glutathione biosynthesis and degradation

The induction of glutathione synthetase by zinc and the induction of glutathione transferase F5 and the repression of glutathione degrading enzyme 5-oxoprolinase after iron treatment further evidenced the buildup of oxidative stress. Glutathione synthetase gene (MLOC_61045) was induced in one transcript by zinc after 24 h. Glutathione is involved in stress tolerance and chelation (Proc Natl Acad Sci U S A. 1987, 84:439-43; Int J Mol Sci. 2013, 14:7405-32). Glutathione also acts as a precursor for the synthesis of phytochelatins

(South African Journal of Botany 2010, 76:167-179). Glutathione transferase F5 (MLOC_68101) showed induction by iron when compared to the untreated samples. Glutathione transferases perform glutathione-dependent catalytic functions of conjugation and detoxification, reduction of hydroperoxides, compound isomerization, and non-catalytic transferase roles of binding and escorting of compounds (Genome Biol. 2002, 3:REVIEWS3004; Plant J. 2009, 58:53-68). We also found 5-oxoprolinase (*Oxp*1; MLOC_57018) repressed after 24 h of iron treatment. OXP1 catalyzes the cleavage of 5-oxo-L-proline, the second step in the glutathione degradation pathway in plants (Plant Physiol. 2008, 148:1603-13).

Ferritin

The gene coding for iron sequestering protein ferritin 1A (MLOC_69295) had higher expression levels in iron treated plants compared to zinc after 6 h and 24 h. Three transcript isoforms were detected for this gene; while the isoforms had low expression prior to the treatments, one was induced following the iron treatment. Ferritin works as an antioxidative stress component under excess iron (Plant J. 2009, 57: 400-412).

Lipid and protein damage

Oxidative stress is recognized by reactive radicals that target macromolecules including nucleic acids, proteins, and lipids. Different metal binding proteins had enhanced expressions. Together with thaumatin genes all were likely involved in membrane damage-repair. Finally, we noticed the induction of ABCG transporter genes involved in cuticle assembly and lignin biosynthesis.

Aquaporins

Channel inactivation has been proposed to explain the affected water permeability by heavy metals (Protoplasma. 2011, 248:663-671; Plant J. 1994, 6: 187-199). Interestingly, two aquaporin coding genes (MLOC_54419 and MLOC_8032) were repressed by iron. In addition, one tonoplast aquaporin coding gene (MLOC_14656) also switched transcripts when comparing iron with zinc. Therefore, less water permeability seems to be an adaptive strategy rather than a toxicity-mediated phenotype.

ACBP2

In *Arabidopsis*, the plasma membrane acyl-CoA-binding domain-containing protein ACBP2 (MLOC_62839) interacts with metallochaperone farnesylated protein 6 (FP6, with metal-binding motif M/LXCXXC) and lysophospholipase 2 and, is involved in phospholipid repair following lipid peroxidation induced by heavy metals and hydrogen peroxide (see Plant Mol Biol. 2003, 51:483-92; New Phytol. 2009, 181:89-102; Plant J. 2010, 62:989-1003; Plant Signal Behav. 2010, 5:1025-1027). We found one splicing isoform of the barley homologue for *Acbp*2 (MLOC_62839) induced strongly after 24 h of iron and zinc treatments. A second isoform was repressed at the same time.

Fp3

Barley *Fp*3 gene (MLOC_43497), a HMA superfamily member which codes for a predicted cell wall/cytosol localized protein, was also induced by iron after 24 h.

***Ubp*16**

The ubiquitin carboxyl-terminal hydrolase 16 (*Ubp*16; MLOC_58115) was induced after 24 h of zinc treatment. It is involved in heavy metal stress likely through interaction with metallochaperone heavy metal associated isoprenylated plant protein 27 (HIPP27 containing the metal-binding motif MXCXXC) which plays an important role in cadmium detoxification

and drought stress response (Plant Mol Biol. 2009 69:213-26; New Phytol. 2009, 181:89-102; Metallomics 2010, 2:556-64; Chem Soc Rev. 2011, 40:5282-92).

Thaumatins

We found thaumatin coding genes influenced after the treatments. Stress-related thaumatin proteins participate in membrane protection and repair. Thaumatins work through membrane permeabilization, β-glucan binding and degradation, inhibition of enzymes such as xylanases and α-amylase, as well as induction of apoptosis (BMC Plant Biol. 2011, 11:33). While in barley the thaumatin coding gene MLOC_12349 was repressed by iron and zinc, thaumatin-like protein PWIR2 coding gene (XLOC_102179) was induced by iron after 24.

Phosphatidylethanolamine

Phosphatidylethanolamine is required for cadmium resistance through functional maintenance of tonoplast (Mol Biol Cell. 2010, 21:443-55). Accordingly, phosphoethanolamine N-methyltransferase 1 (MLOC_66415) which converts phosphoethanolamine to phosphocholine, was repressed by the zinc after 6 h and by iron after 24 h. Additionally, the expression of ECPT-type aminoalcoholphosphotransferase (*Aapt*1, MLOC_53933) which results in the accumulation of phosphatidylethanolamine (Plant Mol Biol. 2010, 72:519-31) was induced after 24 h of the zinc treatment. These genes also had higher expression in zinc treated plants compared to iron.

ABCG family genes

ABCG transporters are involved in lipid metabolism in chloroplasts and endoplasmic reticulum (Arabidopsis Book 2013, 11:e0161). The ABCG family covers pleiotropic drug resistance transporters (PDRs) which shuttle secondary metabolites including heavy metals (FEBS Lett. 2006, 580:1123-30; Trends Plant Sci. 2008, 13:151-9). They can be induced to work against heavy metals and confer tolerance (FEBS Lett. 2003, 553:370-6; Biosci

Biotechnol Biochem. 2011, 75:1211-3). The putative chloroplast ABCG23 (MLOC_61784) coding gene had higher expression after 6 h in zinc treated plants compared to iron. Two other putative endoplasmic reticulum ABCG5 and ABCG25 (MLOC_66355 and MLOC_62985) coding genes were also repressed by the treatments. The Arabidopsis ABCG25 has recently been introduced as an ABA efflux transporter (Plant Physiol. 2014, 164:1587-92). By involving in the cuticle assembly (Plant Cell. 2011, 23:1958-70; Proc Natl Acad Sci U S A. 2011, 108:12354-9), the chloroplast/endoplasmic reticulum ABCG31 and ABCG32 coding genes (*Pdr*6 and *Pdr*4; MLOC_62487 and XLOC_036126) were upregulated by iron after 24 h. The chloroplast ABCG29/PDR1 coding gene (MLOC_5394) was also induced by iron after 24 h in one isoform while one other showed higher expression in the zinc treated samples compared to the iron after 6 h. In *Arabidopsis*, PDR1 participates in lignin biosynthesis (Curr Biol. 2012, 22:1207-12).

Methionine sulfoxide reductase

Our experiment also revealed the induction of the peptide methionine sulfoxide reductase gene (MLOC_57759) after 24 h of zinc treatment. The protein peptide methionine sulfoxide reductase reverses back the oxidized methionine residues in a proteome wide manner (Arch Biochem Biophys. 2002, 397:172-8). This indicates its important role in protecting cells against zinc-mediated oxidative damage.

Cellular protein processing machinery

We found huge perturbation in the cellular protein processing machinery including protein folding, ubiquitination, and degradation. Of these, E3 ubiquitin-protein ligase components were the most frequent type (S4 Fig) and involved not only in cellular cleaning of misfolded and damaged proteins but also in signal transduction through abundance-modulation of regulatory components (J Exp Bot. 2012, 63:599-616). Vacuolar protein-sorting-associated

protein 37 homolog 1-like isoform X4 coding gene (MLOC_59435) was induced in one of its isoforms by iron after 24 h. It is required for the sorting of endocytic ubiquitinated cargos as transmembrane surface transporters into prevacuolar compartments, i.e. multivesicular bodies (Trends Plant Sci. 2006, 11:115-23). The considerable expressional fluctuations in the proteome processing machinery genes is conceivably due to both the regulatory aspects and the cell cleaning functionality after oxidative damage. This builds another strong proof of wide metabolic changes within cells facing elevated levels of toxic metals.

Biotic stress responses

Iron plays an important role in host-pathogen interactions (see Arch Microbiol. 2011, 193:693-9; Biotechnology advances 2013, 31:1292-1307; Microbiol Res. 2014, pii: S0944-5013(14)00090-1). Therefore, iron should influence abiotic stress response mechanisms more effective than other metals. Accordingly, we found a much pronounced induction rate for defense related genes after iron treatment compared to the zinc treatment (S6 Table). More than 80% of the biotic stress related differentially expressed events showed elevated expression levels after iron treatment compared to less than 30% after the zinc treatment (S6 Table). Here, some of the responses are highlighted.

Germins

Germins are involved in response to various abiotic and biotic stresses likely through manganese superoxide dismutase activity (Int J Mol Sci. 2011, 12:7301-13; PLoS One 2013, 8:e61722). The germin coding gene XLOC_051490 was induced after 24 h of iron treatment.

Hypersensitive induced response *Hir*3 genes

Both genes were induced by iron after 24 h. HIRs promote the expression of resistance genes as well as the accumulation of both salicylic acid and hydrogen peroxide and, therefore, contribute to disease resistance (Mol Plant Pathol. 2007, 8:503-14; J Biol Chem. 2011, 286:31297-307).

The pathogen defense MLO protein coding genes

The *Mlo* gene MLOC_61466 was repressed by zinc after 24 h and the MLO coding gene MLOC_70290 had higher expression after iron treatment compared to zinc after 24 h.

Alpha-amylase inhibitor coding genes (MLOC_72278 and MLOC_65022)

They were induced by iron after 24 h.

Beta/alpha-hordothionin coding gene (MLOC_44740/MLOC_22225)

They were induced by both of the treatments. Thionins are small antimicrobial plant proteins (Critical Reviews in Plant Sciences 1994, 13:1-16).

Hydroxymethylglutaryl-CoA synthase-like gene MLOC_62700

Hydroxymethylglutaryl-CoA synthase had higher expression level after 24 h of iron compared to zinc. Hydroxymethylglutaryl-CoA synthase-like (HMG-CoA synthase) is an enzyme which catalyzes the formation of HMG-CoA as the second reaction in the mevalonate-dependent isoprenoid biosynthesis pathway. Plants secondary metabolites "terpenoids" are also involved in defense responses (Journal of Integrative Plant Biology 2007, 49:179-186).

Metal availability influences mobility of Mitochondria

Mitochondria have central role in cellular signaling and stress response and show function dependency on the dynamic state determined by mitochondria division, fusion, and motility (BMC Biology 2014, 12:34; BMC Biology 2014, 12:35). Even distribution of mitochondria is important for efficient targeting of metabolites and proper functionality of the mitochondria (see Hum Mol Genet. 2009, 18:R169-76; BMC Res Notes 2012, 5:505). Therefore, metal availability and toxicity dependency of mitochondria is expected. For example, cadmium enhances the fragmentation of mitochondria (Cell Death Dis. 2013, 4:e540). We found expression changes in the peripheral myelin protein 22 and Rho GTPase 1 coding genes accompanied with different calcium homeostasis factors illuminating the affected mitochondria movements and fission/fusion occurrence.

Peripheral myelin protein (PMP22)

A PMP22-like coding gene (XLOC_095381) had higher expression after 24 h of the iron treatment compared to zinc treatment. It is involved in mitochondrial movement in human (Hum Genet. 2000, 107:494-8; J Neurosci. 2007, 27:422-30).

Rho GTPase 1

Rho GTPase 1 is involved in mitochondria movement and under high calcium levels results in decreased mobility and increased fragmentation; increased cytosolic calcium level is expected under zinc treatment which mimics iron deficiency (Proc Natl Acad Sci U S A. 2008, 105:20728-20733; Front Cell Neurosci. 2013, 7:148; Biotechnology advances 2013, 31:1292-1307). Decreased mobility and increased fragmentation of mitochondria is therefore expected due to the induction of the Rho GTPase 1 coding gene (MLOC_61507) in the isoform with full Miro2 and partial Miro1 domains after 24 h of the zinc treatment.

Seed storage proteins are potential radical scavenging and metal chelating factors

Vesicle-associated membrane protein 727-like coding gene (MLOC_26339)

Showed higher expression by zinc compared to iron after 6 h. The *Arabidopsis* VAMP727 mediates the membrane fusion by which the traffic of storage proteins to the vacuole is handled (Plant Cell 2008, 20:3006-3021). Accordingly, seed storage protein coding genes showed higher expression levels in zinc treated plants compared to iron after 6 h.

Papain-like cysteine proteinase *Pap*-14

The gene *Pap*-14 (MLOC_25678) was repressed after 24 h of iron treatment and showed lower expression when comparing iron to zinc at the same time point. It codes for an ER-localized protein and may be involved in the hydrolysis of seed storage proteins (FEBS Lett. 1994, 351:31-34).

Vacuolar processing enzymes coding genes

The vacuolar processing enzyme 2b-like gene (XLOC_026648) switched transcript isoforms by iron. Vacuolar processing enzymes play an important role in the post-translational processing of seed proteins prior to storage and during germination (Plant Cell 2002, 14:2863-82; BMC Evol Biol. 2008, 8:198; Plant Cell Physiol. 2010, 51:38-46).

Upstream signaling factors of stress response

S3 Fig shows different upstream regulatory factors modulating the cellular stress response. In the following section, we explain the role of these components.

Salicylic acid signaling

Salicylic acid responding gene, Myb-related protein MYBAS2 coding gene (*Myb*17; MLOC_52439) was induced by zinc.

ABA signaling

The transcription factor MYB1R1 coding gene (MLOC_76271) was induced 24 h after iron treatment. As an ABA responsive gene, it confers drought and salt tolerance (see Plant Physiology 2010, 155:421-32). Another Myb-related gene (*Myb*AS1; MLOC_78504) was induced by zinc. *Myb*AS1 responds to abiotic stresses including dehydration, salt, cold, and wounding as well as salicylic acid and methyl jasmonic acid (see Plant Cell Reports 2012, 31:661-669).

Transcription factor Zip1

The transcription factor HBP-1a coding gene (*Zip*1) was induced after 6 h of iron treatment in one splicing isoform and was repressed in another isoform. We also found a zinc specific isoform with elevated expression levels after 24 h. *Zip*1 is involved in biotic and abiotic stress responses (Physiological and Molecular Plant Pathology 2009, 73:88-94).

Transcription factor Une12

The transcription factor *Une*12 (*Bhlh*59; MLOC_74728) was induced after 24 h of zinc treatment. BHLH59 is required for regulation of immune response (Development 2005, 132:603-14; Science 2011, 333:596-601).

Transcription factor Gte11and Npr4

Stress response factors *Gte*11 (MLOC_70538; Plant Mol Biol 2004, 54:549-69) and defense regulatory gene *Npr*4 (MLOC_51588; Plant J. 2005, 41:304-318; Plant J. 2006, 48:647-656) had lower expression levels in zinc treated sample compared to iron after 6 h.

Annexin D5

Iron treatment induced isoform switching in the positive regulator of stress response annexin D5-like gene (MLOC_63977; Plant Cell Physiol. 2010, 51:1499-514; Plant Physiol. Biochem. 2006, 44:13-24, J. Exp. Bot. 2012, 63:5593-606). It was also induced by zinc.

Dehydration-Induced 19

Arabidopsis dehydration-induced 19 (*Di*19) gene family encodes a novel type of Cys2/His2 zinc-finger protein implicated in ABA-independent dehydration, high-salinity stress, and light signaling pathways (see Plant Molecular Biology 2006, 61:13-30). We found DI19-5 gene (MLOC_4058) repressed after 24 h of the treatments.

GsSRK

A G-type lectin S-receptor-like serine/threonine-protein kinase At2g19130-like gene (MLOC_34521) was also induced in one isoform by iron treatment after 24 h. The *Arabidopsis* GsSRK coding gene (At1g11410) is a stress-tolerance factor ensuring higher yield and chlorophyll content when overexpressed under salt stress (see J. Plant Physiol. 2013, 170:505-15). A second GsSRK gene (MLOC_57343) was also found induced after 24 h of iron treatment.

Protein phosphatase 2Cs (PpCs)

The protein phosphatase 2Cs have been implicated as negative modulators of protein kinase pathways involved in diverse environmental stress responses (BMC Genomics 2008, 9:550). We found protein phosphatase 2Cs coding genes induced by both the zinc and iron. The putative protein phosphatase 2C 67-like gene (XLOC_069405) and one isoform of the protein phosphatase 2C06 gene (MLOC_52176), 2C39 (MLOC_56095), and 2C45 (MLOC_54057) were induced after 24 h of iron and zinc treatments. The protein phosphatase 2C47 gene (MLOC_38045) was repressed by iron after 24 h.

Mekk1

The Mitogen-activated protein kinase kinase kinase 1 (MLOC_13593) showed lower expression after 24 h in iron treated plants compared to zinc. MEKK1 is involved in the innate immune MAP kinase signaling cascades (MEKK1, MKK4/MKK5 and MPK3/MPK6; Trends in Plant Science 2011, 16:300-309).

Itn1

The protein Increased tolerance to NaCl (MLOC_64183) which negatively regulates ROS production (see Plant J. 2008, 56:411-22), was induced by zinc after 24 h compared to 6 h.

Mpv17-like

As second level of defense mechanisms, cells try to scavenge the produced ROS in order to alleviate ROS-mediated cellular damages. We also found the protein Mpv17-like coding gene (MLOC_34722) repressed after 24 h of zinc treatment. Mpv17-like is a peroxisomal membrane protein which down-regulates expression of the glutathione peroxidase and catalase genes (Biochem Biophys Res Commun. 2006, 344:948-54).

HSPs

The polyamine spermine protects *Arabidopsis* from heat stress-induced damage by increasing the expression of heat shock-related genes *Hsp*101, *Hsp*90, *Hsp*70, and *Hsp*17.6 (Transgenic Res. 2013, 22:595-605). Gene involved in protein folding and chaperoning including 17 kDa class I small heat shock protein coding gene (MLOC_44536) and *DnaJ*-like (MLOC_38972) had lower and higher expression levels in zinc treated sample compared to iron after 6 h. The *Hsp*90 (MLOC_10369) and *Hsp*70 (MLOC_67581) genes showed higher and lower expression levels after 6 h of zinc treatment compared to iron. In contrast, the sacsin-like gene (MLOC_14650) was repressed by iron and had lower expression compared to zinc after 24 h. This gene codes a HSP70 co-chaperone in human (Hum Mol Genet. 2009, 18:1556-65).

Heat shock transcription factor *A2*c (*HsfA*2c; MLOC_498.1) was repressed by zinc after 24 h. In contrast, the heat stress transcription factor A1 (*HsfA*1; MLOC_67043) was induced by zinc after 24 h. HSP90 and HSP70 are involved in stress response through regulation of the heat shock transcription factors A1, A2, and B1 (J Biol Chem. 2007, 282:37794-804; Plant Cell 2011, 23:741-55). HSFA1 acts upstream to induce the expression of the other factors as *HsfB*1 and *HsfA*2 (Plant Cell Physiol. 2011, 52:933-45). The inhibition of 26S proteasome function and/or Hsp90 activity is involved in the induction of *HsfA*2 expression in response to oxidative stress (Plant Cell Physiol. 2010, 51:486-96). In addition, HSP90 controls the abundance of HSFA2 and HSFB1 negatively (Plant Cell 2011, 23:741-55). The activity and DNA binding capacity of HSFA1 and the activity of HSFB1 are also repressed by HSP70 (Plant Cell 2011, 23:741-55). HSP70s repress the DNA binding and the activity of the HSFA1, an upstream transcription factor of HSFA2.
