# Supplementary material for: Deciphering Mineral Homeostasis in Barley Seed Transfer Cells at Transcriptional Level
Source: PLoS One. 2015 Nov 4;10(11):e0141398. doi: 10.1371/journal.pone.0141398 (PMC4633283; doi:10.1371/journal.pone.0141398)
Supplement: S2 Text — (PDF) [file pone.0141398.s020.pdf]

Deciphering Mineral Homeostasis in Barley Seed Transfer Cells at Transcriptional Level

Behrooz Darbani[1,2¶]*, Shahin Noeparvar[1¶], Søren Borg[1]*

[1] Department of Molecular Biology and Genetics, Research Centre Flakkebjerg, Aarhus University, Forsøgsvej 1, 4200 Slagelse, Denmark
[2] Department of Plant and Environmental Sciences, University of Copenhagen, Frederiksberg, Denmark
* Corresponding authors
Email: behroozdarbani@gmail.com (BD), soren.borg@mbg.au.dk (SB)

S2 Text

Excess zinc and iron affect photosynthesis and respiration

(Accession numbers of the genes are available in S2 and S4 Files)

Our data provides additional evidence on the importance of iron in photosynthesis as recently emphasized in (Trends Plant Sci. 2015, 20:33-40). The tetrapyrrole biosynthesis pathway was enhanced by the treatments. Tetrapyrrole is utilized either by ferrochelatase for heme biosynthesis or by chloroplastic Mg-chelatase for chlorophyll biosynthesis (Arabidopsis Book. 2011, 9:e0145). Catalyzing the third step in tetrapyrrole biosynthesis pathway (Arabidopsis Book. 2011, 9:e0145), the aminolevulinic acid dehydratase (*Alad*) was induced by iron and zinc (S5 Fig.). The chloroplastic ferrochelatase-2 had also lower expression after iron treatment compared to zinc. This can divert tetrapyrrole to heme biosynthesis after zinc triggered iron deficiency and enhance chlorophyll biosynthesis. The chlorophyll b reductase NOL had properly higher expression level after iron treatment compared to zinc (S5 Fig.). NOL has chlorophyll refreshment action by participating in the degradation of chlorophyll complexes (J Biol Chem. 2009, 284:17449-17456). Accordingly, the RuBisCO activase and RuBisCO large subunit *Rbcl*-1 were induced by iron. In contrast, the photosystem I P700 apoprotein A1 and A2, photosystem II CP43 genes, photosystem II D1 and 2, and MCB1

coding genes showed lower expression levels by zinc compared to iron. Hv*Mcb*1 codes for a transcription factor promoting the genes coding for chlorophyll a/b binding proteins of photosystem II (Plant Mol Biol. 2003, 52:447-462). These expressional rearrangements are consistent with the high cellular heme-demand/decreased cellular heme content due to the zinc-mediated iron deficiency after 24 h. Accordingly, sugar transportation was enhanced by iron and restrained by zinc. The *Sweet*4 gene was repressed by zinc and *Sweet*13 and 11 had lower expression levels in zinc-treated plants compared to iron. *Sweets* mediate low-affinity influx and efflux of sugar (Nature 2010, 468:527-532).

Iron and zinc repletion affect respiration. We found a putative mitochondrial chaperone BCS1-B, necessary for the assembly of mitochondrial respiratory chain complex III (J Cell Sci. 2008, 121:2588-2600), repressed by both iron and zinc. The electron chains complex I subunit NADH dehydrogenase iron-sulfur protein 2 was also repressed by zinc. Additionally, the respiratory chain complex I subunit LYR family of Fe/S cluster biogenesis protein had lower expression in zinc-treated plants compared to iron. As we discussed it before (Biotechnol Adv. 2013, 31:1292-1307), these changes may alleviate the negative effects of iron depletion on mitochondrial electron transfer chains. The zinc treatment triggered strong repression in the cytosolic aconitase coding gene. This likely reinforced respiration through Krebs cycle by providing less citrate to ammonium assimilation (see J Exp Bot. 2002, 53:905-916) which is in line with the impaired nitrate uptake under iron deficiency (see Biotechnol Adv. 2013, 31:1292-1307). By recalling the increased cytosolic calcium level after zinc treatment which mimics iron deficiency (Biotechnol Adv. 2013, 31:1292-1307), the zinc-mediated induction of Rho GTPase 1 indicates the importance of proper cellular levels of zinc and iron for mitochondrial functionality. Rho GTPase 1 is involved in mitochondrial movement and under high calcium levels results in decreased mobility and increased

fragmentation (Front Cell Neurosci. 2013, 7:148; Proc Natl Acad Sci U S A. 2008, 105:20728-20733). Our data provides additional evidence to the interplay between iron and oxygen during the evolution (Biotechnol Adv. 2013, 31:1292-1307) concluding a limited aerobic metabolism under excess iron levels to ensure less oxidative stress. Accordingly, the replenishment mechanism of Krebs cycle (the activation of pyruvate dehydrogenase by inositol 1,4,5-trisphosphate receptor dependent calcium release, Cell 2010, 142:270-283) was likely affected by iron since the repression of *pBI*-1 interfere with the activity of inositol 1,4,5-trisphosphate receptor. The expression of cytosolic phosphoenolpyruvate carboxylase *Pepc*2 which enhances respiration through Krebs cycle (J Exp Bot. 2011, 62:3049-3059) was also repressed by iron. In view of the cytosolic calcium dependent activation of PEPC (Plant J. 2000, 23:497-506), *Pepc*2 repression was in harmony with the expected reduction in cytosolic calcium. Dampening Krebs cycle, the putative chloroplast/mitochondrial pyruvate dehydrogenase and chloroplast acetyl-COA synthetase coding genes had lower and higher expression levels, respectively, in iron-treated plants compared to zinc after 6 h. We also found a gene coding for a putative cytosolic/chloroplastic NADP-malic enzyme 3 with higher expression level in iron-treated plants compared to zinc. It works against Krebs cycle through mitochondrial-to-cytosol shuttle of malate (see Plant Physiol. 1988, 87:109-115). A putative mitochondrial/chloroplastic malate to oxaloacetate converting enzyme coding gene, malate dehydrogenase 2 which works against the pyruvate-malate shuttle, had properly higher expression levels in two transcripts when comparing zinc with iron. Further evidence came from iron triggered suppression of the genes coding for mitochondrial electron transfer chains including cytochrome *c* oxidase subunit 1 and 3, NADH dehydrogenase subunit 4L, and apocytochrome *b*.
